# Metabolomics and cancer preventive behaviors in the BC Generations Project

**DOI:** 10.1038/s41598-021-91753-8

**Published:** 2021-06-08

**Authors:** J. Qi, J. J. Spinelli, T. J. B. Dummer, P. Bhatti, M. C. Playdon, J. Olin Levitt, B. Hauner, S. C. Moore, R. A. Murphy

**Affiliations:** 1 School of Population and Public Health, University of British Columbia, Vancouver, BC Canada; 2 Cancer Control Research, BC Cancer, 2-107, 675 W 10th Ave, Vancouver, BC V5Z 1L3 Canada; 3 Department of Nutrition & Integrative Physiology, University of Utah, Salt Lake City, UT USA; 4 Cancer Control and Population Sciences Program, Huntsman Cancer Institute, University of Utah, Salt Lake City, UT USA; 5 Division of Cancer Epidemiology & Genetics, National Cancer Institute, Bethesda, MD USA

**Keywords:** Cancer epidemiology, Cancer prevention, Risk factors, Metabolomics

## Abstract

Metabolomics can detect metabolic shifts resulting from lifestyle behaviors and may provide insight on the relevance of changes to carcinogenesis. We used non-targeted nuclear magnetic resonance to examine associations between metabolic measures and cancer preventive behaviors in 1319 participants (50% male, mean age 54 years) from the BC Generations Project. Behaviors were dichotomized: BMI < 25 kg/m^2^, ≥ 5 servings of fruits or vegetables/day, ≤ 2 alcoholic drinks/day for men or 1 drink/day for women and ≥ 30 min of moderate or vigorous physical activity/day. Linear regression was used to estimate coefficients and 95% confidence intervals with a false discovery rate (FDR) of 0.10. Of the 218 metabolic measures, 173, 103, 71 and 6 were associated with BMI, fruits and vegetables, alcohol consumption and physical activity. Notable findings included negative associations between glycoprotein acetyls, an inflammation-related metabolite with lower BMI and greater fruit and vegetable consumption, a positive association between polyunsaturated fatty acids and fruit and vegetable consumption and positive associations between high-density lipoprotein subclasses with lower BMI. These findings provide insight into metabolic alterations in the context of cancer prevention and the diverse biological pathways they are involved in. In particular, behaviors related to BMI, fruit and vegetable and alcohol consumption had a large metabolic impact.

## Introduction

The global burden of cancer is substantial, with an estimated 18.1 million new cancer cases and 9.6 million cancer deaths in 2018 alone^1^. Over the coming decades, this burden is projected to grow substantially^2^. In the words of Christopher Wild, former director of the International Agency for Research on Cancer (IARC) “we cannot treat our way out of the cancer problem”^3^. Modifiable factors contribute substantially to the overall risk of cancer. Increased body weight, poor diet, physical inactivity and alcohol consumption accounted for approximately 18% of cancer cases in the United States in 2014, the highest contributing factors other than cigarette smoking^4^. Recent estimates from a Canadian population, suggest 34% of cancer in men and 33% of cancers in women are attributable to preventable risk factors^5^.

A number of guidelines and recommendations for cancer prevention have been put forth from government and nonprofit health organizations including the American Cancer Society and the World Cancer Research Fund/American Institute for Cancer Research^6,7^. Recommendations include maintaining body weight within the healthy range and avoiding weight gain in adulthood, being physically active at levels similar to national guidelines, limiting sedentary time, eating a healthy diet which includes wholegrains, fiber, fruits and vegetables, limited red meat, little if any processed meat, limited fast foods and sugary drinks and limiting or avoiding consumption of alcohol. Despite recommendations, few individuals engage in preventive behavior. For example, epidemiologic studies have reported 11% of women meet recommendations for fruit and vegetable consumption and less than 1% of men and women meet all preventive recommendations^8,9^. A greater understanding of mechanisms linking lifestyle behaviors and cancer is important to identify individuals who may be at greater risk, identify or refine strategies for preventive efforts and provide insight on biological processes that could be targets for new preventive approaches.

Metabolomics, the study of small molecules that participate in metabolism provides an integrated view of upstream genetic, transcriptomic and proteomic variation. As such, metabolomics can provide detailed characterization of metabolic phenotypes and corresponding alterations in metabolism that may precede disease. For example, a number of studies have examined metabolic changes associated with obesity^10–12^. Findings suggest obesity is associated with elevated branched-chain amino acid (BCAA), and saturated fatty acid metabolites, suggestive of insulin resistance as well as elevated levels of inflammation and sex hormone-related metabolites. Prior pilot research from our group reported several notable differences in metabolite profiles between people (n = 120) who did and did not follow recommendations for body weight, physical activity and alcohol consumption. For example, not meeting recommendations was associated with lipid metabolite profiles indicative of poor metabolic health including lower levels of high-density lipoprotein (HDL) metabolites, and higher levels of low density lipoprotein (LDL) metabolites^13^. The aim of the current study was to expand upon our prior effort to study associations between cancer preventive behaviors and metabolic profiles and to determine relevance of metabolic associations with cancer-related pathways in a much larger sample of Canadian adults.

## Results

A sample from one participant failed during metabolomics analysis, and thus the study included 1319 participants. Characteristics of these participants are shown in Table 1. On average participants were 54 years of age, 50% were men, 44.4% had less than a university-level education, 34.4% had a university level education, 20.6% had a graduate degree, and nearly 82% were white. With respect to annual household income, 40.0% reported income > $100,000. The mean (SD) BMI was 26.5 (4.76) kg/m^2^, approximately 5% reported prevalent diabetes, 1% reported prevalent heart disease as defined by a history of stroke or myocardial infarction and 30.2% reported a family history of cancer. Aside from half of the population being men, the demographics align with the overall BC Generations Project (BCGP) cohort^14^.

**Table 1:** Select participant characteristics (N = 1319).

Characteristic	
Age (years), mean (SD)	54.4 (9.03)
Men, N (%)	658 (49.9)
**Education, N (%)**	
< University	586 (44.4)
University	454 (34.4)
Graduate degree	272 (20.6)
Missing	7 (0.53)
**Ethnicity, N (%)**	
White	1,079 (81.8)
Non-white	188 (14.3)
Missing	52 (3.94)
**Income, N (%)**	
< $50,000	230 (17.4)
$50,000–$100,000	485 (36.8)
> $100,000	527 (40.0)
Missing	77 (5.84)
BMI (kg/m^2^), mean (SD)	26.5 (4.76)
BMI < 25 kg/m^2^, N (%)	559 (42.4)
Servings of fruits and vegetables, mean (SD)	5.20 (2.60)
≥ 5 servings of fruits and vegetables, N (%)	726 (55.0)
Moderate to vigorous physical activity, minutes/wk, mean (SD)	749 (737)
≥ 30 min moderate to vigorous physical activity on ≥ 5 days/wk, N (%)	562 (42.6)
Alcohol consumption, drinks/d, mean (SD)	0.70 (0.50)
≤ 2 drinks/d (men), ≤ 1drink/d (women), N (%)	1057 (80.1)
Prevalent diabetes, N (%)	71 (5.38)
Prevalent heart disease, N (%)	15 (1.14)
Family history of cancer, N (%)	798 (30.2)

Heart disease defined as self-reported stroke or heart attack.

**Figure 1:**
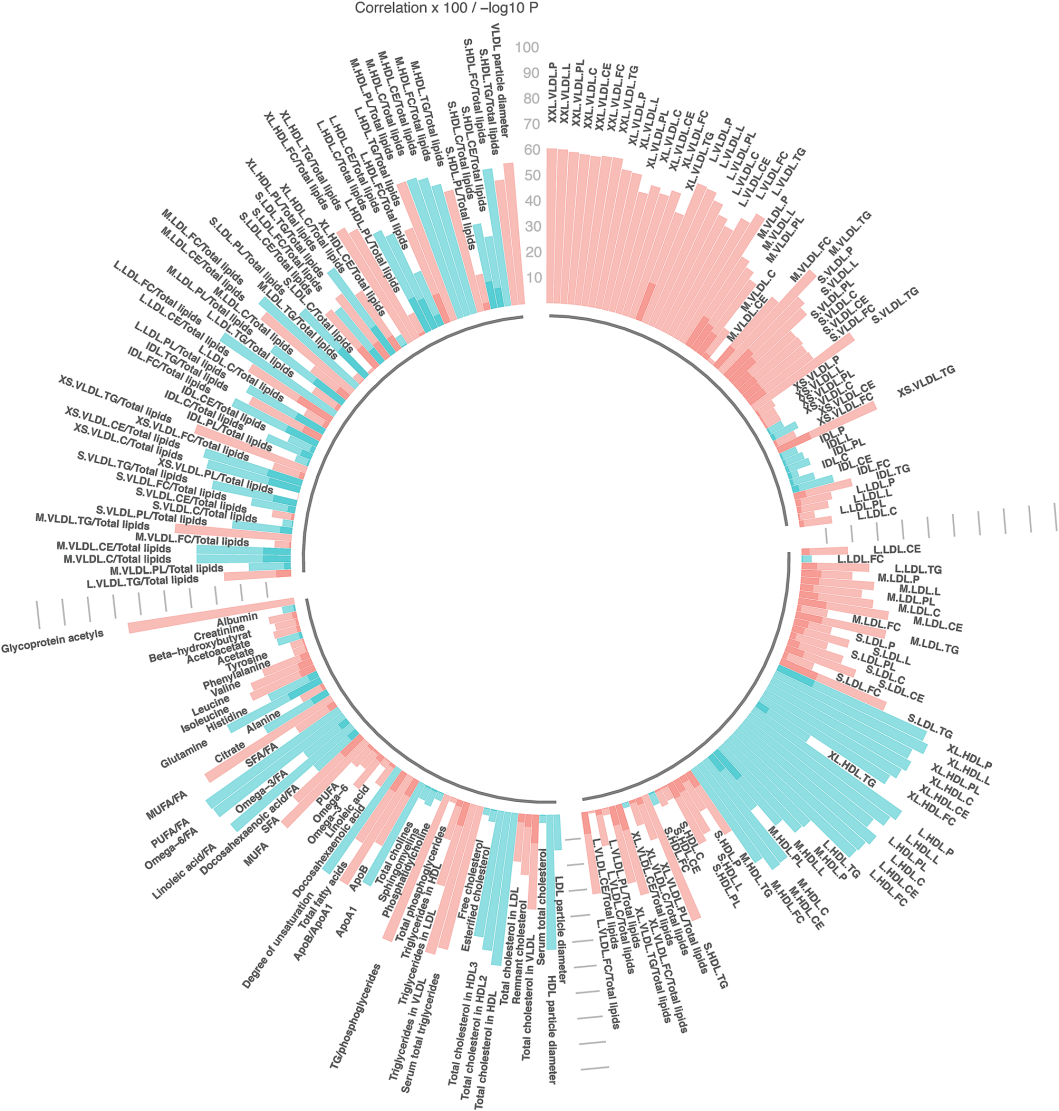
Linear regression, comparing metabolic measures with BMI categorized according to cancer preventive recommendations (BMI < 25 kg/m^2^ or ≥ 25 kg/m^2^ (referent). Models adjusted for age, sex and study (pilot or larger sample). Significance determined at p < 0.05 and q < 0.10. Bar heights represent the magnitude of the beta-coefficient from linear regression. Red bars indicate positive betas and blue bars indicate negative betas. Full names of metabolic measures listed are provided in the Supplemental Tables. Figure generated with R version 4.0.3^44^.

**Figure 2:**
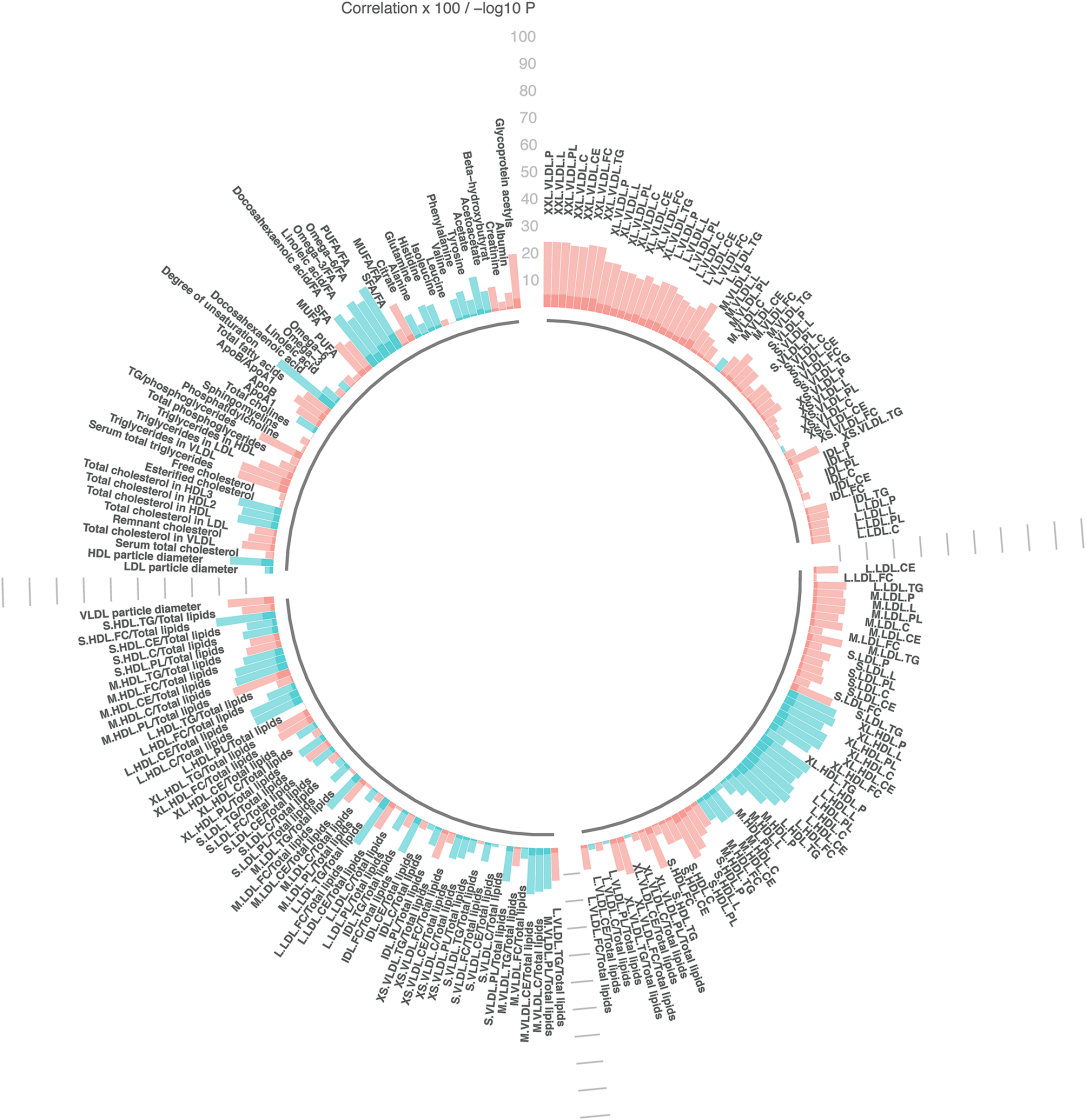
Linear regression, comparing metabolic measures with fruit and vegetables categorized according to cancer preventive recommendations (fruit and vegetable consumption < 5 servings/day (referent) or ≥ 5 servings per day). Models adjusted for age, sex and study (pilot or larger sample). Significance determined at p < 0.05 and q < 0.10. Bar heights represent the magnitude of the beta-coefficient from linear regression. Red bars indicate positive betas and blue bars indicate negative betas. Full names of metabolic measures listed are provided in the Supplemental Tables. Figure generated with R version 4.0.3^44^.

**Figure 3:**
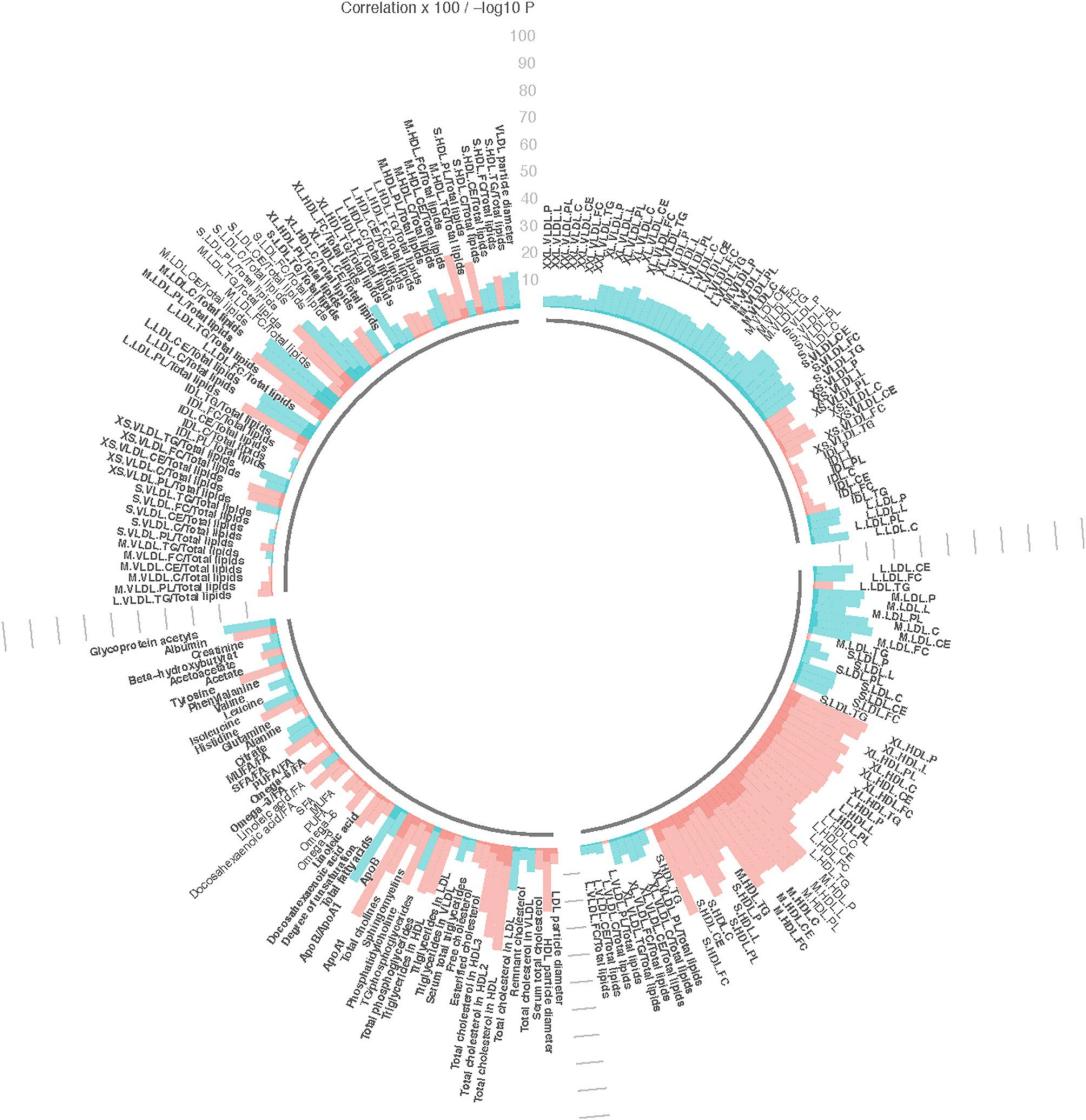
Linear regression, comparing metabolic measures with alcohol consumption categorized according to cancer preventive recommendations (no more than 2 drinks/day for men or 1 drink/day for women (referent) or > 2 drinks/day for men or > 1 drink/day for women). Models adjusted for age, sex and study (pilot or larger sample). Significance determined at p < 0.05 and q < 0.10. Bar heights represent the magnitude of the beta-coefficient from linear regression. Red bars indicate positive betas and blue bars indicate negative betas. Full names of metabolic measures listed are provided in the Supplemental Tables. Figure generated with R version 4.0.3^44^.

**Figure 4:**
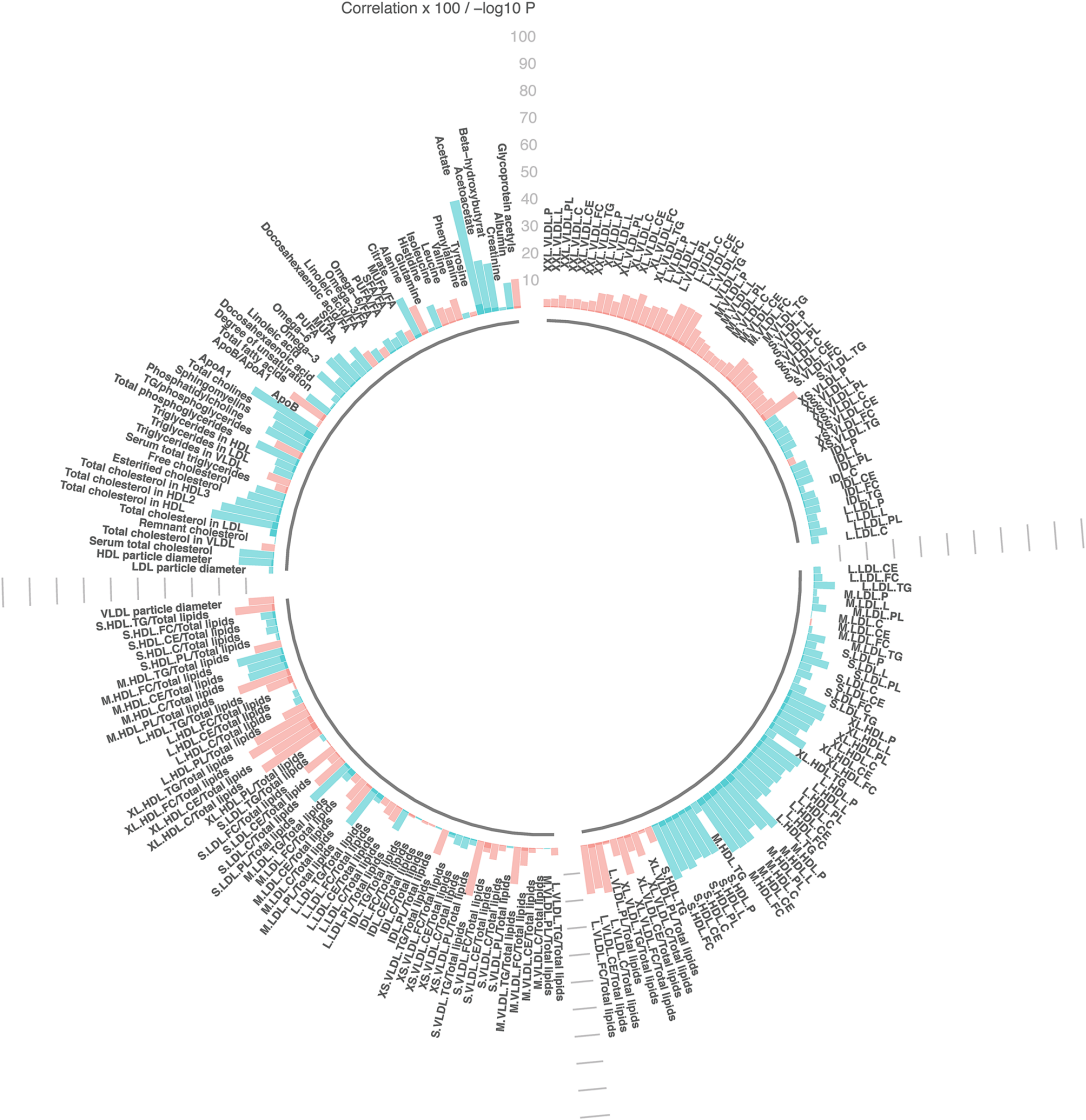
Linear regression, comparing metabolic measures with physical activity categorized according to cancer preventive recommendations (at least 30 min of moderate or vigorous physical activity on 5 or more days of the week (referent) or < 30 min of moderate or vigorous physical activity on 5 or more days of the week). Models adjusted for age, sex and study (pilot or larger sample). Significance determined at p < 0.05 and q < 0.10. Bar heights represent the magnitude of the beta-coefficient from linear regression. Red bars indicate positive betas and blue bars indicate negative betas. Full names of metabolic measures listed are provided in the Supplemental Tables. Figure generated with R version 4.0.3^44^.

Associations between lifestyle behaviors categorized according to cancer prevention guidelines and metabolic measures are shown in Figs. 1, 2, 3, 4 and in Supplemental Tables 5–8. A large number of metabolic measures were associated with each health behavior at the a-priori determined significance level (p < 0.05 and q < 0.10) including 173 with BMI (Fig. 1), 103 with fruit and vegetable intake (Fig. 2) and 71 with alcohol consumption (Fig. 3). Only 6 metabolic measures were associated with moderate to vigorous physical activity (Fig. 4, Supplemental Table 8). These included a ketone body (acetate, coefficient = 0.43, p = 0.0002), total cholines (coefficient = 0.16, p = 0.0009), four lipoprotein measures: medium HDL particles, coefficient = 0.25, p = 0.003, free cholesterol in medium HDL, coefficient = 0.23, p = 0.004, total lipids in medium HDL, coefficient = 0.23, p = 0.004, and total cholesterol in LDL, coefficient = 0.23, p = 0.005. Additional adjustment for demographic (sex, education, income, ethnicity) and health variables (diabetes, heart disease) in models did not appreciably impact associations between cancer preventive behaviors and metabolic measures. Similar associations were also observed with mutual adjustment for lifestyle behaviors within a given model (data not shown).

Of the metabolic measures associated with an overweight/obese BMI, 13 were fatty acids, three were apolipoproteins (ApoA1, ApoB and the ratio of ApoB to ApoA1), eight were amino acids including inverse associations with BCAAs isoleucine, valine and leucine, two were fluid-balance related (creatinine and albumin), one was inflammation-related (glycoprotein acetyls), and the balance were glycerides, phospholipids and lipoprotein measures. Specific metabolic measures, their coefficients and p-values are shown in Fig. 1 and Supplemental Table 5. The largest coefficients and most significant associations were observed for subclasses of HDL, for example, large HDL particles (coefficient = 0.71, p < 10^−11^) and cholesterol in large HDL (coefficient = 0.69, p < 10^−11^). The largest negative coefficients were observed for glycoprotein acetyls (coefficient = − 0.66, p < 10^−11^), the ratio of triglycerides to phosphoglycerides (coefficient = − 0.61, p < 10^−11^) and lipoprotein measures, particularly larger average size of very low density lipoproteins (VLDL) subclasses.

Of the 103 metabolic measures associated with fruits and vegetables, 10 were fatty acids, one was an apolipoprotein (the ratio of ApoB to ApoA1), one was inflammation-related (glycoprotein acetyls), four were trigylcerides, and one was a ketone body (acetate) with the balance comprised of cholesterol, and lipoprotein subclasses. The coefficients and p-values are shown in Fig. 2 and Supplemental Table 6. Metabolic measures with the largest coefficients and most significant associations with fruit and vegetable consumption were fatty acid related measures including the degree of fatty acid unsaturation (coefficient = 0.26, p = 5.54 × 10^−7^), the ratio of polyunsaturated fatty acids to total fatty acids (coefficient = 0.26, p = 1.05 × 10^−6^) and ratio of omega-3 fatty acids to total fatty acids (coefficient = 0.24, p = 8.93 × 10^−6^).

The metabolic measures associated with alcohol consumption were predominately subclasses of lipoproteins or cholesterol metabolites (N = 63), two were apolipoproteins, three were glycerides and phospholipids, 3 were fatty acids, 1 was inflammation-related, two were amino acids and one was a fluid balance related. The largest coefficients and most significant associations between metabolic measures and alcohol consumption were observed for ApoA1 (coefficient = − 0.42, p = 6.30 × 10^−10^), and various HDL metabolic measures e.g. lipids in medium HDL (coefficient = − 0.41, p = 4.12 × 10^−9^), as shown in Fig. 3 and Supplemental Table 7.

### Consistency across behaviors

No metabolic measures were commonly associated with all lifestyle behaviors. Thirty-nine metabolic measures were associated with three of the four lifestyle behaviors including the ratio of ApoB to ApoA1, the ratio of docosahexaenoic acid (DHA) to total fatty acids, glycoprotein acetyls, the ratio of triglycerides to phosphoglycerides and various lipoprotein measures. Notably, the association patterns for BMI and fruit and vegetable consumption were generally consistent with respect to direction (both positive or both negative) but were opposite of associations with alcohol consumption. For example, the coefficient of glycoprotein acetyls for fruits and vegetables and BMI were − 0.20 and − 0.66 while for alcohol consumption it was 0.19.

### Metabolic pathways determined by GGM

**Figure 5:**
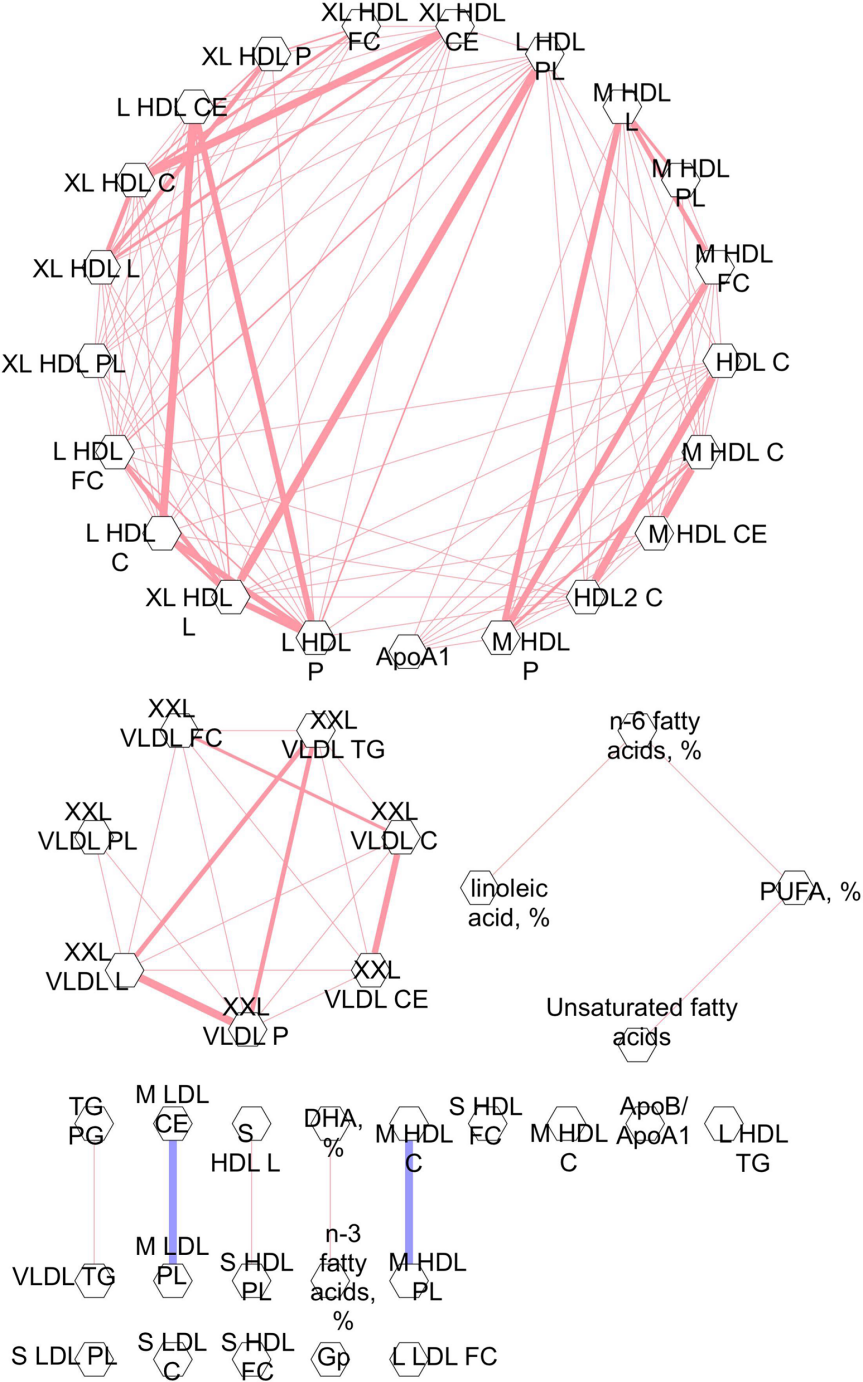
Gaussian Graphical Model of the top 25 metabolic measures correlated with alcohol consumption, BMI and fruits and vegetables categorized according to cancer preventive recommendations. The width of the line represents the strength of the correlation. Blue lines represent inverse correlation. Red lines represent positive correlations. Metabolic measures that are connected represent conditional correlations that were in the top 10%. Apo = apolipoprotein, C = cholesterol, CE = cholesterol ester, DHA = docosahexaenoic acid, FC = free cholesterol, L = lipids if suffix i.e. XL HDL L or L = large if prefix i.e. L HDL C, M = medium, P = particle, PG = phosphoglycerides, PL = phospholipid, PUFA = polyunsaturated fatty acids, S = small, TG = triglyceride, XXL = extremely large, XL = very large. Full names of metabolic measures listed are provided in the Supplemental Tables. Figure generated with the Cytoscape app within Metscape^48^.

The interrelationships of the top 25 BMI, fruit and vegetable and physical activity-associated metabolic measures are shown in the GGM (Fig. 5). Due to overlapping metabolic measures between behaviors, 49 metabolic measures are included in the GGM. Twenty HDL metabolic measures clustered with apolipoprotein A1 (the major protein component of HDL) into a single large interrelated network. Metabolic measures in this cluster were primarily associated with BMI, with particularly strong associations for the large HDL metabolic measures. Seven very large VLDL metabolic measures clustered into a network and were among those most strongly associated with fruit and vegetable consumption. Four fatty acid metabolic measures formed a separate cluster and were primarily associated with fruit and vegetable consumption and BMI. Additional small clusters included lipoprotein subclasses, glycerides and phospholipids, fatty acids and cholesterols. The remaining nine metabolic measures were not related to any of the other metabolic measures with conditional correlation in the top 10% and above, and may represent distinct phenomena.

## Discussion

This study of 1319 participants in the BCGP cohort, determined metabolic signatures associated with four major modifiable behaviors recommended for cancer risk reduction. Out of the 218 metabolic measures captured by NMR, 173, 103, and 71 were associated with BMI, fruit and vegetable intake and alcohol, respectively. Metabolic signatures associated with lifestyle behaviors spanned lipoproteins, amino acids, fatty acids, inflammation, fluid balance, apolipoproteins, glycerides, and phospholipids. Associations confirm previously identified metabolite-behavior associations such as ApoA-1 and alcohol consumption^15^, and associations previously unreported, such as glycoprotein acetyls, a marker of inflammation with fruit and vegetable consumption. Glycoprotein acetyls was also among the metabolic measures most strongly associated with BMI and fruit and vegetables consumption, as were subclasses of HDL with BMI and consumption of alcohol. Many of the metabolic measures identified are of prominent importance in molecular pathways associated with cancer development, as well as other chronic diseases such as diabetes and cardiovascular disease that share common behavioral risk factors and underlying mechanisms with cancer.

Our findings for BMI-related metabolic measures align with prior studies^9–11^. Established relationships have been reported between amino acids and BMI, and particularly for BCAA metabolites. Elevated BCAA metabolites have been consistently associated with increased risk of diabetes^16,17^, which is hypothesized to reflect excess protein breakdown and release of amino acid breakdown products as a result of insulin resistance^18^. A case control study of postmenopausal breast cancer reported that BCAA metabolites were associated with BMI and also with increased breast cancer risk^19^. Evidence supports a synergistic relationship between obesity-related insulin resistance and diabetes with cancer^20^, and thus, these amino acids are biologically relevant for cancer risk. Alternatively, acetyl-CoA production and protein acetylation pathways are significantly altered in obesity, and hypothesized to contribute to the link between obesity and cancer by enhancing lipid accumulation and inflammation^21^. Amino acid metabolism, along with other metabolic pathways such as fatty acid oxidation, ketogenesis and lipid synthesis are integrally connected with acetyl-CoA metabolism. Elevated metabolic measures of amino acids, citrate, glycoprotein acetyls and fatty acids including polyunsaturated fatty acids, among participants with overweight/obese BMI in our study support the notion of altered acetyl-CoA production and protein acetylation, although these markers need to be explored in future prospective studies to determine the relevance as mediators of obesity and cancer risk.

Lipid metabolic measures, including HDL particles, were most commonly and strongly associated with BMI. Although this in part reflects the lipid-heavy platform, a prior study which utilized a different metabolomics platform similarly reported that lipids featured prominently as part of the BMI metabolome^10^. Of particular interest, LDL and HDL lipoprotein metabolic measures were inversely and positively associated with normal BMI, respectively. The GGM models depicted a correlated distinct network of HDL metabolic measures and apolipoprotein A1, while metabolic measures of HDL lipoproteins formed small clusters or were not correlated to other measures. These main cholesterol transporters have been implicated in carcinogenesis and particularly breast cancer which is strongly linked with excess body fat. Experimental models suggest cholesterol transporters may drive carcinogenesis via oxidative modification of lipoproteins and activation of inflammation-related pathways, leading to cell proliferation, migration, and inhibition of apoptosis^22^. Cohort and case–control studies suggest inverse associations between HDL cholesterol and breast cancer risk^23^ and positive associations between LDL cholesterol and breast cancer risk^24,25^. As a result, cholesterol lowering drugs and apolipoprotein A-1 mimetics have emerged as possible therapeutics for breast cancer^23^.

Similar to BMI, associations were observed between fruit and vegetable consumption and metabolic measures of lipoprotein subclasses, however, the largest coefficients were observed for polyunsaturated fatty acids, omega-3 fatty acids and unsaturated fatty acids, which were positively associated with fruit and vegetable consumption. As may be expected, given their biological and chemical properties, these metabolic measures formed a small correlated cluster. Our findings align with those from a large cohort of British men and women which examined metabolite profiles associated with diet quality evaluated as adherence to the alternative healthy eating index (AHEI). The AHEI is scored according to intake of foods and nutrients that can prevent chronic disease. Fruit and vegetable consumption contribute up to 20 points out of a total score of 87.5^26^. They reported a higher AHEI score, indicating a healthier diet was associated with lower glycoprotein acetyls, smaller size of VLDL particles, larger size of HDL particles, the degree of fatty acid unsaturation, higher polyunsaturated fatty acids including omega-3 fatty acids and DHA. Similar inverse associations were observed for serum total triglycerides and monounsaturated fatty acids. Despite the considerable overlap, we also observed associations unique to this study for valine and acetate (positive associations) and the ratio of ApoB1/ApoA1 (negative association). Each of these metabolic measures were also associated with at least one other behavior and it is thus possible that they are markers of overall lifestyle and not specifically dietary intake, although all three associations remained statistically significant in models mutually adjusted for physical activity, alcohol consumption and BMI.

Metabolic profiles of adherence to cancer prevention alcohol guidelines (no more than 1 drink per day for women and 2 per day for men^6^) included metabolic measures that promote inflammation, but not those that promote cardiometabolic health. For example, for low alcohol consumers we observed lower concentrations of A1, numerous subclasses of HDL metabolites, and DHA, and greater concentrations of glycoprotein acetyls and subclasses of large LDL. This finding replicates our prior study of metabolic profiles and cancer preventive behaviors^13^ and supports prior research that, for some cancers, any amount of alcohol can increase risk^27^. Conversely, moderate alcohol consumption has been associated with lower risk of atherosclerosis^28,29^. The underlying mechanism is hypothesized to be increased transport rate of ApoA1 and ApoA11 leading to raised HDL cholesterol^30^. In our study, ApoA1, and HDL subclasses were lower in participants meeting guidelines for cancer prevention which emphasis little to no alcohol compared to those who consumed more than one drink per day for women or two drinks per day for men. Our findings also align with a prior metabolic profiling study of alcohol consumption in nearly 10,000 adults^31^ that similarly reported metabolic profiles that reflected a ‘double-edged’ effect of alcohol on cardiometabolic risk factors including increased HDL subclass concentrations and LDL subclasses.

Few metabolic measures were associated with moderate to vigorous physical activity. Our finding aligns with prior studies of habitual physical activity which reported just 3% and 6% of metabolites were associated with physical activity measured by accelerometry^32^ and self-report^33^. The shift toward greater HDL and acetate and choline metabolites among participants who met physical activity guidelines in our study is consistent with an earlier study which found higher metabolites from a number of HDL subclasses and acetate in more active subjects versus less active subjects^34^. Studies of pre and post exercise also report increases in acetate metabolites^35^ and choline metabolites^36^. Choline is involved in several biochemical pathways, including mechanisms related to DNA methylation, neurotransmitters, and phosphorylation to phosphatidylcholine and sphingomyelin, which are important membrane phospholipids^37^. Altered choline metabolism has been implicated in the development of several cancers including prostate^38^, breast^39^ and colorectal cancer^40^. Together, our findings support a beneficial role of physical activity on metabolic health and possibly, processes related to cancer development.

Strengths of this study include the relatively large sample size, of well-characterized participants from the BCGP, objectively measured BMI which is less prone to bias than self-report, and assessment of metabolic measures in relation to multiple lifestyle behaviors that are outlined in cancer preventive guidelines. Findings however may have limited generalizability due to the study population which was more highly educated and had higher income compared to the general British Columbia population, and was predominately white. Former or current smoking was an exclusion criteria since smoking is the main preventable cause of cancer for some cancers and is correlated with lifestyle behaviors. Confining the population to never smokers avoids residual confounding by smoking but may limit the generalizability of findings, since smokers tend to adopt other correlated behaviors like alcohol consumption^41^. Associations between behaviors and metabolic measures were examined cross-sectionally, as serial biospecimens are not available in the BCGP cohort. The sample size and average length of follow-up of ~ 5 years also limited the ability to examine metabolic profiles, lifestyle behaviors and risk of cancer directly due to the long latency of cancer. The NMR platform used for metabolic assessment was lipids focussed as it was originally developed to study metabolic perturbations of cardiovascular disease^42^. It is possible behavior-metabolite associations in our study are more limited than in studies which have used platforms that capture metabolites from broader biological pathways and xenobiotics^19,32^. However, the breadth of lipid related metabolic measures in our study, also enabled identification of novel associations. The metabolomic assessment focussed on specific metabolite measures of known identity. It is possible that analyses of unknown metabolic measures would show different patterns, although the biological meaning of such patterns would be difficult to ascertain.

In summary, the findings replicated and identified new metabolic measures associated with adherence to cancer preventive recommendations pertaining to BMI, diet, physical activity and alcohol consumption in a cohort of Canadian men and women. A large number of metabolic associations were identified, including associations distinct to a given behavior. This suggest that lifestyle behaviours may affect cancer related pathways in different ways, and thus a broader preventive approach targeting multiple behaviours versus singular approaches (e.g. diet alone) may be more impactful. Of the behaviors assessed, the greatest metabolic shifts were observed for BMI, fruits and vegetables and alcohol consumption, suggesting these may be particularly attractive targets. The diverse pathways represented by these metabolic measures provide additional insight into mechanisms linking lifestyle behavior and cancer development, confirming a role of role of obesity-related insulin resistance, and suggesting cholesterol transport pathways as a potentially promising line of inquiry. However, prospective studies of lifestyle behaviors and incident cancer are needed to confirm findings.

## Methods

### Population

The BCGP is part of the national Canadian Partnership for Tomorrow’s Health (CanPath), a prospective, longitudinal cohort study^43^. The BCGP began in 2009 with the goal of learning more about how environment, lifestyle and genes contribute to cancer and other chronic disease. By 2015, 29,767 participants aged 30 to 74 years had enrolled^14^. Participants completed questionnaires on demographics, health history and lifestyle behaviors and provided biospecimens. A proportion (56%) underwent in-person physical measurements. For this study, the same inclusion criterion were applied as our previous study^13^ to facilitate pooling of data. A random sample of 1200 participants were selected that were 50% men, reported no current or former smoking at baseline, had no personal history of cancer, were not pregnant, attended an in-person assessment and provided a blood sample. This study was approved by the Institutional Review Board at the University of British Columbia (REB number: H17-00004). All research was performed in accordance with relevant guidelines/regulations. All participants provided informed consent.

### Lifestyle variables

Participants reported average daily servings of fresh, frozen, canned or cooked fruits and vegetables. They also reported the number, amount and type of alcoholic drinks consumed in a typical week. One drink was defined as 12 oz of beer, 5 oz of wine or 1.5 oz of 80-proof liquor/spirits. Weight was measured with bioelectrical impedance (Tanita BC-418, Tanita Corporation of America Inc.). Time spent physically active and sedentary in the prior week was assessed by the short-form International Physical Activity Questionnaire (IPAQ-SF). The IPAQ-SF is a validated, widely used questionnaire that captures different domains of physical activity at work, at home, active transportation and leisure time, time spent sitting and the physical effort of physical activity^18^.

### Cancer prevention behaviors

We focused on the main cancer prevention guidelines pertaining to body weight, diet, physical activity and alcohol intake^6^ that were assessed in the BCGP. This included body mass index (BMI), fruit and vegetable consumption (servings/day), moderate to vigorous physical activity, and alcohol consumption (drinks/week). Behaviors were considered as continuous measures, and using scores (0 and 1) for adherence to a given component, as per prior approaches that have operationalized preventive guidelines^9,13^. Preventive behavior was defined as BMI < 25 kg/m^2^, 5 or more servings of fruits and vegetables per day, no more than 2 drinks/day for men or 1 drink/day for women and at least 30 min of moderate or vigorous physical activity on 5 or more days of the week.

### Blood collection

Participants had blood drawn at a community medical laboratory using standard blood collection techniques^22^. Participants were not required to fast. Samples were stored at 4 °C before shipment to the processing laboratory the following day. Samples were processed according to BCGP’s Standard Operating Procedure^24^. EDTA vacutainer tubes were centrifuged for 10 min at 1300*g*/4 °C/with brake on to separate out the plasma fraction. Plasma was transferred to a cryovial and stored at − 80 °C between 19 to 24 h after laboratory blood collection. All samples were noted to be free of hemolysis and lipidemia.

### Metabolic assessment

Metabolic assessment on all samples was carried out by Nightingale Health Ltd. (Vantaa, Finland). The nuclear magnetic resonance (NMR) platform has been applied in a number of epidemiologic studies^12,25,26^ and methodological details have been previously published^27^. The platform captured lipoproteins, fatty acids and fatty acid composition, ketone bodies, fluid balance related  metabolites, an inflammation related metabolite, amino acids, glycolysis and gluconeogenesis-related metabolites. The same NMR setup and software library was used for metabolic quantification for the pilot and larger study. Of the 225 metabolic measures analyzed, glucose values were lower while lactate values were higher than those commonly observed in samples processed by Nightingale Health Ltd. The levels were indicative of glycolysis, with prolonged processing time at the mobile collection centers, rather than shipment conditions (verified by shipment temperature logs). Both metabolic measures were subsequently excluded from analyses along with five metabolic measures (concentration of very large, very low density lipoprotein (VLDL) phospholipid, concentration of extremely large VLDL cholesterol, concentration of extremely large VLDL cholesterol ester, concentration of extremely large VLDL free cholesterol, and concentration of extremely large VLDL triglycerides) that were below the limit of detection for > 20% of the population, leaving a total of 218 metabolic measures for analysis. As variation in levels of lactate and glucose were modest it is unlikely to have a measurable effect on other metabolites. Overall, samples were deemed to be high quality as metabolite distributions matched those observed in other cohort studies, there were no indications of irregularities or signs of degradation. The coefficient of variation determined from duplicate samples (5% of sample) was < 5% for the vast majority of metabolic measures.

### Statistical analysis

Statistical analyses were conducted using R version 4.0.3^44^ and Stata version 14.0^45^. Metabolic measures were log-transformed and centered to a mean of 0 and standard deviation of 1. When the concentration of a metabolic measure was below the limit of detection, values were set to half of the minimum value of that metabolic measure in the total population^13^. Missing covariate (education, n = 7) and predictor data (fruit and vegetables, physical activity and alcohol consumption, n = 154) were multiple imputed using the *Multivariate Imputations by Chained Equation (MICE)* package in R^46^. Age, sex, self-reported body mass index (BMI), self-perceived health, and diet quality were included as auxiliary variables in the multiple imputation^47^. Convergence was achieved with 30 iterations and 40 imputations.

Linear regression was used to evaluate associations between quantified metabolic measures modelled as the outcome and health behaviors modeled as the exposure. Health behaviors were categorized and dummy coded (0 or 1) according to cancer prevention guidelines. The reference group was those not meeting recommendations, and therefore the regression coefficient refers to the change in mean standardized value of a metabolic measure between those with a healthy behavior and those without. Associations between behaviors as continuous variables and concentrations of metabolic measures were also examined using linear regression (Supplemental Tables 1–4) to permit comparison with previous studies which have used this approach^10,42^. All models were adjusted for age, sex and a variable to indicate whether samples were part of the pilot or the larger sample. Beta coefficients, SE and p-values were reported. To account for multiple testing, false discovery rates were computed using the q value for each individual exposure^28^. Statistical significance was set at p < 0.05 and q < 0.10. Linear regression models were also mutually adjusted for cancer preventive behaviors to determine if associations were independent. Sensitivity analyses were performed with further adjustment for demographic and health variables (education, ethnicity, income, prevalent diabetes and prevalent heart disease).

Pearson pairwise correlations between all metabolic measures significantly associated with alcohol consumption, BMI, and fruits and vegetables were determined to evaluate the potential independence of metabolic associations. Physical activity related metabolites were not included due to the small identified associations. Pairwise correlations for the 25 top-ranked metabolic measures for each of alcohol, BMI, and fruits and vegetables were additionally evaluated after conditioning on other top-ranked metabolic measures^11^. Conditional correlation networks were mapped using a Gaussian Graphical Model (GGM) approach using the Cytoscape app within Metscape^48^. The GGM is visualized as metabolic measures (nodes) connected by edges that represent conditional correlations. For the sake of clarity, the network was constructed using only the edges for which the conditional correlation was in the top 10%. Metabolic measures were grouped into apolipoproteins, cholesterol, fatty acids, glycerides and phospholipids, inflammation and lipoprotein subclasses to facilitate interpretation of subnetworks.

## Supplementary Information

Supplementary Information.

## Data Availability

The datasets generated during and/or analyzed during the current study are available from the corresponding author on reasonable request and with approval from the BC Generations Project.
