# Supplementary material for: Metabolomics and cancer preventive behaviors in the BC Generations Project
Source: Sci Rep. 2021 Jun 8;11:12094. doi: 10.1038/s41598-021-91753-8 (PMC8187402; doi:10.1038/s41598-021-91753-8)
Supplement: Supplementary file 1 — Supplementary Information. [file 41598_2021_91753_MOESM1_ESM.docx]

**Metabolomics and cancer preventive behaviors in the BC Generations Project**

J Qi, JJ Spinelli, TJB Dummer, P Bhatti, MC Playdon, J Olin Levitt, B Hauner, SC Moore, RA Murphy

**Supplementary Table 1.** Metabolite measures associated with BMI in the BCGP study sample (N=1,319)

|  | ß (SE) | p-value | q-value |
| --- | --- | --- | --- |
| **Composite lipid measures** |  |  |  |
| Total serum cholesterol | -0.04 (0.03) | 0.18 | 0.02 |
| Esterified cholesterol | -0.05 (0.03) | 0.08 | 0.01 |
| Free cholesterol | -0.03 (0.03) | 0.26 | 0.03 |
| Remnant cholesterol | 0.13 (0.03) | 1.77 X 10^-6 | 2.51 X 10^-7 |
| VLDL cholesterol | 0.20 (0.03) | 1.55 X 10^-14 | <1.0 X 10^-11 |
| LDL cholesterol | 0.09 (0.03) | 0.001 | 7.55 X 10^-5 |
| HDL cholesterol | -0.36 (0.02) | <1.0 X10^-20 | <1.0 X 10^-11 |
| HDL2 cholesterol | -0.31 (0.02) | <1.0 X10^-20 | <1.0 X 10^-11 |
| HDL3 cholesterol | -0.32 (0.02) | <1.0 X10^-20 | <1.0 X 10^-11 |
| Total triacylglycerol | 0.32 (0.03) | <1.0 X10^-20 | <1.0 X 10^-11 |
| Total triacylglycerol in VLDL | 0.33 (0.02) | <1.0 X10^-20 | <1.0 X 10^-11 |
| Total triacylglycerol in LDL | 0.20 (0.03) | 5.22 X 10^-14 | <1.0 X 10^-11 |
| Total triacylglycerol in HDL | 0.09 (0.03) | 0.001 | 0.0001 |
| **Total lipids in lipoproteins** |  |  |  |
| Extremely large VLDL | 0.33 (0.03) | <1.0 X10^-20 | <1.0 X 10^-11 |
| Very large VLDL | 0.29 (0.03) | <1.0 X10^-20 | <1.0 X 10^-11 |
| Large VLDL | 0.32 (0.03) | <1.0 X10^-20 | <1.0 X 10^-11 |
| Medium VLDL | 0.20 (0.03) | 1.26 X 10^-13 | <1.0 X 10^-11 |
| Small VLDL | 0.21 (0.03) | 4.44 X 10^-16 | <1.0 X 10^-11 |
| Very small VLDL | 0.05 (0.03) | 0.07 | 0.01 |
| IDL | -0.05 (0.03) | 0.06 | 0.01 |
| Large LDL | 0.06 (0.03) | 0.03 | 0.003 |
| Medium LDL | 0.15 (0.03) | 1.13 X 10^-8 | 1.8 X 10^-9 |
| Small LDL | 0.11 (0.03) | 1.17 X 10^-5 | 1.60 X 10^-6 |
| Very large HDL | -0.38 (0.02) | <1.0 X10^-20 | <1.0 X 10^-11 |
| Large HDL | -0.40 (0.02) | <1.0 X10^-20 | <1.0 X 10^-11 |
| Medium HDL | -0.22 (0.02) | <1.0 X10^-20 | <1.0 X 10^-11 |
| Small HDL | 0.12 (0.03) | 4.70 X 10^-6 | 6.58 X 10^-7 |
| **Phospholipids in lipoproteins** |  |  |  |
| Extremely large VLDL | 0.33 (0.03) | <1.0 X10^-20 | <1.0 X 10^-11 |
| Very large VLDL | 0.25 (0.03) | <1.0 X10^-20 | <1.0 X 10^-11 |
| Large VLDL | 0.30 (0.03) | <1.0 X10^-20 | <1.0 X 10^-11 |
| Medium VLDL | 0.18 (0.03) | 4.86 X 10^-11 | <1.0 X 10^-11 |
| Small VLDL | 0.16 (0.03) | 3.32 X 10^-9 | 5.0 X 10^-10 |
| Very small VLDL | 0.05 (0.03) | 0.07 | 0.01 |
| IDL | -0.05 (0.03) | 0.04 | 0.01 |
| Large LDL | 0.03 (0.03) | 0.33 | 0.03 |
| Medium LDL | 0.12 (0.03) | 3.07 X 10^-6 | 4.32 X 10^-7 |
| Small LDL | 0.07 (0.03) | 0.01 | 0.001 |
| Very large HDL | -0.36 (0.02) | <1.0 X10^-20 | <1.0 X 10^-11 |
| Large HDL | -0.38 (0.02) | <1.0 X10^-20 | <1.0 X 10^-11 |
| Medium HDL | -0.15 (0.02) | 9.50 X 10^-11 | <1.0 X 10^-11 |
| Small HDL | 0.14 (0.03) | 2.92 X 10^-8 | 4.50 X 10^-9 |
| **Cholesterol esters in lipoproteins** |  |  |  |
| Extremely large VLDL | 0.32 (0.03) | <1.0 X10^-20 | <1.0 X 10^-11 |
| Very large VLDL | 0.26 (0.03) | <1.0 X10^-20 | <1.0 X 10^-11 |
| Large VLDL | 0.26 (0.03) | <1.0 X10^-20 | <1.0 X 10^-11 |
| Medium VLDL | -0.02 (0.03) | 0.50 | 0.05 |
| Small VLDL | 0.16 (0.03) | 4.98 X 10^-10 | 1.0 X 10^-10 |
| Very small VLDL | -0.06 (0.03) | 0.02 | 0.002 |
| IDL | -0.06 (0.03) | 0.02 | 0.002 |
| Large LDL | 0.09 (0.03) | 0.001 | 0.0001 |
| Medium LDL | 0.19 (0.03) | 1.49 X 10^-13 | <1.0 X 10^-11 |
| Small LDL | 0.15 (0.03) | 1.13 X 10^-8 | 1.80 X 10^-9 |
| Very large HDL | -0.36 (0.02) | <1.0 X10^-20 | <1.0 X 10^-11 |
| Large HDL | -0.40 (0.02) | <1.0 X10^-20 | <1.0 X 10^-11 |
| Medium HDL | -0.30 (0.02) | <1.0 X10^-20 | <1.0 X 10^-11 |
| Small HDL | 0.01 (0.03) | 0.74 | 0.07 |
| **Free cholesterol in lipoproteins** |  |  |  |
| Extremely large VLDL | 0.32 (0.03) | <1.0 X10^-20 | <1.0 X 10^-11 |
| Very large VLDL | 0.28 (0.03) | <1.0 X10^-20 | <1.0 X 10^-11 |
| Large VLDL | 0.29 (0.03) | <1.0 X10^-20 | <1.0 X 10^-11 |
| Medium VLDL | 0.18 (0.03) | 6.88 X 10^-12 | <1.0 X 10^-11 |
| Small VLDL | 0.16 (0.03) | 8.87 X 10^-10 | 2.0 X 10^-10 |
| Very small VLDL | 0.05 (0.03) | 0.04 | 0.01 |
| IDL | -0.10 (0.03) | 2.67 X 10^-4 | 3.49 X 10^-5 |
| Large LDL | -0.04 (0.03) | 0.19 | 0.02 |
| Medium LDL | 0.05 (0.03) | 0.05 | 0.006 |
| Small LDL | 0.05 (0.03) | 0.04 | 0.05 |
| Very large HDL | -0.36 (0.02) | <1.0 X10^-20 | <1.0 X 10^-11 |
| Large HDL | -0.39 (0.02) | <1.0 X10^-20 | <1.0 X 10^-11 |
| Medium HDL | -0.26 (0.02) | <1.0 X10^-20 | <1.0 X 10^-11 |
| Small HDL | -0.05 (0.02) | 0.06 | 0.01 |
| **Particles in lipoproteins** |  |  |  |
| Extremely large VLDL | 0.34 (0.03) | <1.0 X10^-20 | <1.0 X 10^-11 |
| Very large VLDL | 0.30 (0.03) | <1.0 X10^-20 | <1.0 X 10^-11 |
| Large VLDL | 0.32 (0.03) | <1.0 X10^-20 | <1.0 X 10^-11 |
| Medium VLDL | 0.21 (0.03) | 1.78 X 10^-15 | <1.0 X 10^-11 |
| Small VLDL | 0.23 (0.03) | <10^-20 | <1.0 X 10^-11 |
| Very small VLDL | 0.05 (0.03) | 0.05 | 0.01 |
| IDL | -0.04 (0.03) | 0.11 | 0.01 |
| Large LDL | 0.07 (0.03) | 0.01 | 0.001 |
| Medium LDL | 0.16 (0.03) | 1.51 X 10^-9 | 2.0 X 10^-10 |
| Small LDL | 0.08 (0.02) | 5.19 X 10^-7 | 7.52 X 10^-8 |
| Very large HDL | -0.37 (0.02) | <1.0 X10^-20 | <1.0 X 10^-11 |
| Large HDL | -0.42 (0.02) | <1.0 X10^-20 | <1.0 X 10^-11 |
| Medium HDL | -0.27 (0.02) | <1.0 X10^-20 | <1.0 X 10^-11 |
| Small HDL | 0.06 (0.03) | 0.02 | 0.002 |
| **Total cholesterol in lipoproteins** |  |  |  |
| Extremely large VLDL | 0.32 (0.03) | <1.0 X10^-20 | <1.0 X 10^-11 |
| Very large VLDL | 0.28 (0.03) | <1.0 X10^-20 | <1.0 X 10^-11 |
| Large VLDL | 0.27 (0.03) | <1.0 X10^-20 | <1.0 X 10^-11 |
| Medium VLDL | 0.06 (0.03) | 0.03 | 0.003 |
| Small VLDL | 0.17 (0.03) | 3.07 X 10^-10 | 1.0 X 10^-10 |
| Very small VLDL | -0.03 (0.03) | 0.21 | 0.02 |
| IDL | -0.03 (0.01) | 0.01 | 0.001 |
| Large LDL | 0.06 (0.03) | 0.03 | 0.003 |
| Medium LDL | 0.15 (0.03) | 6.32 X 10^-9 | 1.0 X 10^-9 |
| Small LDL | 0.11 (0.03) | 2.58 X 10^-5 | 3.48 X 10^-6 |
| Very large HDL | -0.37 (0.02) | <1.0 X10^-20 | <1.0 X 10^-11 |
| Large HDL | -0.41 (0.02) | <1.0 X10^-20 | <1.0 X 10^-11 |
| Medium HDL | -0.30 (0.02) | <1.0 X10^-20 | <1.0 X 10^-11 |
| Small HDL | 0.00 (0.03) | 0.93 | 0.09 |
| **Apolipoproteins** |  |  |  |
| Apolipoprotein A1 | -0.26 (0.02) | <1.0 X10^-20 | <1.0 X 10^-11 |
| Apolipoprotein B | 0.15 (0.03) | 4.39 X 10^-9 | 7.0 X 10^-10 |
| Apo B/Apo A1 | 0.27 (0.02) | <1.0 X10^-20 | <1.0 X 10^-11 |
| **Triacylglycerol in lipoproteins** |  |  |  |
| Extremely large VLDL | 0.33 (0.03) | <1.0 X10^-20 | <1.0 X 10^-11 |
| Very large VLDL | 0.23 (0.03) | <1.0 X10^-20 | <1.0 X 10^-11 |
| Large VLDL | 0.32 (0.03) | <1.0 X10^-20 | <1.0 X 10^-11 |
| Medium VLDL | 0.28 (0.03) | <10^-20 | <1.0 X 10^-11 |
| Small VLDL | 0.26 (0.03) | <1.0 X10^-20 | <1.0 X 10^-11 |
| Very small VLDL | 0.25 (0.03) | <1.0 X10^-20 | <1.0 X 10^-11 |
| IDL | 0.14 (0.03) | 5.59 X 10^-8 | 8.30 X 10^-9 |
| Large LDL | 0.16 (0.03) | 1.38 X 10^-9 | 2.0 X 10^-10 |
| Medium LDL | 0.21 (0.03) | 4.89 X 10^-15 | <1.0 X 10^-11 |
| Small LDL | 0.25 (0.03) | <1.0 X10^-20 | <1.0 X 10^-11 |
| Very large HDL | -0.15 (0.03) | 6.71 X 10^-9 | 1.10 X 10^-9 |
| Large HDL | -0.28 (0.02) | <1.0 X10^-20 | <1.0 X 10^-11 |
| Medium HDL | 0.16 (0.03) | 1.22 X 10^-9 | 2.0 X 10^-10 |
| Small HDL | 0.30 (0.03) | <1.0 X10^-20 | <1.0 X 10^-11 |
| **Lipoprotein particle size** |  |  |  |
| VLDL particle size | 0.30 (0.02) | <1.0 X10^-20 | <1.0 X 10^-11 |
| LDL particle size | -0.07 (0.01) | <1.0 X10^-20 | <1.0 X 10^-11 |
| HDL particle size | -0.30 (0.02) | <1.0 X10^-20 | <1.0 X 10^-11 |
| **Total cholesterol in lipoproteins (%)** |  |  |  |
| Very large VLDL | 0.04 (0.03) | 0.17 | 0.02 |
| Large VLDL | 0.07 (0.03) | 0.01 | 0.001 |
| Medium VLDL | -0.22 (0.03) | <1.0 X10^-20 | <1.0 X 10^-11 |
| Small VLDL | -0.02 (0.03) | 0.39 | 0.04 |
| Very small VLDL | -0.22 (0.02) | <1.0 X10^-20 | <1.0 X 10^-11 |
| IDL | -0.13 (0.02) | 8.39 X 10^-9 | 1.30 X 10^-9 |
| Large LDL | 0.04 (0.02) | 0.08 | 0.01 |
| Medium LDL | 0.09 (0.02) | 5.98 X 10^-6 | 8.30 X 10^-7 |
| Small LDL | -0.01 (0.02) | 0.71 | 0.07 |
| Very large HDL | 0.13 (0.03) | 1.12 X 10^-6 | 1.60 X 10^-7 |
| Large HDL | -0.28 (0.03) | <1.0 X10^-20 | <1.0 X 10^-11 |
| Medium HDL | -0.35 (0.02) | <1.0 X10^-20 | <1.0 X 10^-11 |
| Small HDL | -0.22 (0.02) | <1.0 X10^-20 | <1.0 X 10^-11 |
| **Phospholipids in lipoproteins (%)** |  |  |  |
| Very large VLDL | 0.07 (0.03) | 0.01 | 0.001 |
| Large VLDL | 0.01 (0.03) | 0.78 | 0.07 |
| Medium VLDL | -0.12 (0.03) | 1.04 X 10^-5 | 1.43 X 10^-6 |
| Small VLDL | -0.14 (0.03) | 1.14 X 10^-7 | 1.67 X 10^-8 |
| Very small VLDL | 0.00 (0.02) | 0.96 | 0.09 |
| IDL | -0.01 (0.02) | 0.65 | 0.06 |
| Large LDL | -0.18 (0.02) | 1.73 X 10^-14 | <1.0 X 10^-11 |
| Medium LDL | -0.19 (0.02) | <1.0 X10^-20 | <1.0 X 10^-11 |
| Small LDL | -0.21 (0.02) | <1.0 X10^-20 | <1.0 X 10^-11 |
| Very large HDL | -0.25 (0.03) | <1.0 X10^-20 | <1.0 X 10^-11 |
| Large HDL | 0.02 (0.03) | 0.41 | 0.04 |
| Medium HDL | 0.36 (0.02) | <1.0 X10^-20 | <1.0 X 10^-11 |
| Small HDL | 0.11 (0.02) | 5.37 X 10^-7 | 7.73 X 10^-8 |
| **Cholesterol esters in lipoproteins (%)** | |  |  |
| Very large VLDL | -0.03 (0.03) | 0.24 | 0.02 |
| Large VLDL | 0.00 (0.03) | 0.96 | 0.09 |
| Medium VLDL | -0.23 (0.03) | <1.0 X10^-20 | <1.0 X 10^-11 |
| Small VLDL | 0.05 (0.03) | 0.07 | 0.01 |
| Very small VLDL | -0.24 (0.02) | <1.0 X10^-20 | <1.0 X 10^-11 |
| IDL | -0.09 (0.02) | 1.14 X 10^-4 | 1.50 X 10^-5 |
| Large LDL | 0.19 (0.02) | <1.0 X10^-20 | <1.0 X 10^-11 |
| Medium LDL | 0.25 (0.02) | <1.0 X10^-20 | <1.0 X 10^-11 |
| Small LDL | 0.15 (0.02) | 1.46 X 10 ^-12 | <1.0 X 10^-11 |
| Very large HDL | -0.05 (0.03) | 0.05 | 0.01 |
| Large HDL | -0.27 (0.03) | <1.0 X10^-20 | <1.0 X 10^-11 |
| Medium HDL | -0.35 (0.02) | <1.0 X10^-20 | <1.0 X 10^-11 |
| Small HDL | -0.18 (0.02) | 4.84 X 10^-14 | <1.0 X 10^-11 |
| **Free cholesterol in lipoproteins (%)** |  |  |  |
| Very large VLDL | 0.19 (0.03) | 1.74 X 10^-12 | <1.0 X 10^-11 |
| Large VLDL | 0.17 (0.03) | 1.86 X 10^-10 | <1.0 X 10^-11 |
| Medium VLDL | 0.02 (0.03) | 0.57 | 0.06 |
| Small VLDL | -0.10 (0.03) | 8.63 X 10^-5 | 1.15 X 10^-5 |
| Very small VLDL | 0.06 (0.02) | 0.02 | 0.002 |
| IDL | -0.21 (0.02) | <10^-20 | <1.0 X 10^-11 |
| Large LDL | -0.31 (0.02) | <1.0 X10^-20 | <1.0 X 10^-11 |
| Medium LDL | -0.29 (0.02) | <1.0 X10^-20 | <1.0 X 10^-11 |
| Small LDL | -0.16 (0.03) | 1.46 X 10^-10 | <1.0 X 10^-11 |
| Very large HDL | 0.28 (0.03) | <1.0 X10^-20 | <1.0 X 10^-11 |
| Large HDL | -0.15 (0.03) | 1.41 X 10^-8 | 2.20 X 10^-9 |
| Medium HDL | -0.30 (0.02) | <1.0 X10^-20 | <1.0 X 10^-11 |
| Small HDL | -0.31 (0.02) | <1.0 X10^-20 | <1.0 X 10^-11 |
| **Triacylglycerol in lipoproteins (%)** |  |  |  |
| Very large VLDL | 0.15 (0.03) | 3.64 X 10^-8 | 5.60 X 10^-9 |
| Large VLDL | 0.14 (0.03) | 4.90 X 10^-8 | 7.40 X 10^-9 |
| Medium VLDL | 0.23 (0.03) | <1.0 X10^-20 | <1.0 X 10^-11 |
| Small VLDL | 0.18 (0.03) | 2.12 X 10^-12 | <1.0 X 10^-11 |
| Very small VLDL | 0.26 (0.03) | <10^-20 | <1.0 X 10^-11 |
| IDL | 0.19 (0.03) | 1.17 X 10^-12 | <1.0 X 10^-11 |
| Large LDL | 0.09 (0.03) | 0.0006 | 7.53 X 10^-5 |
| Medium LDL | 0.04 (0.03) | 0.13 | 0.01 |
| Small LDL | 0.19 (0.03) | 1.15 X 10^-12 | <1.0 X 10^-11 |
| Very large HDL | 0.26 (0.03) | <1.0 X10^-20 | <1.0 X 10^-11 |
| Large HDL | 0.21 (0.03) | 1.11 X 10^-14 | <1.0 X 10^-11 |
| Medium HDL | 0.28 (0.03) | <1.0 X10^-20 | <1.0 X 10^-11 |
| Small HDL | 0.28 (0.03) | <1.0 X10^-20 | <1.0 X 10^-11 |
| **Branched-chain amino acids** |  |  |  |
| Isoleucine | 0.20 (0.03) | 2.78 X 10^-13 | <1.0 X 10^-11 |
| Leucine | 0.18 (0.03) | 1.75 X 10^-11 | <1.0 X 10^-11 |
| Valine | 0.14 (0.03) | 4.08 X 10^-8 | 6.20 X 10^-9 |
| **Aromatic amino acids** |  |  |  |
| Phenylalanine | 0.10 (0.03) | 0.0001 | 1.38 X 10^-5 |
| Tyrosine | 0.07 (0.03) | 0.01 | 0.001 |
| Histidine | -0.11 (0.03) | 1.69 X 10^-5 | 2.30 X 10^-6 |
| **Other amino acids** |  |  |  |
| Alanine | 0.06 (0.03) | 0.03 | 0.003 |
| Glutamine | -0.03 (0.02) | <1.0 X10^-20 | <1.0 X 10^-11 |
| **Ketone bodies** |  |  |  |
| Acetoacetate | 0.06 (0.03) | 0.03 | 0.003 |
| 3-hydroxybutyrate | 0.09 (0.03) | 0.001 | 9.56 X 10^-5 |
| **Miscellaneous** |  |  |  |
| Creatinine | 0.05 (0.02) | 0.02 | 0.002 |
| Albumin | -0.05 (0.02) | 0.01 | 0.001 |
| Acetate | -0.03 (0.03) | 0.29 | 0.03 |
| Citrate | -0.16 (0.03) | 1.02 X 10^-9 | 2.0 X 10^-10 |
| Glycoprotein acetyls | 0.40 (0.02) | <10^-20 | <1.0 X 10^-11 |
| **Fatty acids** |  |  |  |
| Total fatty acids | 0.18 (0.03) | 6.23 X 10^-12 | <1.0 X 10^-11 |
| Degree of unsaturation | -0.30 (0.02) | <1.0 X10^-20 | <1.0 X 10^-11 |
| DHA | 0.04 (0.03) | 0.12 | 0.01 |
| Linoleic acid | 0.01 (0.03) | 0.79 | 0.07 |
| Saturated fatty acids | 0.20 (0.03) | 1.91 X 10^-14 | <1.0 X 10^-11 |
| n-3 fatty acids | 0.07 (0.03) | 0.01 | 0.0006 |
| n-6 fatty acids | 0.04 (0.03) | 0.17 | 0.02 |
| Monounsaturated fatty acids | 0.27 (0.03) | <1.0 X10^-20 | <1.0 X 10^-11 |
| Polyunsaturated fatty acids | 0.04 (0.03) | 0.09 | 0.01 |
| **Fatty acid ratios** |  |  |  |
| Saturated fatty acids (%) | 0.10 (0.03) | 3.83 X 10^-5 | 5.14 X 10^-6 |
| Monounsaturated fatty acids (%) | 0.35 (0.02) | <1.0 X10^-20 | <1.0 X 10^-11 |
| Polyunsaturated fatty acids (%) | -0.38 (0.02) | <1.0 X10^-20 | <1.0 X 10^-11 |
| n-6 fatty acids (%) | -0.37 (0.02) | <1.0 X10^-20 | <1.0 X 10^-11 |
| Linoleic acid (%) | -0.36 (0.02) | <1.0 X10^-20 | <1.0 X 10^-11 |
| n-3 fatty acids (%) | -0.15 (0.03) | 5.34 X 10^-8 | 8.0 X 10^-9 |
| DHA (%) | -0.14 (0.03) | 1.24 X 10^-7 | 1.81 X 10^-8 |
| **Phospholipids** |  |  |  |
| Sphingomyelins | -0.03 (0.03) | 0.20 | 0.02 |
| Total cholines | -0.03 (0.03) | 0.19 | 0.02 |
| Phosphatidylcholines | -0.03 (0.03) | 0.31 | 0.03 |
| Total phosphoglycerides | 0.00 (0.03) | 0.91 | 0.09 |
| TG/PG | 0.35 (0.02) | <1.0 X10^-20 | <1.0 X 10^-11 |
| Significance determined at p<0.05 and q<0.10. Linear regression, comparing metabolites (outcome) with BMI on a continuous scale (exposure), models adjusted for age, sex and study (pilot or larger sample) | | | |

**Supplementary Table 2.** Metabolite measures associated with fruit and vegetable consumption in the BCGP study sample (N=1,319)

|  | ß (SE) | p-value | q-value |
| --- | --- | --- | --- |
| **Composite lipid measures** |  |  |  |
| Total serum cholesterol | -0.04 (0.03) | 0.11 | 0.08 |
| Esterified cholesterol | -0.04 (0.03) | 0.12 | 0.08 |
| Free cholesterol | -0.04 (0.03) | 0.17 | 0.10 |
| Remnant cholesterol | -0.05 (0.03) | 0.08 | 0.07 |
| VLDL cholesterol | -0.04 (0.03) | 0.11 | 0.08 |
| LDL cholesterol | -0.06 (0.03) | 0.03 | 0.05 |
| HDL cholesterol | 0.02 (0.03) | 0.38 | 0.18 |
| HDL2 cholesterol | 0.02 (0.03) | 0.45 | 0.20 |
| HDL3 cholesterol | 0.03 (0.03) | 0.23 | 0.12 |
| Total triacylglycerol | -0.08 (0.03) | 0.01 | 0.03 |
| Total triacylglycerol in VLDL | -0.07 (0.03) | 0.01 | 0.03 |
| Total triacylglycerol in LDL | -0.07 (0.03) | 0.02 | 0.04 |
| Total triacylglycerol in HDL | -0.03 (0.03) | 0.24 | 0.13 |
| **Total lipids in lipoproteins** |  |  |  |
| Extremely large VLDL | -0.10 (0.03) | 8.91 X 10^-6 | 0.0001 |
| Very large VLDL | -0.06 (0.03) | 0.02 | 0.04 |
| Large VLDL | -0.07 (0.03) | 0.01 | 0.03 |
| Medium VLDL | -0.04 (0.03) | 0.14 | 0.10 |
| Small VLDL | -0.04 (0.03) | 0.11 | 0.08 |
| Very small VLDL | -0.03 (0.03) | 0.31 | 0.16 |
| IDL | -0.04 (0.03) | 0.17 | 0.10 |
| Large LDL | -0.06 (0.03) | 0.04 | 0.05 |
| Medium LDL | -0.07 (0.03) | 0.01 | 0.03 |
| Small LDL | -0.06 (0.03) | 0.02 | 0.04 |
| Very large HDL | 0.05 (0.03) | 0.04 | 0.05 |
| Large HDL | 0.05 (0.03) | 0.06 | 0.06 |
| Medium HDL | -0.01 (0.02) | 0.74 | 0.29 |
| Small HDL | -0.10 (0.03) | 0.22 | 0.12 |
| **Phospholipids in lipoproteins** |  |  |  |
| Extremely large VLDL | -0.10 (0.03) | 4.61 X 10^-6 | 0.0001 |
| Very large VLDL | -0.05 (0.03) | 0.05 | 0.05 |
| Large VLDL | -0.06 (0.03) | 0.02 | 0.04 |
| Medium VLDL | -0.04 (0.03) | 0.14 | 0.10 |
| Small VLDL | -0.04 (0.03) | 0.13 | 0.09 |
| Very small VLDL | -0.03 (0.03) | 0.33 | 0.16 |
| IDL | -0.04 (0.03) | 0.20 | 0.12 |
| Large LDL | -0.05 (0.03) | 0.05 | 0.05 |
| Medium LDL | -0.07 (0.03) | 0.01 | 0.03 |
| Small LDL | -0.06 (0.03) | 0.04 | 0.05 |
| Very large HDL | 0.04 (0.03) | 0.10 | 0.08 |
| Large HDL | 0.05 (0.03) | 0.09 | 0.07 |
| Medium HDL | -0.02 (0.02) | 0.38 | 0.18 |
| Small HDL | -0.10 (0.03) | 0.15 | 0.10 |
| **Cholesterol esters in lipoproteins** |  |  |  |
| Extremely large VLDL | -0.09 (0.03) | 1.53 X 10^-5 | 0.0001 |
| Very large VLDL | -0.05 (0.03) | 0.09 | 0.07 |
| Large VLDL | -0.04 (0.03) | 0.13 | 0.09 |
| Medium VLDL | 0.00 (0.03) | 0.87 | 0.33 |
| Small VLDL | -0.03 (0.03) | 0.20 | 0.12 |
| Very small VLDL | -0.01 (0.03) | 0.71 | 0.29 |
| IDL | -0.04 (0.03) | 0.17 | 0.10 |
| Large LDL | -0.06 (0.03) | 0.03 | 0.05 |
| Medium LDL | -0.07 (0.03) | 0.01 | 0.03 |
| Small LDL | -0.06 (0.03) | 0.02 | 0.04 |
| Very large HDL | 0.06 (0.03) | 0.04 | 0.05 |
| Large HDL | 0.05 (0.03) | 0.04 | 0.05 |
| Medium HDL | 0.01 (0.03) | 0.62 | 0.26 |
| Small HDL | -0.07 (0.03) | 0.01 | 0.03 |
| **Free cholesterol in lipoproteins** |  |  |  |
| Extremely large VLDL | -0.09 (0.03) | 1.39 X 10^-5 | 0.0001 |
| Very large VLDL | -0.06 (0.03) | 0.05 | 0.05 |
| Large VLDL | -0.05 (0.03) | 0.06 | 0.06 |
| Medium VLDL | -0.04 (0.03) | 0.19 | 0.11 |
| Small VLDL | -0.04 (0.03) | 0.20 | 0.12 |
| Very small VLDL | -0.02 (0.03) | 0.38 | 0.18 |
| IDL | -0.02 (0.03) | 0.38 | 0.18 |
| Large LDL | -0.03 (0.03) | 0.22 | 0.12 |
| Medium LDL | -0.05 (0.03) | 0.07 | 0.06 |
| Small LDL | -0.05 (0.03) | 0.05 | 0.05 |
| Very large HDL | 0.04 (0.03) | 0.11 | 0.08 |
| Large HDL | 0.05 (0.03) | 0.06 | 0.06 |
| Medium HDL | 0.00 (0.02) | 1.00 | 0.36 |
| Small HDL | -0.06 (0.03) | 0.02 | 0.04 |
| **Particles in lipoproteins** |  |  |  |
| Extremely large VLDL | -0.10 (0.03) | 9.46 X 10^-6 | 0.0001 |
| Very large VLDL | -0.06 (0.03) | 0.02 | 0.04 |
| Large VLDL | -0.07 (0.03) | 0.01 | 0.03 |
| Medium VLDL | -0.04 (0.03) | 0.13 | 0.09 |
| Small VLDL | -0.04 (0.03) | 0.11 | 0.08 |
| Very small VLDL | -0.03 (0.03) | 0.27 | 0.14 |
| IDL | -0.04 (0.03) | 0.14 | 0.09 |
| Large LDL | -0.06 (0.03) | 0.04 | 0.05 |
| Medium LDL | -0.07 (0.03) | 0.01 | 0.03 |
| Small LDL | -0.06 (0.03) | 0.02 | 0.04 |
| Very large HDL | 0.05 (0.03) | 0.04 | 0.05 |
| Large HDL | 0.05 (0.03) | 0.06 | 0.06 |
| Medium HDL | -0.01 (0.02) | 0.74 | 0.29 |
| Small HDL | -0.10 (0.03) | 0.22 | 0.12 |
| **Total cholesterol in lipoproteins** |  |  |  |
| Extremely large VLDL | -0.09 (0.03) | 1.44 X 10^-5 | 0.0001 |
| Very large VLDL | -0.05 (0.03) | 0.06 | 0.06 |
| Large VLDL | -0.05 (0.03) | 0.09 | 0.07 |
| Medium VLDL | -0.01 (0.03) | 0.69 | 0.28 |
| Small VLDL | -0.04 (0.03) | 0.20 | 0.12 |
| Very small VLDL | -0.01 (0.03) | 0.61 | 0.26 |
| IDL | -0.01 (0.01) | 0.21 | 0.12 |
| Large LDL | -0.05 (0.03) | 0.05 | 0.05 |
| Medium LDL | -0.06 (0.03) | 0.02 | 0.04 |
| Small LDL | -0.06 (0.03) | 0.02 | 0.04 |
| Very large HDL | 0.06 (0.03) | 0.04 | 0.05 |
| Large HDL | 0.05 (0.03) | 0.04 | 0.05 |
| Medium HDL | 0.01 (0.03) | 0.66 | 0.27 |
| Small HDL | -0.07 (0.03) | 0.01 | 0.03 |
| **Apolipoproteins** |  |  |  |
| Apolipoprotein A1 | -0.01 (0.02) | 0.81 | 0.32 |
| Apolipoprotein B | -0.06 (0.03) | 0.04 | 0.05 |
| Apo B/Apo A1 | -0.04 (0.03) | 0.09 | 0.07 |
| **Triacylglycerol in lipoproteins** |  |  |  |
| Extremely large VLDL | -0.10 (0.03) | 1.09 X 10^-5 | 0.0001 |
| Very large VLDL | -0.05 (0.03) | 0.09 | 0.07 |
| Large VLDL | -0.07 (0.03) | 0.01 | 0.03 |
| Medium VLDL | -0.05 (0.03) | 0.07 | 0.06 |
| Small VLDL | -0.04 (0.03) | 0.15 | 0.10 |
| Very small VLDL | -0.05 (0.03) | 0.09 | 0.07 |
| IDL | -0.05 (0.03) | 0.08 | 0.07 |
| Large LDL | -0.06 (0.03) | 0.03 | 0.05 |
| Medium LDL | -0.07 (0.03) | 0.02 | 0.04 |
| Small LDL | -0.07 (0.03) | 0.01 | 0.03 |
| Very large HDL | 0.02 (0.03) | 0.53 | 0.23 |
| Large HDL | 0.05 (0.03) | 0.09 | 0.07 |
| Medium HDL | -0.04 (0.03) | 0.13 | 0.09 |
| Small HDL | -0.06 (0.03) | 0.02 | 0.04 |
| **Lipoprotein particle size** |  |  |  |
| VLDL particle size | -0.06 (0.02) | 0.01 | 0.03 |
| LDL particle size | 0.01 (0.01) | 0.17 | 0.10 |
| HDL particle size | 0.05 (0.02) | 0.01 | 0.03 |
| **Total cholesterol in lipoproteins (%)** |  |  |  |
| Very large VLDL | 0.02 (0.03) | 0.46 | 0.20 |
| Large VLDL | 0.02 (0.03) | 0.52 | 0.22 |
| Medium VLDL | 0.05 (0.03) | 0.05 | 0.05 |
| Small VLDL | 0.00 (0.03) | 0.92 | 0.34 |
| Very small VLDL | 0.03 (0.03) | 0.30 | 0.16 |
| IDL | 0.00 (0.02) | 0.95 | 0.35 |
| Large LDL | -0.02 (0.03) | 0.37 | 0.18 |
| Medium LDL | -0.02 (0.02) | 0.45 | 0.20 |
| Small LDL | 0.00 (0.02) | 0.84 | 0.32 |
| Very large HDL | -0.01 (0.03) | 0.82 | 0.32 |
| Large HDL | 0.06 (0.03) | 0.05 | 0.05 |
| Medium HDL | 0.05 (0.03) | 0.05 | 0.05 |
| Small HDL | 0.04 (0.02) | 0.12 | 0.08 |
| **Phospholipids in lipoproteins (%)** |  |  |  |
| Very large VLDL | -0.01 (0.03) | 0.66 | 0.27 |
| Large VLDL | 0.00 (0.03) | 0.97 | 0.36 |
| Medium VLDL | 0.03 (0.03) | 0.33 | 0.16 |
| Small VLDL | 0.01 (0.03) | 0.74 | 0.29 |
| Very small VLDL | 0.00 (0.02) | 0.90 | 0.34 |
| IDL | 0.02 (0.02) | 0.49 | 0.21 |
| Large LDL | 0.04 (0.03) | 0.08 | 0.07 |
| Medium LDL | 0.02 (0.03) | 0.29 | 0.15 |
| Small LDL | 0.03 (0.03) | 0.21 | 0.12 |
| Very large HDL | 0.03 (0.03) | 0.34 | 0.17 |
| Large HDL | 0.00 (0.03) | 0.98 | 0.36 |
| Medium HDL | -0.06 (0.03) | 0.03 | 0.05 |
| Small HDL | -0.03 (0.03) | 0.15 | 0.10 |
| **Cholesterol esters in lipoproteins (%)** | |  |  |
| Very large VLDL | 0.04 (0.03) | 0.21 | 0.12 |
| Large VLDL | 0.03 (0.03) | 0.21 | 0.12 |
| Medium VLDL | 0.05 (0.03) | 0.05 | 0.05 |
| Small VLDL | -0.01 (0.03) | 0.68 | 0.28 |
| Very small VLDL | 0.03 (0.03) | 0.30 | 0.16 |
| IDL | -0.02 (0.02) | 0.45 | 0.20 |
| Large LDL | -0.06 (0.02) | 0.02 | 0.04 |
| Medium LDL | -0.05 (0.02) | 0.05 | 0.05 |
| Small LDL | -0.01 (0.02) | 0.56 | 0.24 |
| Very large HDL | 0.02 (0.03) | 0.40 | 0.18 |
| Large HDL | 0.05 (0.03) | 0.06 | 0.06 |
| Medium HDL | 0.05 (0.03) | 0.05 | 0.05 |
| Small HDL | 0.03 (0.03) | 0.20 | 0.12 |
| **Free cholesterol in lipoproteins (%)** |  |  |  |
| Very large VLDL | -0.03 (0.03) | 0.33 | 0.16 |
| Large VLDL | -0.01 (0.03) | 0.67 | 0.28 |
| Medium VLDL | 0.02 (0.03) | 0.47 | 0.21 |
| Small VLDL | 0.02 (0.03) | 0.57 | 0.24 |
| Very small VLDL | 0.00 (0.03) | 0.93 | 0.35 |
| IDL | 0.05 (0.02) | 0.04 | 0.05 |
| Large LDL | 0.07 (0.02) | 0.01 | 0.03 |
| Medium LDL | 0.06 (0.02) | 0.04 | 0.05 |
| Small LDL | 0.02 (0.02) | 0.43 | 0.20 |
| Very large HDL | -0.04 (0.03) | 0.12 | 0.08 |
| Large HDL | 0.03 (0.03) | 0.23 | 0.12 |
| Medium HDL | 0.02 (0.03) | 0.31 | 0.16 |
| Small HDL | 0.07 (0.03) | 0.01 | 0.03 |
| **Triacylglycerol in lipoproteins (%)** |  |  |  |
| Very large VLDL | 0.03 (0.03) | 0.35 | 0.17 |
| Large VLDL | -0.05 (0.03) | 0.10 | 0.08 |
| Medium VLDL | -0.02 (0.03) | 0.39 | 0.18 |
| Small VLDL | -0.01 (0.03) | 0.74 | 0.29 |
| Very small VLDL | -0.03 (0.03) | 0.31 | 0.16 |
| IDL | -0.01 (0.03) | 0.83 | 0.32 |
| Large LDL | 0.01 (0.03) | 0.84 | 0.32 |
| Medium LDL | 0.01 (0.03) | 0.72 | 0.29 |
| Small LDL | -0.03 (0.03) | 0.37 | 0.18 |
| Very large HDL | -0.04 (0.03) | 0.18 | 0.11 |
| Large HDL | 0.00 (0.03) | 0.87 | 0.33 |
| Medium HDL | -0.03 (0.02) | 0.21 | 0.12 |
| Small HDL | -0.03 (0.03) | 0.27 | 0.14 |
| **Branched-chain amino acids** |  |  |  |
| Isoleucine | -0.03 (0.03) | 0.28 | 0.15 |
| Leucine | -0.03 (0.03) | 0.32 | 0.16 |
| Valine | 0.00 (0.03) | 0.99 | 0.36 |
| **Aromatic amino acids** |  |  |  |
| Phenylalanine | 0.01 (0.03) | 0.83 | 0.32 |
| Tyrosine | 0.01 (0.03) | 0.81 | 0.32 |
| Histidine | 0.05 (0.03) | 0.08 | 0.07 |
| **Other amino acids** |  |  |  |
| Alanine | 0.00 (0.03) | 0.96 | 0.35 |
| Glutamine | 0.02 (0.03) | 0.50 | 0.22 |
| **Ketone bodies** |  |  |  |
| Acetoacetate | 0.04 (0.03) | 0.10 | 0.08 |
| 3-hydroxybutyrate | -0.03 (0.03) | 0.37 | 0.18 |
| **Miscellaneous** |  |  |  |
| Creatinine | -0.04 (0.02) | 0.12 | 0.08 |
| Albumin | -0.01 (0.02) | 0.81 | 0.32 |
| Acetate | 0.07 (0.03) | 0.01 | 0.03 |
| Citrate | 0.04 (0.03) | 0.11 | 0.08 |
| Glycoprotein acetyls | -0.08 (0.03) | 1.34 X 10^-5 | 0.0001 |
| **Fatty acids** |  |  |  |
| Total fatty acids | -0.07 (0.03) | 0.01 | 0.03 |
| Degree of unsaturation | 0.10 (0.03) | 1.42 X 10^-6 | 0.0001 |
| DHA | 0.02 (0.03) | 0.37 | 0.18 |
| Linoleic acid | -0.05 (0.03) | 0.06 | 0.06 |
| Saturated fatty acids | -0.08 (0.03) | 0.05 | 0.05 |
| n-3 fatty acids | 0.00 (0.03) | 0.86 | 0.33 |
| n-6 fatty acids | -0.05 (0.03) | 0.06 | 0.06 |
| Monounsaturated fatty acids | -0.08 (0.03) | 0.01 | 0.03 |
| Polyunsaturated fatty acids | -0.04 (0.03) | 0.12 | 0.08 |
| **Fatty acid ratios** |  |  |  |
| Saturated fatty acids (%) | -0.05 (0.03) | 0.04 | 0.05 |
| Monounsaturated fatty acids (%) | -0.06 (0.03) | 0.03 | 0.05 |
| Polyunsaturated fatty acids (%) | 0.08 (0.03) | 0.003 | 0.02 |
| n-6 fatty acids (%) | 0.06 (0.03) | 0.03 | 0.05 |
| Linoleic acid (%) | 0.04 (0.03) | 0.14 | 0.09 |
| n-3 fatty acids (%) | 0.11 (0.03) | 0.34 | 0.16 |
| DHA (%) | 0.11 (0.03) | 0.0002 | 0.002 |
| **Phospholipids** |  |  |  |
| Sphingomyelins | -0.05 (0.03) | 0.05 | 0.05 |
| Total cholines | -0.04 (0.03) | 0.11 | 0.08 |
| Phosphatidylcholines | -0.04 (0.03) | 0.10 | 0.08 |
| Total phosphoglycerides | -0.05 (0.03) | 0.07 | 0.06 |
| TG/PG | -0.06 (0.03) | 0.04 | 0.05 |
| Significance determined at p<0.05 and q<0.10. Linear regression, comparing metabolites (outcome) with fruit and vegetable consumption per week on a continuous scale (exposure), models adjusted for age, sex and study (pilot or larger sample). | | | |

**Supplementary Table 3.** Metabolite measures associated with alcohol consumption in the BCGP study sample (N=1,319)

|  | | | |
| --- | --- | --- | --- |
|  | ß (SE) | p-value | q-value |
| **Composite lipid measures** |  |  |  |
| Total serum cholesterol | 0.05 (0.02) | 0.02 | 0.02 |
| Esterified cholesterol | 0.06 (0.02) | 0.02 | 0.02 |
| Free cholesterol | 0.04 (0.02) | 0.08 | 0.06 |
| Remnant cholesterol | 0.00 (0.02) | 0.93 | 0.38 |
| VLDL cholesterol | -0.03 (0.02) | 0.22 | 0.14 |
| LDL cholesterol | -0.02 (0.02) | 0.40 | 0.21 |
| HDL cholesterol | 0.15 (0.02) | 5.06 X 10^-13 | <1.0 X 10^-11 |
| HDL2 cholesterol | 0.15 (0.02) | 2.64 X 10^-13 | <1.0 X 10^-11 |
| HDL3 cholesterol | 0.08 (0.02) | 3.24 X 10^-4 | 0.0007 |
| Total triacylglycerol | -0.02 (0.02) | 0.34 | 0.19 |
| Total triacylglycerol in VLDL | -0.04 (0.02) | 0.12 | 0.08 |
| Total triacylglycerol in LDL | 0.01 (0.02) | 0.71 | 0.31 |
| Total triacylglycerol in HDL | 0.09 (0.02) | 0.002 | 0.007 |
| **Total lipids in lipoproteins** |  |  |  |
| Extremely large VLDL | -0.01 (0.02) | 0.58 | 0.27 |
| Very large VLDL | -0.04 (0.02) | 0.09 | 0.07 |
| Large VLDL | -0.04 (0.02) | 0.06 | 0.05 |
| Medium VLDL | -0.03 (0.02) | 0.17 | 0.10 |
| Small VLDL | -0.06 (0.02) | 0.01 | 0.01 |
| Very small VLDL | 0.05 (0.02) | 0.03 | 0.03 |
| IDL | -0.04 (0.02) | 0.06 | 0.05 |
| Large LDL | -0.01 (0.02) | 0.75 | 0.32 |
| Medium LDL | -0.04 (0.02) | 0.11 | 0.08 |
| Small LDL | -0.01 (0.01) | 0.66 | 0.30 |
| Very large HDL | -0.01 (0.01) | 0.66 | 0.30 |
| Large HDL | 0.11 (0.02) | 0.00 | 0.00 |
| Medium HDL | 0.17 (0.02) | 0.00 | 0.00 |
| Small HDL | 0.15 (0.02) | 0.00 | 0.00 |
| **Phospholipids in lipoproteins** |  |  |  |
| Extremely large VLDL | -0.01 (0.02) | 0.66 | 0.30 |
| Very large VLDL | -0.04 (0.02) | 0.11 | 0.08 |
| Large VLDL | -0.04 (0.02) | 0.06 | 0.05 |
| Medium VLDL | -0.02 (0.02) | 0.35 | 0.19 |
| Small VLDL | -0.06 (0.02) | 0.01 | 0.01 |
| Very small VLDL | 0.05 (0.02) | 0.03 | 0.03 |
| IDL | -0.05 (0.02) | 0.04 | 0.04 |
| Large LDL | 0.00 (0.02) | 0.88 | 0.37 |
| Medium LDL | -0.03 (0.02) | 0.19 | 0.12 |
| Small LDL | -0.01 (0.02) | 0.79 | 0.34 |
| Very large HDL | 0.11 (0.02) | 1.86 X 10^-5 | 4.87 X 10^-5 |
| Large HDL | 0.11 (0.02) | 8.20 X 10^-7 | 2.87 X 10^-6 |
| Medium HDL | 0.17 (0.02) | 8.88 X 10^-16 | <1.0 X 10^-11 |
| Small HDL | 0.15 (0.02) | 9.28 X 10^-12 | 2.87 X 10^-6 |
| **Cholesterol esters in lipoproteins** |  |  |  |
| Extremely large VLDL | -0.02 (0.02) | 0.45 | 0.23 |
| Very large VLDL | -0.05 (0.03) | 0.04 | 0.04 |
| Large VLDL | -0.06 (0.02) | 0.02 | 0.02 |
| Medium VLDL | -0.02 (0.03) | 0.50 | 0.24 |
| Small VLDL | -0.05 (0.02) | 0.02 | 0.02 |
| Very small VLDL | 0.07 (0.02) | 0.0001 | 0.002 |
| IDL | 0.02 (0.01) | 0.05 | 0.05 |
| Large LDL | -0.01 (0.02) | 0.67 | 0.30 |
| Medium LDL | -0.04 (0.02) | 0.07 | 0.06 |
| Small LDL | -0.02 (0.02) | 0.42 | 0.22 |
| Very large HDL | 0.11 (0.02) | 8.11 X 10^-6 | 2.87 X 10^-6 |
| Large HDL | 0.10 (0.02) | 9.20 X 10^-6 | 2.49 X 10^-5 |
| Medium HDL | 0.16 (0.02) | 2.50 X 10^-13 | <1.0 X 10^-11 |
| Small HDL | 0.14 (0.02) | 8.62 X 10 ^-10 | 4.50 X 10^-9 |
| **Free cholesterol in lipoproteins** |  |  |  |
| Extremely large VLDL | -0.01 (0.02) | 0.59 | 0.27 |
| Very large VLDL | -0.03 (0.02) | 0.14 | 0.09 |
| Large VLDL | -0.05 (0.02) | 0.05 | 0.05 |
| Medium VLDL | -0.01 (0.02) | 0.61 | 0.28 |
| Small VLDL | -0.05 (0.02) | 0.02 | 0.02 |
| Very small VLDL | 0.07 (0.02) | 0.12 | 0.09 |
| IDL | 0.05 (0.02) | 0.05 | 0.05 |
| Large LDL | -0.01 (0.02) | 0.57 | 0.27 |
| Medium LDL | -0.05 (0.02) | 0.02 | 0.02 |
| Small LDL | -0.02 (0.02) | 0.34 | 0.19 |
| Very large HDL | 0.10 (0.02) | 1.50 X 10^-6 | 4.85 X 10^-6 |
| Large HDL | 0.10 (0.02) | 6.19 X 10^-7 | 2.26 X 10^-6 |
| Medium HDL | 0.16 (0.02) | 1.33 X 10^-15 | <1.0 X 10^-11 |
| Small HDL | 0.16 (0.02) | 2.92 X 10^-13 | <1.0 X 10^-11 |
| **Particles in lipoproteins** |  |  |  |
| Extremely large VLDL | -0.01 (0.02) | 0.58 | 0.27 |
| Very large VLDL | -0.04 (0.02) | 0.09 | 0.07 |
| Large VLDL | -0.04 (0.02) | 0.07 | 0.06 |
| Medium VLDL | -0.04 (0.02) | 0.17 | 0.11 |
| Small VLDL | -0.04 (0.02) | 0.12 | 0.08 |
| Very small VLDL | -0.06 (0.02) | 0.01 | 0.01 |
| IDL | -0.01 (0.02) | 0.73 | 0.32 |
| Large LDL | 0.02 (0.02) | 0.30 | 0.17 |
| Medium LDL | 0.02 (0.02) | 0.44 | 0.22 |
| Small LDL | 0.00 (0.02) | 0.95 | 0.38 |
| Very large HDL | 0.00 (0.02) | 0.93 | 0.38 |
| Large HDL | 0.08 (0.02) | 0.00 | 0.00 |
| Medium HDL | 0.09 (0.02) | 0.00 | 0.00 |
| Small HDL | 0.14 (0.02) | 0.02 | 0.02 |
| **Total cholesterol in lipoproteins** |  |  |  |
| Extremely large VLDL | -0.02 (0.02) | 0.50 | 0.24 |
| Very large VLDL | -0.04 (0.02) | 0.06 | 0.05 |
| Large VLDL | -0.05 (0.02) | 0.03 | 0.03 |
| Medium VLDL | -0.02 (0.02) | 0.37 | 0.20 |
| Small VLDL | -0.04 (0.02) | 0.06 | 0.05 |
| Very small VLDL | 0.04 (0.02) | 0.05 | 0.05 |
| IDL | -0.05 (0.02) | 0.03 | 0.03 |
| Large LDL | 0.01 (0.02) | 0.56 | 0.27 |
| Medium LDL | -0.01 (0.02) | 0.68 | 0.30 |
| Small LDL | 0.01 (0.02) | 0.56 | 0.27 |
| Very large HDL | 0.10 (0.02) | 0.00 | 0.00 |
| Large HDL | 0.11 (0.02) | 0.00 | 0.00 |
| Medium HDL | 0.16 (0.02) | 0.00 | 0.00 |
| Small HDL | 0.14 (0.02) | 0.00 | 0.00 |
| **Apolipoproteins** |  |  |  |
| Apolipoprotein A1 | 0.17 (0.02) | 0.00 | 0.00 |
| Apolipoprotein B | -0.02 (0.02) | 0.29 | 0.17 |
| Apo B/Apo A1 | -0.11 (0.02) | 0.00 | 0.00 |
| **Triacylglycerol in lipoproteins** |  |  |  |
| Extremely large VLDL | -0.01 (0.02) | 0.54 | 0.26 |
| Very large VLDL | -0.04 (0.02) | 0.09 | 0.07 |
| Large VLDL | -0.04 (0.02) | 0.10 | 0.07 |
| Medium VLDL | -0.03 (0.02) | 0.20 | 0.12 |
| Small VLDL | -0.05 (0.02) | 0.02 | 0.02 |
| Very small VLDL | 0.04 (0.02) | 0.13 | 0.09 |
| IDL | 0.05 (0.02) | 0.05 | 0.05 |
| Large LDL | 0.00 (0.02) | 1.00 | 0.39 |
| Medium LDL | -0.01 (0.02) | 0.47 | 0.23 |
| Small LDL | -0.01 (0.02) | 0.58 | 0.27 |
| Very large HDL | 0.11 (0.02) | 0.0003 | 0.0006 |
| Large HDL | 0.11 (0.02) | 7.56 X 10^-5 | 1.81 X 10^-4 |
| Medium HDL | 0.06 (0.02) | 0.01 | 0.01 |
| Small HDL | 0.00 (0.02) | 1.00 | 0.39 |
| **Lipoprotein particle size** |  |  |  |
| VLDL particle size | -0.05 (0.02) | 0.01 | 0.01 |
| LDL particle size | 0.02 (0.01) | 0.009 | 0.01 |
| HDL particle size | 0.07 (0.02) | 0.0009 | 0.01 |
| **Total cholesterol in lipoproteins (%)** |  |  |  |
| Very large VLDL | -0.04 (0.02) | 0.10 | 0.07 |
| Large VLDL | -0.05 (0.02) | 0.02 | 0.02 |
| Medium VLDL | 0.01 (0.02) | 0.53 | 0.26 |
| Small VLDL | -0.02 (0.02) | 0.35 | 0.19 |
| Very small VLDL | 0.08 (0.02) | 0.0005 | 0.0009 |
| IDL | 0.02 (0.02) | 0.38 | 0.20 |
| Large LDL | -0.05 (0.02) | 0.01 | 0.01 |
| Medium LDL | -0.09 (0.02) | 3.08 X 10^-7 | 1.29 X 10^-6 |
| Small LDL | -0.01 (0.02) | 0.71 | 0.31 |
| Very large HDL | -0.02 (0.02) | 0.33 | 0.19 |
| Large HDL | 0.04 (0.02) | 0.14 | 0.09 |
| Medium HDL | -0.35 (0.02) | 0.003 | 0.005 |
| Small HDL | 0.00 (0.02) | 0.96 | 0.38 |
| **Phospholipids in lipoproteins (%)** |  |  |  |
| Very large VLDL | -0.03 (0.02) | 0.30 | 0.17 |
| Large VLDL | -0.02 (0.02) | 0.49 | 0.24 |
| Medium VLDL | 0.04 (0.02) | 0.07 | 0.06 |
| Small VLDL | 0.03 (0.02) | 0.16 | 0.10 |
| Very small VLDL | -0.02 (0.02) | 0.44 | 0.22 |
| IDL | 0.02 (0.02) | 0.30 | 0.17 |
| Large LDL | 0.08 (0.02) | 4.33 X 10^-5 | 0.0001 |
| Medium LDL | 0.12 (0.02) | 2.044 X 10^-10 | 1.20 X 10^-9 |
| Small LDL | 0.10 (0.02) | 2.04 X 10^-7 | 9.54 X 10^-7 |
| Very large HDL | 0.07 (0.02) | 0.003 | 0.005 |
| Large HDL | 0.00 (0.02) | 0.86 | 0.36 |
| Medium HDL | -0.07 (0.02) | 0.002 | 0.004 |
| Small HDL | 0.05 (0.02) | 0.02 | 0.02 |
| **Cholesterol esters in lipoproteins (%)** | |  |  |
| Very large VLDL | -0.04 (0.02) | 0.11 | 0.08 |
| Large VLDL | -0.05 (0.02) | 0.03 | 0.03 |
| Medium VLDL | 0.02 (0.02) | 0.47 | 0.23 |
| Small VLDL | -0.03 (0.02) | 0.23 | 0.14 |
| Very small VLDL | 0.09 (0.02) | 0.0001 | 0.0002 |
| IDL | 0.02 (0.02) | 0.32 | 0.18 |
| Large LDL | -0.05 (0.02) | 0.01 | 0.01 |
| Medium LDL | -0.10 (0.02) | 2.55 X 10^-7 | 1.13 X 10^-6 |
| Small LDL | -0.07 (0.02) | 0.0006 | 0.001 |
| Very large HDL | 0.02 (0.02) | 0.04 | 0.04 |
| Large HDL | 0.03 (0.02) | 0.18 | 0.11 |
| Medium HDL | 0.06 (0.02) | 0.01 | 0.01 |
| Small HDL | 0.00 (0.02) | 0.87 | 0.36 |
| **Free cholesterol in lipoproteins (%)** |  |  |  |
| Very large VLDL | -0.03 (0.02) | 0.26 | 0.16 |
| Large VLDL | -0.04 (0.02) | 0.08 | 0.06 |
| Medium VLDL | 0.00 (0.02) | 0.95 | 0.38 |
| Small VLDL | 0.00 (0.02) | 0.79 | 0.34 |
| Very small VLDL | -0.04 (0.02) | 0.08 | 0.06 |
| IDL | 0.00 (0.02) | 0.92 | 0.38 |
| Large LDL | 0.01 (0.02) | 0.59 | 0.26 |
| Medium LDL | 0.04 (0.02) | 0.08 | 0.06 |
| Small LDL | -0.02 (0.02) | 0.32 | 0.17 |
| Very large HDL | -0.05 (0.02) | 0.02 | 0.02 |
| Large HDL | 0.02 (0.02) | 0.34 | 0.18 |
| Medium HDL | 0.11 (0.02) | 6.02 X 10^-8 | 2.97 X 10^-7 |
| Small HDL | 0.00 (0.02) | 0.87 | 0.36 |
| **Triacylglycerol in lipoproteins (%)** |  |  |  |
| Very large VLDL | -0.04 (0.02) | 0.13 | 0.09 |
| Large VLDL | -0.01 (0.02) | 0.69 | 0.31 |
| Medium VLDL | -0.02 (0.02) | 0.45 | 0.23 |
| Small VLDL | -0.03 (0.02) | 0.16 | 0.10 |
| Very small VLDL | -0.07 (0.02) | 0.003 | 0.004 |
| IDL | -0.03 (0.02) | 0.26 | 0.16 |
| Large LDL | 0.02 (0.02) | 0.37 | 0.20 |
| Medium LDL | 0.04 (0.02) | 0.12 | 0.08 |
| Small LDL | 0.00 (0.02) | 0.87 | 0.36 |
| Very large HDL | -0.03 (0.02) | 0.15 | 0.10 |
| Large HDL | -0.06 (0.02) | 0.01 | 0.01 |
| Medium HDL | -0.05 (0.02) | 0.06 | 0.05 |
| Small HDL | -0.06 (0.02) | 0.08 | 0.06 |
| **Branched-chain amino acids** |  |  |  |
| Isoleucine | -0.07 (0.02) | 0.005 | 0.007 |
| Leucine | -0.03 (0.03) | 0.02 | 0.02 |
| Valine | -0.04 (0.02) | 0.08 | 0.06 |
| **Aromatic amino acids** |  |  |  |
| Phenylalanine | -0.03 (0.02) | 0.25 | 0.15 |
| Tyrosine | 0.04 (0.02) | 0.12 | 0.08 |
| Histidine | 0.02 (0.02) | 0.40 | 0.21 |
| **Other amino acids** |  |  |  |
| Alanine | -0.03 (0.02) | 0.20 | 0.12 |
| Glutamine | 0.01 (0.02) | 0.68 | 0.30 |
| **Ketone bodies** |  |  |  |
| Acetoacetate | 0.00 (0.02) | 0.87 | 0.36 |
| 3-hydroxybutyrate | -0.02 (0.02) | 0.34 | 0.19 |
| **Miscellaneous** |  |  |  |
| Creatinine | 0.00 (0.02) | 0.94 | 0.38 |
| Albumin | 0.06 (0.02) | 0.0006 | 0.001 |
| Acetate | -0.04 (0.02) | 0.08 | 0.06 |
| Citrate | -0.06 (0.02) | 0.01 | 0.01 |
| Glycoprotein acetyls | -0.09 (0.02) | 2.1*10^-4^ | 4.5*10^-4^ |
| **Fatty acids** |  |  |  |
| Total fatty acids | 0.02 (0.02) | 0.46 | 0.23 |
| Degree of unsaturation | 0.02 (0.02) | 0.33 | 0.19 |
| DHA | 0.06 (0.02) | 0.01 | 0.01 |
| Linoleic acid | 0.00 (0.02) | 0.95 | 0.38 |
| Saturated fatty acids | 0.03 (0.02) | 0.17 | 0.11 |
| n-3 fatty acids | 0.04 (0.02) | 0.08 | 0.06 |
| n-6 fatty acids | 0.02 (0.02) | 0.39 | 0.20 |
| Monounsaturated fatty acids | -0.01 (0.02) | 0.83 | 0.35 |
| Polyunsaturated fatty acids | 0.02 (0.02) | 0.30 | 0.17 |
| **Fatty acid ratios** |  |  |  |
| Saturated fatty acids (%) | 0.07 (0.02) | 0.003 | 0.005 |
| Monounsaturated fatty acids (%) | -0.06 (0.02) | 0.01 | 0.01 |
| Polyunsaturated fatty acids (%) | 0.01 (0.02) | 0.74 | 0.32 |
| n-6 fatty acids (%) | 0.00 (0.02) | 0.96 | 0.38 |
| Linoleic acid (%) | -0.04 (0.02) | 0.12 | 0.08 |
| n-3 fatty acids (%) | 0.04 (0.02) | 0.09 | 0.07 |
| DHA (%) | 0.06 (0.02) | 0.01 | 0.01 |
| **Phospholipids** |  |  |  |
| Sphingomyelins | 0.07 (0.02) | 0.003 | 0.005 |
| Total cholines | 0.09 (0.02) | 0.0001 | 0.0003 |
| Phosphatidylcholines | -0.03 (0.03) | 0.31 | 0.18 |
| Total phosphoglycerides | -0.08 (0.02) | 5.09 X 10^-5 | 0.0001 |
| TG/PG | 0.09 (0.02) | 0.0004 | 0.0008 |
| Significance determined at p<0.05 and q<0.10. Linear regression, comparing metabolites (outcome) with alcohol consumption per week on a continuous scale (exposure), models adjusted for age, sex and study (pilot or larger sample). | | | |

**Supplementary Table 4.** Metabolite measures associated with moderate to vigorous physical activity in the BCGP study sample (N=1,319)

|  | | | |
| --- | --- | --- | --- |
|  | ß (SE) | p-value | q-value |
| **Composite lipid measures** |  |  |  |
| Total serum cholesterol | -1.13 (4.50) | 0.80 | 0.88 |
| Esterified cholesterol | -0.79 (4.45) | 0.86 | 0.89 |
| Free cholesterol | -1.29 (4.59) | 0.26 | 0.88 |
| Remnant cholesterol | -3.32 (4.84) | 0.50 | 0.88 |
| VLDL cholesterol | -3.76 (0.50) | 0.46 | 0.88 |
| LDL cholesterol | -2.85 (3.98) | 0.48 | 0.88 |
| HDL cholesterol | 3.98 (3.23) | 0.22 | 0.88 |
| HDL2 cholesterol | 4.05 (3.19) | 0.21 | 0.88 |
| HDL3 cholesterol | 1.74 (3.15) | 0.58 | 0.88 |
| Total triacylglycerol | 0.32 (0.03) | 0.78 | 0.88 |
| Total triacylglycerol in VLDL | -2.69 (5.24) | 0.61 | 0.88 |
| Total triacylglycerol in LDL | -3.38 (4.86) | 0.49 | 0.88 |
| Total triacylglycerol in HDL | 0.55 (4.54) | 0.90 | 0.90 |
| **Total lipids in lipoproteins** |  |  |  |
| Extremely large VLDL | -4.88 (4.61) | 0.29 | 0.88 |
| Very large VLDL | -2.70 (4.31) | 0.53 | 0.88 |
| Large VLDL | -3.31 (4.54) | 0.47 | 0.88 |
| Medium VLDL | -3.88 (4.84) | 0.43 | 0.88 |
| Small VLDL | -2.96 (4.96) | 0.55 | 0.88 |
| Very small VLDL | 0.08 (4.64) | 0.99 | 0.91 |
| IDL | -1.00 (3.60) | 0.78 | 0.88 |
| Large LDL | -2.44 (4.00) | 0.54 | 0.88 |
| Medium LDL | -2.48 (4.42) | 0.58 | 0.88 |
| Small LDL | -1.91 (4.77) | 0.69 | 0.88 |
| Very large HDL | 3.01 (3.27) | 0.36 | 0.88 |
| Large HDL | 2.75 (3.31) | 0.41 | 0.88 |
| Medium HDL | 3.37 (3.00) | 0.26 | 0.88 |
| Small HDL | 2.02 (3.94) | 0.61 | 0.88 |
| **Phospholipids in lipoproteins** |  |  |  |
| Extremely large VLDL | -5.19 (4.52) | 0.26 | 0.88 |
| Very large VLDL | -1.87 (4.05) | 0.65 | 0.88 |
| Large VLDL | -2.89 (4.39) | 0.51 | 0.88 |
| Medium VLDL | -3.49 (4.83) | 0.47 | 0.88 |
| Small VLDL | -2.67 (4.71) | 0.57 | 0.88 |
| Very small VLDL | 0.67 (4.53) | 0.88 | 0.90 |
| IDL | -0.15 (3.48) | 0.97 | 0.91 |
| Large LDL | -1.89 (3.99) | 0.64 | 0.88 |
| Medium LDL | -2.21 (4.49) | 0.64 | 0.88 |
| Small LDL | -1.21 (4.86) | 0.80 | 0.88 |
| Very large HDL | 2.25 (3.12) | 0.47 | 0.88 |
| Large HDL | 2.35 (3.22) | 0.47 | 0.88 |
| Medium HDL | 3.19 (2.95) | 0.28 | 0.88 |
| Small HDL | 2.08 (4.26) | 0.63 | 0.88 |
| **Cholesterol esters in lipoproteins** |  |  |  |
| Extremely large VLDL | -5.31 (4.57) | 0.25 | 0.88 |
| Very large VLDL | -3.29 (4.28) | 0.44 | 0.88 |
| Large VLDL | -3.57 (4.17) | 0.40 | 0.88 |
| Medium VLDL | -3.33 (3.72) | 0.37 | 0.88 |
| Small VLDL | -2.66 (4.37) | 0.55 | 0.88 |
| Very small VLDL | -0.24 (3.58) | 0.95 | 0.90 |
| IDL | -1.80 (3.29) | 0.59 | 0.88 |
| Large LDL | -3.09 (3.95) | 0.44 | 0.88 |
| Medium LDL | -2.90 (4.29) | 0.50 | 0.88 |
| Small LDL | -2.05 (4.50) | 0.65 | 0.88 |
| Very large HDL | 2.42 (3.21) | 0.45 | 0.88 |
| Large HDL | 2.10 (3.36) | 0.53 | 0.88 |
| Medium HDL | 3.18 (3.16) | 0.32 | 0.88 |
| Small HDL | 2.13 (2.76) | 0.44 | 0.88 |
| **Free cholesterol in lipoproteins** |  |  |  |
| Extremely large VLDL | -5.00 (4.55) | 0.28 | 0.88 |
| Very large VLDL | -2.80 (4.15) | 0.50 | 0.88 |
| Large VLDL | -2.94 (4.28) | 0.49 | 0.88 |
| Medium VLDL | -3.86 (4.76) | 0.42 | 0.88 |
| Small VLDL | -3.21 (4.74) | 0.50 | 0.88 |
| Very small VLDL | -0.36 (4.73) | 0.94 | 0.90 |
| IDL | -1.13 (3.26) | 0.73 | 0.88 |
| Large LDL | -2.16 (3.30) | 0.51 | 0.88 |
| Medium LDL | -2.47 (3.68) | 0.50 | 0.88 |
| Small LDL | -2.53 (3.91) | 0.52 | 0.88 |
| Very large HDL | 2.82 (3.32) | 0.40 | 0.88 |
| Large HDL | 2.26 (3.21) | 0.48 | 0.88 |
| Medium HDL | 3.41 (3.05) | 0.27 | 0.88 |
| Small HDL | 3.50 (4.00) | 0.39 | 0.88 |
| **Particles in lipoproteins** |  |  |  |
| Extremely large VLDL | -4.86 (4.63) | 0.30 | 0.88 |
| Very large VLDL | -2.17 (4.34) | 0.53 | 0.88 |
| Large VLDL | -3.31 (4.57) | 0.47 | 0.88 |
| Medium VLDL | -3.84 (4.84) | 0.43 | 0.88 |
| Small VLDL | -2.84 (4.96) | 0.57 | 0.88 |
| Very small VLDL | 0.08 (4.64) | 0.99 | 0.91 |
| IDL | -1.03 (3.71) | 0.78 | 0.88 |
| Large LDL | -2.43 (4.06) | 0.55 | 0.88 |
| Medium LDL | -2.46 (4.43) | 0.58 | 0.88 |
| Small LDL | -1.20 (2.88) | 0.68 | 0.88 |
| Very large HDL | 3.23 (3.38) | 0.34 | 0.88 |
| Large HDL | 2.95 (3.36) | 0.38 | 0.88 |
| Medium HDL | 3.59 (3.10) | 0.25 | 0.88 |
| Small HDL | 2.33 (3.35) | 0.49 | 0.88 |
| **Total cholesterol in lipoproteins** |  |  |  |
| Extremely large VLDL | -5.19 (4.58) | 0.26 | 0.88 |
| Very large VLDL | -3.21 (4.29) | 0.46 | 0.88 |
| Large VLDL | -3.34 (4.24) | 0.43 | 0.88 |
| Medium VLDL | -3.75 (4.21) | 0.38 | 0.88 |
| Small VLDL | -2.95 (4.64) | 0.53 | 0.88 |
| Very small VLDL | -0.31 (3.89) | 0.94 | 0.90 |
| IDL | -0.59 (1.18) | 0.62 | 0.88 |
| Large LDL | -2.88 (3.79) | 0.45 | 0.88 |
| Medium LDL | -2.84 (4.15) | 0.50 | 0.88 |
| Small LDL | -2.31 (4.29) | 0.59 | 0.88 |
| Very large HDL | 2.73 (3.25) | 0.40 | 0.88 |
| Large HDL | 2.36 (3.37) | 0.48 | 0.88 |
| Medium HDL | 3.22 (3.13) | 0.31 | 0.88 |
| Small HDL | 2.34 (2.87) | 0.42 | 0.88 |
| **Apolipoproteins** |  |  |  |
| Apolipoprotein A1 | 3.97 (3.28) | 0.23 | 0.88 |
| Apolipoprotein B | -3.44 (5.17) | 0.51 | 0.88 |
| Apo B/Apo A1 | -5.07 (4.25) | 0.24 | 0.88 |
| **Triacylglycerol in lipoproteins** |  |  |  |
| Extremely large VLDL | -4.87 (4.50) | 0.28 | 0.88 |
| Very large VLDL | -1.76 (3.71) | 0.64 | 0.88 |
| Large VLDL | -2.99 (4.44) | 0.50 | 0.88 |
| Medium VLDL | -3.50 (4.56) | 0.45 | 0.88 |
| Small VLDL | -2.83 (4.57) | 0.54 | 0.88 |
| Very small VLDL | 0.70 (5.55) | 0.90 | 0.90 |
| IDL | 2.52 (5.84) | 0.67 | 0.88 |
| Large LDL | 2.78 (6.23) | 0.72 | 0.88 |
| Medium LDL | 0.56 (6.29) | 0.93 | 0.90 |
| Small LDL | -1.33 (6.04) | 0.83 | 0.88 |
| Very large HDL | 0.57 (4.02) | 0.89 | 0.90 |
| Large HDL | 1.58 (3.18) | 0.62 | 0.88 |
| Medium HDL | 0.15 (4.35) | 0.97 | 0.91 |
| Small HDL | -1.82 (4.59) | 0.69 | 0.88 |
| **Lipoprotein particle size** |  |  |  |
| VLDL particle size | -3.97 (3.62) | 0.28 | 0.88 |
| LDL particle size | 0.39 (1.12) | 0.73 | 0.88 |
| HDL particle size | 2.57 (2.48) | 0.30 | 0.88 |
| **Total cholesterol in lipoproteins (%)** |  |  |  |
| Very large VLDL | -1.58 (2.77) | 0.57 | 0.88 |
| Large VLDL | -1.85 (2.84) | 0.52 | 0.88 |
| Medium VLDL | -1.15 (2.97) | 0.70 | 0.88 |
| Small VLDL | -1.79 (2.75) | 0.52 | 0.88 |
| Very small VLDL | -1.16 (3.02) | 0.70 | 0.88 |
| IDL | -3.79 (2.65) | 0.16 | 0.88 |
| Large LDL | -4.95 (2.87) | 0.09 | 0.88 |
| Medium LDL | -3.66 (2.33) | 0.12 | 0.88 |
| Small LDL | -2.57 (3.54) | 0.47 | 0.88 |
| Very large HDL | -2.27 (2.91) | 0.44 | 0.88 |
| Large HDL | -9.41 (3.12) | 0.76 | 0.88 |
| Medium HDL | 1.49 (3.87) | 0.70 | 0.88 |
| Small HDL | 0.81 (3.88) | 0.84 | 0.88 |
| **Phospholipids in lipoproteins (%)** |  |  |  |
| Very large VLDL | 0.48 (2.91) | 0.87 | 0.90 |
| Large VLDL | 0.91 (2.61) | 0.73 | 0.88 |
| Medium VLDL | 1.17 (2.89) | 0.69 | 0.88 |
| Small VLDL | 0.61 (3.02) | 0.84 | 0.88 |
| Very small VLDL | 1.65 (2.36) | 0.49 | 0.88 |
| IDL | 3.80 (2.41) | 0.12 | 0.88 |
| Large LDL | 3.94 (3.15) | 0.21 | 0.88 |
| Medium LDL | 2.88 (2.76) | 0.30 | 0.88 |
| Small LDL | 3.25 (3.06) | 0.29 | 0.88 |
| Very large HDL | 0.66 (2.88) | 0.82 | 0.88 |
| Large HDL | -2.61 (2.63) | 0.32 | 0.88 |
| Medium HDL | -1.80 (3.33) | 0.59 | 0.88 |
| Small HDL | 0.62 (2.92) | 0.83 | 0.88 |
| **Cholesterol esters in lipoproteins (%)** |  |  |  |
| Very large VLDL | -0.96 (2.66) | 0.72 | 0.88 |
| Large VLDL | -1.83 (2.66) | 0.49 | 0.88 |
| Medium VLDL | -0.96 (3.04) | 0.75 | 0.88 |
| Small VLDL | -1.43 (2.62) | 0.58 | 0.88 |
| Very small VLDL | -0.73 (3.27) | 0.82 | 0.88 |
| IDL | -4.34 (2.54) | 0.09 | 0.88 |
| Large LDL | 0.72 (4.34) | 0.76 | 0.88 |
| Medium LDL | -3.23 (3.03) | 0.29 | 0.88 |
| Small LDL | -0.94 (2.46) | 0.70 | 0.88 |
| Very large HDL | -1.21 (2.82) | 0.67 | 0.88 |
| Large HDL | -1.01 (3.13) | 0.75 | 0.88 |
| Medium HDL | 1.35 (3.99) | 0.74 | 0.88 |
| Small HDL | 0.48 (3.94) | 0.90 | 0.90 |
| **Free cholesterol in lipoproteins (%)** |  |  |  |
| Very large VLDL | -1.96 (3.38) | 0.56 | 0.88 |
| Large VLDL | -1.28 (3.22) | 0.69 | 0.88 |
| Medium VLDL | -2.53 (2.85) | 0.38 | 0.88 |
| Small VLDL | -1.95 (2.68) | 0.47 | 0.88 |
| Very small VLDL | -1.83 (3.53) | 0.61 | 0.88 |
| IDL | -4.34 (2.54) | 0.09 | 0.88 |
| Large LDL | 0.72 (4.34) | 0.76 | 0.88 |
| Medium LDL | -3.23 (3.03) | 0.29 | 0.88 |
| Small LDL | -0.94 (2.46) | 0.70 | 0.88 |
| Very large HDL | -1.21 (2.82) | 0.67 | 0.88 |
| Large HDL | -1.01 (3.13) | 0.75 | 0.88 |
| Medium HDL | 1.35 (3.99) | 0.74 | 0.88 |
| Small HDL | 0.48 (3.94) | 0.90 | 0.90 |
| **Triacylglycerol in lipoproteins (%)** |  |  |  |
| Very large VLDL | -0.73 (3.11) | 0.81 | 0.88 |
| Large VLDL | 0.21 (2.90) | 0.94 | 0.90 |
| Medium VLDL | -1.45 (2.92) | 0.62 | 0.88 |
| Small VLDL | -1.16 (2.71) | 0.67 | 0.88 |
| Very small VLDL | 0.82 (3.41) | 0.81 | 0.88 |
| IDL | 3.48 (3.79) | 0.36 | 0.88 |
| Large LDL | 4.93 (3.58) | 0.17 | 0.88 |
| Medium LDL | 3.88 (3.34) | 0.25 | 0.88 |
| Small LDL | 0.34 (3.80) | 0.93 | 0.90 |
| Very large HDL | -3.06 (3.48) | 0.38 | 0.88 |
| Large HDL | -3.27 (3.41) | 0.34 | 0.88 |
| Medium HDL | -1.87 (4.04) | 0.65 | 0.88 |
| Small HDL | -2.88 (4.01) | 0.48 | 0.88 |
| **Branched-chain amino acids** |  |  |  |
| Isoleucine | -2.66 (3.68) | 0.47 | 0.88 |
| Leucine | -2.35 (3.48) | 0.50 | 0.88 |
| Valine | -3.57 (2.81) | 0.21 | 0.88 |
| **Aromatic amino acids** |  |  |  |
| Phenylalanine | 0.02 (2.87) | 0.99 | 0.91 |
| Tyrosine | 0.50 (2.82) | 0.88 | 0.90 |
| Histidine | 3.11 (2.70) | 0.25 | 0.88 |
| **Other amino acids** |  |  |  |
| Alanine | -1.63 (2.77) | 0.56 | 0.88 |
| Glutamine | 1.95 (2.86) | 0.50 | 0.88 |
| **Ketone bodies** |  |  |  |
| Acetoacetate | 0.66 (2.97) | 0.82 | 0.88 |
| 3-hydroxybutyrate | -0.89 (2.83) | 0.75 | 0.88 |
| **Miscellaneous** |  |  |  |
| Creatinine | -1.40 (2.36) | 0.55 | 0.88 |
| Albumin | -0.07 (2.17) | 0.98 | 0.91 |
| Acetate | 1.84 (3.19) | 0.56 | 0.88 |
| Citrate | -0.66 (2.89) | 0.82 | 0.88 |
| Glycoprotein acetyls | -4.90 (4.59) | 0.29 | 0.88 |
| **Fatty acids** |  |  |  |
| Total fatty acids | -2.15 (5.62) | 0.70 | 0.88 |
| Degree of unsaturation | 3.40 (3.85) | 0.38 | 0.88 |
| DHA | 1.28 (4.34) | 0.77 | 0.88 |
| Linoleic acid | -2.33 (5.12) | 0.65 | 0.88 |
| Saturated fatty acids | -1.96 (5.62) | 0.73 | 0.88 |
| n-3 fatty acids | 0.41 (4.73) | 0.93 | 0.90 |
| n-6 fatty acids | -1.93 (5.17) | 0.71 | 0.88 |
| Monounsaturated fatty acids | -2.73 (5.60) | 0.63 | 0.88 |
| Polyunsaturated fatty acids | -1.54 (5.22) | 0.77 | 0.88 |
| **Fatty acid ratios** |  |  |  |
| Saturated fatty acids (%) | 0.85 (2.85) | 0.76 | 0.88 |
| Monounsaturated fatty acids (%) | -2.78 (3.90) | 0.48 | 0.88 |
| Polyunsaturated fatty acids (%) | 2.30 (4.10) | 0.58 | 0.88 |
| n-6 fatty acids (%) | 1.38 (4.10) | 0.73 | 0.88 |
| Linoleic acid (%) | 0.06 (3.55) | 0.99 | 0.91 |
| n-3 fatty acids (%) | 3.90 (2.86) | 0.17 | 0.88 |
| DHA (%) | 4.08 (2.95) | 0.17 | 0.88 |
| **Phospholipids** |  |  |  |
| Sphingomyelins | -1.78 (3.97) | 0.65 | 0.88 |
| Total cholines | 0.33 (4.76) | 0.95 | 0.90 |
| Phosphatidylcholines | 0.51 (4.98) | 0.92 | 0.90 |
| Total phosphoglycerides | 0.33 (4.97) | 0.95 | 0.90 |
| TG/PG | -3.61 (4.42) | 0.42 | 0.88 |
| Significance determined at p<0.05 and q<0.10. Linear regression, comparing metabolites (outcome) with moderate and vigorous physical activity (minutes) per week on a continuous scale (exposure), models adjusted for age, sex and study (pilot or larger sample). | | | |

**Supplementary Table 5.** Metabolite measures associated with BMI in the BCGP study sample (N=1,319)

|  | ß (SE) | p-value | | | q-value |
| --- | --- | --- | --- | --- | --- |
| **Composite lipid measures** |  |  | | |  |
| Total serum cholesterol | 0.03 (0.05) | 0.57 | | | 0.05 |
| Esterified cholesterol | 0.05 (0.05) | 0.38 | | | 0.03 |
| Free cholesterol | 0.03 (0.05) | 0.62 | | | 0.05 |
| Remnant cholesterol | -0.24 (0.05) | 1.5* 10^-5^ | | | 1.8*10^-6^ |
| VLDL cholesterol | -0.37 (0.05) | <1.0 *10^-20^ | | | <1.0 *10^-11^ |
| LDL cholesterol | -0.19 (0.05) | 0.001 | | | 6.5*10^-5^ |
| HDL cholesterol | 0.60 (0.05) | <1.0 *10^-20^ | | | <1.0 *10^-11^ |
| HDL2 cholesterol | 0.55 (0.05) | <1.0 *10^-20^ | | | <1.0 *10^-11^ |
| HDL3 cholesterol | 0.50 (0.05) | <1.0 *10^-20^ | | | <1.0 *10^-11^ |
| Total triacylglycerol | -0.57 (0.05) | <1.0 *10^-20^ | | | <1.0 *10^-11^ |
| Total triacylglycerol in VLDL | -0.61 (0.05) | <1.0 *10^-20^ | | | <1.0 *10^-11^ |
| Total triacylglycerol in LDL | -0.34 (0.05) | 4.0* 10^-10^ | | | 1.0*10^-10^ |
| Total triacylglycerol in HDL | -0.16 (0.05) | 0.004 | | | 3.9* 10^-4^ |
| **Total lipids in lipoproteins** |  |  | | |  |
| Extremely large VLDL | -0.60 (0.05) | <1.0 *10^-20^ | | | <1.0 *10^-11^ |
| Very large VLDL | -0.55 (0.05) | <1.0 *10^-20^ | | | <1.0 *10^-11^ |
| Large VLDL | -0.58 (0.05) | <1.0 *10^-20^ | | | <1.0 *10^-11^ |
| Medium VLDL | -0.38 (0.05) | <1.0 *10^-20^ | | | <1.0 *10^-11^ |
| Small VLDL | -0.40 (0.05) | <1.0 *10^-20^ | | | <1.0 *10^-11^ |
| Very small VLDL | -0.07 (0.05) | 0.21 | | | 0.02 |
| IDL | 0.08 (0.05) | 0.12 | | | 0.01 |
| Large LDL | -0.12 (0.05) | 0.02 | | | 0.003 |
| Medium LDL | -0.29 (0.05) | 7.8* 10^-8^ | | | 1.1*10^-8^ |
| Small LDL | -0.23 (0.05) | 2.1* 10^-5^ | | | 2.6*10^-6^ |
| Very large HDL | 0.66 (0.05) | <1.0 *10^-20^ | | | <1.0 *10^-11^ |
| Large HDL | 0.68 (0.05) | <1.0 *10^-20^ | | | <1.0 *10^-11^ |
| Medium HDL | 0.37 (0.05) | <1.0 *10^-20^ | | | <1.0 *10^-11^ |
| Small HDL | -0.24 (0.05) | 5.9* 10^-6^ | | | 7.7*10^-7^ |
| **Phospholipids in lipoproteins** |  |  | | |  |
| Extremely large VLDL | -0.59 (0.05) | <1.0 *10^-20^ | | | <1.0 *10^-11^ |
| Very large VLDL | -0.48 (0.05) | <1.0 *10^-20^ | | | <1.0 *10^-11^ |
| Large VLDL | -0.55 (0.05) | <1.0 *10^-20^ | | | <1.0 *10^-11^ |
| Medium VLDL | -0.35 (0.05) | 2.0 *10^-10^ | | | 1.0 *10^-10^ |
| Small VLDL | -0.31 (0.05) | 2.8*10^-8^ | | | 4.3*10^-9^ |
| Very small VLDL | -0.07 (0.05) | 0.21 | | | 0.02 |
| IDL | 0.10 (0.05) | 0.07 | | | 0.01 |
| Large LDL | -0.06 (0.05) | 0.25 | | | 0.02 |
| Medium LDL | -0.24 (0.05) | 7.7 * 10^-6^ | | | 1.0*10^-6^ |
| Small LDL | -0.15 (0.05) | 0.006 | | | 6.1*10^-4^ |
| Very large HDL | 0.61 (0.05) | <1.0 *10^-20^ | | | <1.0 *10^-11^ |
| Large HDL | 0.63 (0.05) | <1.0 *10^-20^ | | | <1.0 *10^-11^ |
| Medium HDL | 0.27 (0.05) | 3.4* 10^-8^ | | | 5.0*10^-9^ |
| Small HDL | -0.27 (0.05) | 5.3* 10^-7^ | | | 7.2*10^-8^ |
| **Cholesterol esters in lipoproteins** |  |  | | |  |
| Extremely large VLDL | -0.58 (0.05) | <1.0 *10^-20^ | | | <1.0 *10^-11^ |
| Very large VLDL | -0.50 (0.05) | <1.0 *10^-20^ | | | <1.0 *10^-11^ |
| Large VLDL | -0.48 (0.05) | | <1.0 *10^-20^ | <1.0 *10^-11^ | |
| Medium VLDL | -0.01 (0.06) | 0.80 | | | 0.06 |
| Small VLDL | -0.30 (0.05) | 3.0*10^-8^ | | | 4.5*10^-9^ |
| Very small VLDL | 0.11 (0.05) | 0.04 | | | 0.004 |
| IDL | 0.10 (0.05) | 0.08 | | | 0.001 |
| Large LDL | -0.18 (0.05) | 9.6 *10^-4^ | | | 1.1*10^-4^ |
| Medium LDL | -0.36 (0.05) | <1.0 *10^-20^ | | | <1.0 *10^-11^ |
| Small LDL | -0.29 (0.05) | 4.6* 10^-8^ | | | 6.6*10^-9^ |
| Very large HDL | 0.61 (0.05) | <1.0 *10^-20^ | | | <1.0 *10^-11^ |
| Large HDL | 0.67 (0.05) | <1.0 *10^-20^ | | | <1.0 *10^-11^ |
| Medium HDL | 0.50 (0.05) | <1.0 *10^-20^ | | | <1.0 *10^-11^ |
| Small HDL | -0.07 (0.05) | 0.19 | | | 0.02 |
| **Free cholesterol in lipoproteins** |  |  | | |  |
| Extremely large VLDL | -0.59 (0.05) | <1.0 *10^-20^ | | | <1.0 *10^-11^ |
| Very large VLDL | -0.52 (0.05) | <1.0 *10^-20^ | | | <1.0 *10^-11^ |
| Large VLDL | -0.53 (0.05) | <1.0 *10^-20^ | | | <1.0 *10^-11^ |
| Medium VLDL | -0.35 (0.05) | 1.0 *10^-10^ | | | 1.0 *10^-10^ |
| Small VLDL | -0.31 (0.05) | 2.0*10^-8^ | | | 3.1*10^-9^ |
| Very small VLDL | -0.09 (0.05) | 0.10 | | | 0.01 |
| IDL | 0.17 (0.05) | 0.002 | | | 2.6*10^-4^ |
| Large LDL | 0.04 (0.06) | 0.49 | | | 0.04 |
| Medium LDL | -0.12 (0.05) | 0.03 | | | 0.003 |
| Small LDL | -0.12 (0.05) | 0.02 | | | 0.002 |
| Very large HDL | 0.59 (0.05) | <1.0 *10^-20^ | | | <1.0 *10^-11^ |
| Large HDL | 0.66 (0.05) | <1.0 *10^-20^ | | | <1.0 *10^-11^ |
| Medium HDL | 0.44 (0.05) | <1.0 *10^-20^ | | | <1.0 *10^-11^ |
| Small HDL | 0.05 (0.05) | 0.32 | | | 0.03 |
| **Particles in lipoproteins** |  |  | | |  |
| Extremely large VLDL | -0.60 (0.05) | <1.0 *10^-20^ | | | <1.0 *10^-11^ |
| Very large VLDL | -0.55 (0.05) | <1.0 *10^-20^ | | | <1.0 *10^-11^ |
| Large VLDL | -0.58 (0.05) | <1.0 *10^-20^ | | | <1.0 *10^-11^ |
| Medium VLDL | -0.41 (0.05) | <1.0 *10^-20^ | | | <1.0 *10^-11^ |
| Small VLDL | -0.42 (0.05) | <1.0 *10^-20^ | | | <1.0 *10^-11^ |
| Very small VLDL | -0.08 (0.05) | 0.15 | | | 0.014 |
| IDL | 0.07 (0.05) | 0.21 | | | 0.02 |
| Large LDL | -0.14 (0.05) | 0.009 | | | 9.6*10^-4^ |
| Medium LDL | -0.20 (0.05) | 1.7* 10^-8^ | | | 2.6*10^-9^ |
| Small LDL | -0.15 (0.03) | 2.0* 10^-6^ | | | 2.7*10^-7^ |
| Very large HDL | 0.63 (0.05) | <1.0 *10^-20^ | | | <1.0 *10^-11^ |
| Large HDL | 0.70 (0.05) | <1.0 *10^-20^ | | | <1.0 *10^-11^ |
| Medium HDL | 0.45 (0.05) | <1.0 *10^-20^ | | | <1.0 *10^-11^ |
| Small HDL | -0.16 (0.05) | 0.003 | | | 3.2*10^-4^ |
| **Total cholesterol in lipoproteins** |  |  | | |  |
| Extremely large VLDL | -0.59 (0.05) | <1.0 *10^-20^ | | | <1.0 *10^-11^ |
| Very large VLDL | -0.52 (0.05) | <1.0 *10^-20^ | | | <1.0 *10^-11^ |
| Large VLDL | -0.50 (0.05) | <1.0 *10^-20^ | | | <1.0 *10^-11^ |
| Medium VLDL | -0.14 (0.05) | 0.01 | | | 0.001 |
| Small VLDL | -0.31 (0.05) | 1.4*10^-8^ | | | 2.2*10^-9^ |
| Very small VLDL | 0.06 (0.05) | 0.24 | | | 0.02 |
| IDL | 0.04 (0.02) | 0.04 | | | 0.004 |
| Large LDL | -0.13 (0.05) | 0.02 | | | 0.002 |
| Medium LDL | -0.29 (0.05) | 4.3* 10^-8^ | | | 6.2*10^-9^ |
| Small LDL | -0.22 (0.05) | 3.04* 10^-5^ | | | 3.7*10^-6^ |
| Very large HDL | 0.63 (0.05) | <1.0 *10^-20^ | | | <1.0 *10^-11^ |
| Large HDL | 0.69 (0.05) | <1.0 *10^-20^ | | | <1.0 *10^-11^ |
| Medium HDL | 0.50 (0.05) | <1.0 *10^-20^ | | | <1.0 *10^-11^ |
| Small HDL | -0.06 (0.05) | 0.28 | | | 0.02 |
| **Apolipoproteins** |  |  | | |  |
| Apolipoprotein A1 | 0.45 (0.05) | <1.0 *10^-20^ | | | <1.0 *10^-11^ |
| Apolipoprotein B | -0.29 (0.05) | 6.6* 10^-8^ | | | 9.4*10^-9^ |
| Apo B/Apo A1 | -0.49 (0.05) | <1.0 *10^-20^ | | | <1.0 *10^-11^ |
| **Triacylglycerol in lipoproteins** |  |  | | |  |
| Extremely large VLDL | -0.59 (0.05) | <1.0 *10^-20^ | | | <1.0 *10^-11^ |
| Very large VLDL | -0.45 (0.05) | <1.0 *10^-20^ | | | <1.0 *10^-11^ |
| Large VLDL | -0.58 (0.05) | <1.0 *10^-20^ | | | <1.0 *10^-11^ |
| Medium VLDL | -0.53 (0.05) | <1.0 *10^-20^ | | | <1.0 *10^-11^ |
| Small VLDL | -0.48 (0.05) | <1.0 *10^-20^ | | | <1.0 *10^-11^ |
| Very small VLDL | -0.42 (0.05) | <1.0 *10^-20^ | | | <1.0 *10^-11^ |
| IDL | -0.23 (0.05) | 2.0 * 10^-5^ | | | 2.5*10^-6^ |
| Large LDL | -0.26 (0.05) | 2.1* 10^-6^ | | | 1.4*10^-7^ |
| Medium LDL | -0.36 (0.05) | <1.0 *10^-20^ | | | <1.0 *10^-11^ |
| Small LDL | -0.44 (0.05) | <1.0 *10^-20^ | | | <1.0 *10^-11^ |
| Very large HDL | 0.24 (0.05) | 1.0* 10^-5^ | | | 1.3*10^-6^ |
| Large HDL | 0.45 (0.05) | <1.0 *10^-20^ | | | <1.0 *10^-11^ |
| Medium HDL | -0.28 (0.05) | 2.3* 10^-7^ | | | 3.3*10^-8^ |
| Small HDL | -0.53 (0.05) | <1.0 *10^-20^ | | | <1.0 *10^-11^ |
| **Lipoprotein particle size** |  |  | | |  |
| VLDL particle size | -0.55 (0.04) | <1.0 *10^-20^ | | | <1.0 *10^-11^ |
| LDL particle size | 0.14 (0.02) | <1.0 *10^-20^ | | | <1.0 *10^-11^ |
| HDL particle size | 0.53 (0.04) | <1.0 *10^-20^ | | | <1.0 *10^-11^ |
| **Total cholesterol in lipoproteins (%)** |  |  | | |  |
| Very large VLDL | -0.10 (0.06) | 0.07 | | | 0.007 |
| Large VLDL | -0.14 (0.05) | 0.009 | | | 0.001 |
| Medium VLDL | 0.37 (0.05) | <1.0 *10^-20^ | | | <1.0 *10^-11^ |
| Small VLDL | 0.04 (0.05) | 0.46 | | | 0.04 |
| Very small VLDL | 0.38 (0.05) | <1.0 *10^-20^ | | | <1.0 *10^-11^ |
| IDL | 0.19 (0.05) | 4.1* 10^-5^ | | | 4.9*10^-6^ |
| Large LDL | -0.10 (0.04) | 0.02 | | | 0.002 |
| Medium LDL | -0.18 (0.04) | 1.1* 10^-5^ | | | 1.4*10^-6^ |
| Small LDL | -0.01 (0.04) | 0.82 | | | 0.07 |
| Very large HDL | -0.27 (0.05) | 7.3* 10^-7^ | | | 1.0*10^-7^ |
| Large HDL | 0.44 (0.05) | <1.0 *10^-20^ | | | <1.0 *10^-11^ |
| Medium HDL | 0.57 (0.05) | <1.0 *10^-20^ | | | <1.0 *10^-11^ |
| Small HDL | 0.34 (0.05) | <1.0 *10^-20^ | | | <1.0 *10^-11^ |
| **Phospholipids in lipoproteins (%)** |  |  | | |  |
| Very large VLDL | -0.17 (0.06) | 0.002 | | | 2.5*10^-4^ |
| Large VLDL | -0.03 (0.06) | 0.63 | | | 0.05 |
| Medium VLDL | 0.16 (0.05) | 0.003 | | | 3.2*10^-4^ |
| Small VLDL | 0.23 (0.05) | 2.2* 10^-5^ | | | 2.7*10^-6^ |
| Very small VLDL | 0.00 (0.05) | 0.99 | | | 0.08 |
| IDL | 0.06 (0.05) | 0.24 | | | 0.02 |
| Large LDL | 0.34 (0.05) | <1.0 *10^-20^ | | | <1.0 *10^-11^ |
| Medium LDL | 0.35 (0.04) | <1.0 *10^-20^ | | | <1.0 *10^-11^ |
| Small LDL | 0.36 (0.04) | <1.0 *10^-20^ | | | <1.0 *10^-11^ |
| Very large HDL | 0.41 (0.05) | <1.0 *10^-20^ | | | <1.0 *10^-11^ |
| Large HDL | -0.09 (0.06) | 0.10 | | | 0.009 |
| Medium HDL | -0.57 (0.05) | <1.0 *10^-20^ | | | <1.0 *10^-11^ |
| Small HDL | -0.14 (0.04) | 0.002 | | | 1.8*10^-4^ |
| **Cholesterol esters in lipoproteins (%)** | |  | | |  |
| Very large VLDL | 0.02 (0.06) | 0.73 | | | 0.06 |
| Large VLDL | -0.01 (0.06) | 0.88 | | | 0.07 |
| Medium VLDL | 0.37 (0.05) | <1.0 *10^-20^ | | | <1.0 *10^-11^ |
| Small VLDL | -0.09 (0.05) | 0.10 | | | 0.01 |
| Very small VLDL | 0.40 (0.05) | <1.0 *10^-20^ | | | <1.0 *10^-11^ |
| IDL | 0.11 (0.05) | 0.02 | | | 0.002 |
| Large LDL | -0.38 (0.05) | <1.0 *10^-20^ | | | <1.0 *10^-11^ |
| Medium LDL | -0.46 (0.04) | <1.0 *10^-20^ | | | <1.0 *10^-11^ |
| Small LDL | -0.29 (0.04) | 1.0* 10^-10^ | | | <1.0 *10^-11^ |
| Very large HDL | 0.04 (0.06) | 0.43 | | | 0.04 |
| Large HDL | 0.41 (0.05) | <1.0 *10^-20^ | | | <1.0 *10^-11^ |
| Medium HDL | 0.56 (0.05) | <1.0 *10^-20^ | | | <1.0 *10^-11^ |
| Small HDL | 0.28 (0.05) | 3.0* 10^-8^ | | | 4.5*10^-9^ |
| **Free cholesterol in lipoproteins (%)** |  |  | | |  |
| Very large VLDL | -0.36 (0.05) | <1.0 *10^-20^ | | | <1.0 *10^-11^ |
| Large VLDL | -0.32 (0.05) | 3.0* 10^-9^ | | | 5.0*10^-10^ |
| Medium VLDL | -0.06 (0.05) | 0.24 | | | 0.02 |
| Small VLDL | 0.17 (0.05) | 0.001 | | | 1.4*10^-4^ |
| Very small VLDL | -0.13 (0.05) | 0.01 | | | 0.001 |
| IDL | 0.37 (0.05) | <1.0 *10^-20^ | | | <1.0 *10^-11^ |
| Large LDL | 0.54 (0.05) | <1.0 *10^-20^ | | | <1.0 *10^-11^ |
| Medium LDL | 0.50 (0.05) | <1.0 *10^-20^ | | | <1.0 *10^-11^ |
| Small LDL | 0.27 (0.05) | 2.4* 10^-7^ | | | 3.4*10^-8^ |
| Very large HDL | -0.52 (0.05) | <1.0 *10^-20^ | | | <1.0 *10^-11^ |
| Large HDL | 0.23 (0.05) | 3.5* 10^-5^ | | | 4.3*10^-6^ |
| Medium HDL | 0.52 (0.05) | <1.0 *10^-20^ | | | <1.0 *10^-11^ |
| Small HDL | 0.54 (0.05) | <1.0 *10^-20^ | | | <1.0 *10^-11^ |
| **Triacylglycerol in lipoproteins (%)** |  |  | | |  |
| Very large VLDL | -0.31 (0.06) | 3.3* 10^-8^ | | | 4.9*10^-9^ |
| Large VLDL | -0.26 (0.05) | 1.7* 10^-6^ | | | 2.2*10^-7^ |
| Medium VLDL | -0.46 (0.05) | <1.0 *10^-20^ | | | <1.0 *10^-11^ |
| Small VLDL | 0.34 (0.05) | 5.0* 10^-10^ | | | 1.0*10^-10^ |
| Very small VLDL | -0.46 (0.05) | <1.0 *10^-20^ | | | <1.0 *10^-11^ |
| IDL | -0.31 (0.06) | 2.0* 10^-8^ | | | 3.1*10^-9^ |
| Large LDL | -0.12 (0.06) | 0.03 | | | 0.003 |
| Medium LDL | -0.04 (0.06) | 0.52 | | | 0.04 |
| Small LDL | -0.31 (0.06) | 2.9* 10^-8^ | | | 4.3*10^-9^ |
| Very large HDL | -0.49 (0.05) | <1.0 *10^-20^ | | | <1.0 *10^-11^ |
| Large HDL | -0.40 (0.05) | <1.0 *10^-20^ | | | <1.0 *10^-11^ |
| Medium HDL | -0.49 (0.05) | <1.0 *10^-20^ | | | <1.0 *10^-11^ |
| Small HDL | -0.49 (0.05) | <1.0 *10^-20^ | | | <1.0 *10^-11^ |
| **Branched-chain amino acids** |  |  | | |  |
| Isoleucine | -0.27 (0.06) | 7.5* 10^-7^ | | | 1.0* 10^-7^ |
| Leucine | -0.25 (0.05) | 3.3* 10^-6^ | | | 4.4* 10^-7^ |
| Valine | -0.19 (0.05) | 3.6* 10^-4^ | | | 4.3* 10^-5^ |
| **Aromatic amino acids** |  |  | | |  |
| Phenylalanine | -0.05 (0.05) | 0.33 | | | 0.03 |
| Tyrosine | -0.05 (0.06) | 0.33 | | | 0.03 |
| Histidine | 0.21 (0.05) | 1.2* 10^-4^ | | | 1.4*10^-5^ |
| **Other amino acids** |  |  | | |  |
| Alanine | -0.10 (0.05) | 0.06 | | | 0.006 |
| Glutamine | 0.41 (0.05) | <1.0 *10^-20^ | | | <1.0 *10^-11^ |
| **Ketone bodies** |  |  | | |  |
| Acetoacetate | -0.10 (0.05) | 0.05 | | | 0.005 |
| 3-hydroxybutyrate | -0.09 (0.06) | 0.10 | | | 0.01 |
| **Miscellaneous** |  |  | | |  |
| Creatinine | -0.11 (0.05) | 0.02 | | | 0.002 |
| Albumin | 0.05 (0.04) | 0.24 | | | 0.02 |
| Acetate | 0.10 (0.06) | 0.07 | | | 0.007 |
| Citrate | 0.28 (0.05) | 9.6* 10^-8^ | | | 1.3* 10^-8^ |
| Glycoprotein acetyls | -0.66 (0.05) | <1.0 *10^-20^ | | | <1.0 *10^-11^ |
| **Fatty acids** |  |  | | |  |
| Total fatty acids | -0.32 (0.05) | 3.1* 10^-9^ | | | 5.0*10^-10^ |
| Degree of unsaturation | 0.49 (0.05) | <1.0 *10^-20^ | | | <1.0 *10^-11^ |
| DHA | -0.10 (0.05) | 0.07 | | | 0.007 |
| Linoleic acid | -0.03 (0.06) | 0.58 | | | 0.05 |
| Saturated fatty acids | -0.36 (0.05) | <1.0 *10^-20^ | | | <1.0 *10^-11^ |
| n-3 fatty acids | 0.21 (0.05) | 1.2* 10^-4^ | | | 1.4* 10^-5^ |
| n-6 fatty acids | 0.63 (0.05) | <1.0 *10^-20^ | | | <1.0 *10^-11^ |
| Monounsaturated fatty acids | -0.58 (0.05) | <1.0 *10^-20^ | | | <1.0 *10^-11^ |
| Polyunsaturated fatty acids | 0.63 (0.05) | <1.0 *10^-20^ | | | <1.0 *10^-11^ |
| **Fatty acid ratios** |  |  | | |  |
| Saturated fatty acids (%) | -0.18 (0.05) | 5.6* 10^-4^ | | | 6.5* 10^-5^ |
| Monounsaturated fatty acids (%) | -0.46 (0.05) | <1.0 *10^-20^ | | | <1.0 *10^-11^ |
| Polyunsaturated fatty acids (%) | -0.10 (0.05) | 0.07 | | | 0.007 |
| n-6 fatty acids (%) | -0.08 (0.05) | 0.13 | | | 0.01 |
| Linoleic acid (%) | 0.61 (0.05) | <1.0 *10^-20^ | | | <1.0 *10^-11^ |
| n-3 fatty acids (%) | -0.16 (0.05) | 0.003 | | | 3.5* 10^-4^ |
| DHA (%) | 0.22 (0.06) | 9.8* 10^-5^ | | | 1.2*10^-5^ |
| **Phospholipids** |  |  | | |  |
| Sphingomyelins | 0.07 (0.05) | 0.21 | | | 0.02 |
| Total cholines | 0.06 (0.05) | 0.28 | | | 0.02 |
| Phosphatidylcholines | 0.03 (0.05) | 0.55 | | | 0.05 |
| Total phosphoglycerides | -0.02 (0.05) | 0.73 | | | 0.06 |
| TG/PG | -0.61 (0.05) | <1.0 *10^-20^ | | | <1.0 *10^-11^ |
| Significance determined at p<0.05 and q<0.10. Linear regression, comparing metabolites (outcome) with BMI on a categorical scale (exposure), models adjusted for age, sex and study (pilot or larger sample) | | | | | |

**Supplementary Table 6.** Metabolite measures associated with fruit and vegetable consumption in the BCGP study sample (N=1,319)

|  | ß (SE) | p-value | q-value |
| --- | --- | --- | --- |
| **Composite lipid measures** |  |  |  |
| Total serum cholesterol | -0.03 (0.05) | 0.57 | 0.26 |
| Esterified cholesterol | -0.01 (0.05) | 0.82 | 0.33 |
| Free cholesterol | -0.02 (0.05) | 0.75 | 0.31 |
| Remnant cholesterol | -0.10 (0.05) | 0.07 | 0.05 |
| VLDL cholesterol | -0.12 (0.05) | 0.03 | 0.03 |
| LDL cholesterol | -0.08 (0.05) | 0.13 | 0.08 |
| HDL cholesterol | 0.15 (0.05) | 0.004 | 0.005 |
| HDL2 cholesterol | 0.14 (0.05) | 0.007 | 0.008 |
| HDL3 cholesterol | 0.16 (0.05) | 0.004 | 0.005 |
| Total triacylglycerol | -0.19 (0.05) | 7.4*10^-4^ | 0.002 |
| Total triacylglycerol in VLDL | -0.19 (0.05) | 4.8*10^-4^ | 0.001 |
| Total triacylglycerol in LDL | -0.13 (0.05) | 0.02 | 0.02 |
| Total triacylglycerol in HDL | -0.06 (0.05) | 0.26 | 0.13 |
| **Total lipids in lipoproteins** |  |  |  |
| Extremely large VLDL | -0.24 (0.06) | 2.2*10^-5^ | 2.1*10^-4^ |
| Very large VLDL | -0.19 (0.06) | 4.7*10^-4^ | 1.1*10^-4^ |
| Large VLDL | -0.18 (0.05) | 8.6*10^-4^ | 0.002 |
| Medium VLDL | -0.08 (0.05) | 0.12 | 0.08 |
| Small VLDL | -0.09 (0.05) | 0.12 | 0.07 |
| Very small VLDL | -0.03 (0.05) | 0.52 | 0.24 |
| IDL | -0.03 (0.05) | 0.60 | 0.26 |
| Large LDL | -0.07 (0.05) | 0.18 | 0.10 |
| Medium LDL | -0.12 (0.05) | 0.04 | 0.04 |
| Small LDL | -0.10 (0.05) | 0.07 | 0.05 |
| Very large HDL | 0.20 (0.05) | 1.7*10^-4^ | 5.9*10^-4^ |
| Large HDL | 0.20 (0.05) | 9.4*10^-5^ | 3.8*10^-4^ |
| Medium HDL | 0.06 (0.05) | 0.20 | 0.11 |
| Small HDL | -0.16 (0.05) | 0.002 | 0.004 |
| **Phospholipids in lipoproteins** |  |  |  |
| Extremely large VLDL | -0.24 (0.06) | 1.4*10^-5^ | 2.1*10^-4^ |
| Very large VLDL | -0.17 (0.06) | 0.002 | 0.003 |
| Large VLDL | -0.16 (0.05) | 0.003 | 0.005 |
| Medium VLDL | -0.06 (0.06) | 0.24 | 0.13 |
| Small VLDL | -0.04 (0.06) | 0.43 | 0.20 |
| Very small VLDL | -0.02 (0.05) | 0.76 | 0.32 |
| IDL | -0.01 (0.05) | 0.79 | 0.32 |
| Large LDL | -0.07 (0.05) | 0.23 | 0.12 |
| Medium LDL | -0.12 (0.05) | 0.05 | 0.04 |
| Small LDL | -0.08 (0.05) | 0.15 | 0.09 |
| Very large HDL | 0.17 (0.05) | 0.001 | 0.003 |
| Large HDL | 0.19 (0.05) | 2.9*10^-4^ | 8.2*10^-4^ |
| Medium HDL | 0.02 (0.05) | 0.75 | 0.31 |
| Small HDL | -0.18 (0.05) | 5.7*10^-4^ | 0.001 |
| **Cholesterol esters in lipoproteins** |  |  |  |
| Extremely large VLDL | -0.23 (0.06) | 3.6*10^-5^ | 2.2*10^-4^ |
| Very large VLDL | -0.16 (0.06) | 0.003 | 0.005 |
| Large VLDL | -0.14 (0.05) | 0.01 | 0.01 |
| Medium VLDL | 0.05 (0.06) | 0.41 | 0.20 |
| Small VLDL | -0.08 (0.05) | 0.14 | 0.08 |
| Very small VLDL | 0.01 (0.05) | 0.84 | 0.33 |
| IDL | -0.03 (0.05) | 0.57 | 0.26 |
| Large LDL | -0.09 (0.05) | 0.12 | 0.07 |
| Medium LDL | -0.12 (0.05) | 0.03 | 0.03 |
| Small LDL | -0.10 (0.05) | 0.05 | 0.04 |
| Very large HDL | 0.20 (0.05) | 1.5*10^-4^ | 5.4*10^-4^ |
| Large HDL | 0.22 (0.05) | 4.4*10^-5^ | 2.2*10^-4^ |
| Medium HDL | 0.12 (0.05) | 0.02 | 0.02 |
| Small HDL | -0.09 (0.05) | 0.09 | 0.06 |
| **Free cholesterol in lipoproteins** |  |  |  |
| Extremely large VLDL | -0.24 (0.06) | 2.8 *10^-5^ | 2.1*10^-4^ |
| Very large VLDL | -0.18 (0.06) | 8.3*10^-4^ | 0.002 |
| Large VLDL | -0.16 (0.05) | 0.003 | 0.005 |
| Medium VLDL | -0.08 (0.05) | 0.14 | 0.08 |
| Small VLDL | -0.07 (0.05) | 0.23 | 0.12 |
| Very small VLDL | -0.04 (0.05) | 0.45 | 0.21 |
| IDL | 0.00 (0.05) | 0.93 | 0.35 |
| Large LDL | -0.01 (0.06) | 0.82 | 0.33 |
| Medium LDL | -0.05 (0.05) | 0.31 | 0.16 |
| Small LDL | -0.06 (0.05) | 0.27 | 0.14 |
| Very large HDL | 0.17 (0.05) | 0.001 | 0.003 |
| Large HDL | 0.20 (0.05) | 1.0*10^-4^ | 4.9*10^-4^ |
| Medium HDL | 0.09 (0.05) | 0.06 | 0.05 |
| Small HDL | -0.04 (0.05) | 0.38 | 0.18 |
| **Particles in lipoproteins** |  |  |  |
| Extremely large VLDL | -0.24 (0.06) | 2.6*10^-5^ | 2.1*10^-4^ |
| Very large VLDL | -0.19 (0.05) | 4.4*10^-4^ | 1.1*10^-4^ |
| Large VLDL | -0.18 (0.05) | 7.6*10^-4^ | 0.002 |
| Medium VLDL | -0.09 (0.05) | 0.10 | 0.07 |
| Small VLDL | -0.09 (0.05) | 0.11 | 0.07 |
| Very small VLDL | -0.04 (0.05) | 0.49 | 0.23 |
| IDL | -0.03 (0.05) | 0.55 | 0.25 |
| Large LDL | -0.08 (0.05) | 0.16 | 0.09 |
| Medium LDL | -0.11 (0.05) | 0.04 | 0.03 |
| Small LDL | -0.06 (0.03) | 0.05 | 0.04 |
| Very large HDL | 0.19 (0.05) | 2.6*10^-4^ | 8.1*10^-4^ |
| Large HDL | 0.21 (0.05) | 3.8*10^-5^ | 2.2*10^-4^ |
| Medium HDL | 0.09 (0.05) | 0.06 | 0.05 |
| Small HDL | -0.12 (0.05) | 0.03 | 0.03 |
| **Total cholesterol in lipoproteins** |  |  |  |
| Extremely large VLDL | -0.23 (0.06) | 3.4*10^-4^ | 2.2*10^-4^ |
| Very large VLDL | -0.17 (0.06) | 0.002 | 0.003 |
| Large VLDL | -0.15 (0.05) | 0.006 | 0.008 |
| Medium VLDL | -0.01 (0.06) | 0.92 | 0.35 |
| Small VLDL | -0.08 (0.05) | 0.15 | 0.09 |
| Very small VLDL | 0.00 (0.05) | 0.95 | 0.36 |
| IDL | -0.01 (0.02) | 0.67 | 0.28 |
| Large LDL | -0.07 (0.05) | 0.21 | 0.11 |
| Medium LDL | -0.10 (0.05) | 0.06 | 0.04 |
| Small LDL | -0.09 (0.05) | 0.10 | 0.06 |
| Very large HDL | 0.20 (0.05) | 1.5*10^-4^ | 5.4*10^-4^ |
| Large HDL | 0.21 (0.05) | 4.7*10^-5^ | 2.2*10^-4^ |
| Medium HDL | 0.12 (0.05) | 0.02 | 0.02 |
| Small HDL | -0.09 (0.05) | 0.10 | 0.06 |
| **Apolipoproteins** |  |  |  |
| Apolipoprotein A1 | 0.08 (0.05) | 0.10 | 0.06 |
| Apolipoprotein B | -0.10 (0.05) | 0.06 | 0.05 |
| Apo B/Apo A1 | -0.13 (0.05) | 0.01 | 0.02 |
| **Triacylglycerol in lipoproteins** |  |  |  |
| Extremely large VLDL | -0.24 (0.06) | 2.0*10^-5^ | 2.1*10^-4^ |
| Very large VLDL | -0.16 (0.06) | 0.003 | 0.005 |
| Large VLDL | -0.19 (0.05) | 4.2*10^-4^ | 0.001 |
| Medium VLDL | -0.12 (0.05) | 0.03 | 0.03 |
| Small VLDL | -0.08 (0.05) | 0.13 | 0.08 |
| Very small VLDL | -0.12 (0.05) | 0.04 | 0.03 |
| IDL | -0.08 (0.05) | 0.14 | 0.08 |
| Large LDL | -0.12 (0.05) | 0.04 | 0.03 |
| Medium LDL | -0.13 (0.05) | 0.02 | 0.02 |
| Small LDL | -0.16 (0.05) | 0.003 | 0.005 |
| Very large HDL | 0.08 (0.06) | 0.16 | 0.09 |
| Large HDL | 0.16 (0.05) | 0.002 | 0.004 |
| Medium HDL | -0.10 (0.06) | 0.07 | 0.05 |
| Small HDL | -0.16 (0.05) | 0.003 | 0.005 |
| **Lipoprotein particle size** |  |  |  |
| VLDL particle size | -0.17 (0.02) | 2.7*10^-4^ | 8.1*10^-4^ |
| LDL particle size | 0.03 (0.04) | 0.06 | 0.04 |
| HDL particle size | 0.16 (0.05) | 4.1*10^-5^ | 2.2*10^-4^ |
| **Total cholesterol in lipoproteins (%)** |  |  |  |
| Very large VLDL | -0.03 (0.06) | 0.58 | 0.26 |
| Large VLDL | -0.03 (0.06) | 0.64 | 0.27 |
| Medium VLDL | 0.16 (0.05) | 0.004 | 0.006 |
| Small VLDL | 0.00 (0.05) | 0.99 | 0.37 |
| Very small VLDL | 0.08 (0.05) | 0.12 | 0.07 |
| IDL | 0.02 (0.05) | 0.74 | 0.31 |
| Large LDL | -0.01 (0.04) | 0.81 | 0.33 |
| Medium LDL | -0.01 (0.04) | 0.86 | 0.34 |
| Small LDL | 0.03 (0.04) | 0.43 | 0.20 |
| Very large HDL | -0.03 (0.06) | 0.62 | 0.27 |
| Large HDL | 0.19 (0.06) | 4.8*10^-4^ | 0.001 |
| Medium HDL | 0.20 (0.05) | 2.3*10^-4^ | 7.4*10^-4^ |
| Small HDL | 0.13 (0.05) | 0.009 | 0.01 |
| **Phospholipids in lipoproteins (%)** |  |  |  |
| Very large VLDL | -0.08 (0.06) | 0.16 | 0.09 |
| Large VLDL | 0.01 (0.06) | 0.84 | 0.33 |
| Medium VLDL | 0.15 (0.05) | 0.007 | 0.009 |
| Small VLDL | 0.13 (0.06) | 0.02 | 0.02 |
| Very small VLDL | 0.05 (0.05) | 0.30 | 0.15 |
| IDL | 0.07 (0.05) | 0.16 | 0.09 |
| Large LDL | 0.08 (0.05) | 0.10 | 0.06 |
| Medium LDL | 0.05 (0.05) | 0.30 | 0.15 |
| Small LDL | 0.08 (0.05) | 0.08 | 0.06 |
| Very large HDL | 0.11 (0.06) | 0.04 | 0.03 |
| Large HDL | 0.00 (0.06) | 0.95 | 0.36 |
| Medium HDL | -0.22 (0.05) | 1.4*10^-5^ | 2.1*10^-4^ |
| Small HDL | -0.11 (0.04) | 0.02 | 0.02 |
| **Cholesterol esters in lipoproteins (%)** | |  |  |
| Very large VLDL | 0.01 (0.06) | 0.82 | 0.33 |
| Large VLDL | 0.02 (0.06) | 0.70 | 0.29 |
| Medium VLDL | 0.17 (0.06) | 0.002 | 0.003 |
| Small VLDL | 0.01 (0.05) | 0.90 | 0.35 |
| Very small VLDL | 0.09 (0.05) | 0.07 | 0.05 |
| IDL | -0.03 (0.05) | 0.58 | 0.26 |
| Large LDL | -0.11 (0.05) | 0.02 | 0.02 |
| Medium LDL | -0.10 (0.05) | 0.03 | 0.03 |
| Small LDL | -0.04 (0.04) | 0.33 | 0.16 |
| Very large HDL | 0.08 (0.06) | 0.17 | 0.09 |
| Large HDL | 0.19 (0.06) | 6.8*10^-4^ | 0.002 |
| Medium HDL | 0.19 (0.05) | 3.1*10^-4^ | 8.5*10^-4^ |
| Small HDL | 0.11 (0.05) | 0.03 | 0.03 |
| **Free cholesterol in lipoproteins (%)** |  |  |  |
| Very large VLDL | -0.14 (0.06) | 0.01 | 0.02 |
| Large VLDL | -0.09 (0.05) | 0.09 | 0.06 |
| Medium VLDL | 0.04 (0.05) | 0.50 | 0.23 |
| Small VLDL | 0.07 (0.05) | 0.17 | 0.09 |
| Very small VLDL | -0.04 (0.05) | 0.37 | 0.18 |
| IDL | 0.14 (0.05) | 0.005 | 0.007 |
| Large LDL | 0.20 (0.05) | 6.7*10^-5^ | 2.9*10^-4^ |
| Medium LDL | 0.16 (0.05) | 0.001 | 0.003 |
| Small LDL | 0.09 (0.05) | 0.07 | 0.05 |
| Very large HDL | -0.14 (0.05) | 0.01 | 0.012 |
| Large HDL | 0.11 (0.06) | 0.05 | 0.04 |
| Medium HDL | 0.15 (0.05) | 0.001 | 0.002 |
| Small HDL | 0.22 (0.05) | 1.8*10^-5^ | 2.1*10^-4^ |
| **Triacylglycerol in lipoproteins (%)** |  |  |  |
| Very large VLDL | -0.12 (0.06) | 0.03 | 0.03 |
| Large VLDL | -0.12 (0.05) | 0.02 | 0.02 |
| Medium VLDL | -0.07 (0.05) | 0.23 | 0.12 |
| Small VLDL | -0.01 (0.05) | 0.89 | 0.34 |
| Very small VLDL | -0.11 (0.05) | 0.05 | 0.04 |
| IDL | -0.05 (0.06) | 0.40 | 0.19 |
| Large LDL | -0.03 (0.06) | 0.62 | 0.27 |
| Medium LDL | -0.01 (0.06) | 0.88 | 0.34 |
| Small LDL | -0.10 (0.06) | 0.08 | 0.06 |
| Very large HDL | -0.13 (0.05) | 0.02 | 0.02 |
| Large HDL | -0.06 (0.06) | 0.28 | 0.14 |
| Medium HDL | -0.13 (0.05) | 0.02 | 0.02 |
| Small HDL | -0.12 (0.05) | 0.03 | 0.03 |
| **Branched-chain amino acids** |  |  |  |
| Isoleucine | -0.02 (0.05) | 0.76 | 0.31 |
| Leucine | 0.00 (0.05) | 0.99 | 0.37 |
| Valine | 0.08 (0.05) | 0.14 | 0.08 |
| **Aromatic amino acids** |  |  |  |
| Phenylalanine | 0.10 (0.05) | 0.08 | 0.05 |
| Tyrosine | 0.06 (0.05) | 0.25 | 0.13 |
| Histidine | 0.09 (0.06) | 0.11 | 0.07 |
| **Other amino acids** |  |  |  |
| Alanine | 0.04 (0.05) | 0.41 | 0.20 |
| Glutamine | 0.10 (0.05) | 0.06 | 0.04 |
| **Ketone bodies** |  |  |  |
| Acetoacetate | 0.08 (0.06) | 0.12 | 0.08 |
| 3-hydroxybutyrate | -0.09 (0.05) | 0.10 | 0.07 |
| **Miscellaneous** |  |  |  |
| Creatinine | -0.03 (0.04) | 0.46 | 0.22 |
| Albumin | -0.06 (0.06) | 0.13 | 0.08 |
| Acetate | 0.14 (0.05) | 0.01 | 0.02 |
| Citrate | 0.09 (0.05) | 0.09 | 0.06 |
| Glycoprotein acetyls | -0.20 (0.05) | 2.9*10^-4^ | 8.2*10^-4^ |
| **Fatty acids** |  |  |  |
| Total fatty acids | -0.11 (0.05) | 0.05 | 0.04 |
| Degree of unsaturation | 0.26 (0.05) | 5.5*10^-7^ | 4.3*10^-5^ |
| DHA | 0.07 (0.06) | 0.17 | 0.09 |
| Linoleic acid | -0.03 (0.05) | 0.58 | 0.26 |
| Saturated fatty acids | -0.13 (0.06) | 0.02 | 0.02 |
| n-3 fatty acids | 0.04 (0.05) | 0.45 | 0.21 |
| n-6 fatty acids | -0.04 (0.05) | 0.50 | 0.23 |
| Monounsaturated fatty acids | -0.15 (0.05) | 0.008 | 0.01 |
| Polyunsaturated fatty acids | -0.02 (0.05) | 0.67 | 0.28 |
| **Fatty acid ratios** |  |  |  |
| Saturated fatty acids (%) | -0.06 (0.05) | 0.25 | 0.13 |
| Monounsaturated fatty acids (%) | -0.15 (0.05) | 0.006 | 0.007 |
| Polyunsaturated fatty acids (%) | 0.26 (0.05) | 1.1*10^-5^ | 4.3*10^-5^ |
| n-6 fatty acids (%) | 0.22 (0.05) | 5.0*10^-5^ | 2.2*10^-4^ |
| Linoleic acid (%) | 0.19 (0.05) | 4.8*10^-4^ | 0.001 |
| n-3 fatty acids (%) | 0.24 (0.05) | 8.9*10^-6^ | 2.1*10^-4^ |
| DHA (%) | 0.23 (0.05) | 2.8*10^-5^ | 2.1*10^-4^ |
| **Phospholipids** |  |  |  |
| Sphingomyelins | -0.03 (0.05) | 0.64 | 0.27 |
| Total cholines | 0.00 (0.05) | 0.91 | 0.35 |
| Phosphatidylcholines | -0.01 (0.05) | 0.78 | 0.32 |
| Total phosphoglycerides | -0.03 (0.05) | 0.63 | 0.27 |
| TG/PG | -0.17 (0.05) | 0.002 | 0.004 |
| Significance determined at p<0.05 and q<0.10. Linear regression, comparing metabolites (outcome) with fruit and vegetable consumption on a categorical scale (exposure), models adjusted for age, sex and study (pilot or larger sample) | | | |

**Supplementary Table 7.** Metabolite measures associated with alcohol consumption in the BCGP study sample (N=1,319)

|  | ß (SE) | p-value | q-value |
| --- | --- | --- | --- |
| **Composite lipid measures** |  |  |  |
| Total serum cholesterol | -0.08 (0.08) | 0.28 | 0.18 |
| Esterified cholesterol | -0.10 (0.08) | 0.18 | 0.14 |
| Free cholesterol | -0.08 (0.08) | 0.28 | 0.18 |
| Remnant cholesterol | 0.04 (0.08) | 0.62 | 0.30 |
| VLDL cholesterol | 0.09 (0.08) | 0.28 | 0.17 |
| LDL cholesterol | 0.16 (0.08) | 0.05 | 0.06 |
| HDL cholesterol | -0.38 (0.07) | 8.2*10^-8^ | 9.7*10^-7^ |
| HDL2 cholesterol | -0.34 (0.07) | 1.4*10^-6^ | 9.4*10^-6^ |
| HDL3 cholesterol | -0.25 (0.08) | 8.3*10^-4^ | 0.002 |
| Total triacylglycerol | 0.04 (0.08) | 0.57 | 0.29 |
| Total triacylglycerol in VLDL | 0.09 (0.08) | 0.25 | 0.16 |
| Total triacylglycerol in LDL | -0.04 (0.08) | 0.55 | 0.29 |
| Total triacylglycerol in HDL | -0.23 (0.08) | 0.002 | 0.004 |
| **Total lipids in lipoproteins** |  |  |  |
| Extremely large VLDL | 0.04 (0.08) | 0.60 | 0.30 |
| Very large VLDL | 0.10 (0.08) | 0.19 | 0.14 |
| Large VLDL | 0.10 (0.08) | 0.19 | 0.14 |
| Medium VLDL | 0.11 (0.08) | 0.16 | 0.14 |
| Small VLDL | 0.17 (0.08) | 0.03 | 0.05 |
| Very small VLDL | -0.10 (0.08) | 0.18 | 0.14 |
| IDL | -0.04 (0.08) | 0.60 | 0.30 |
| Large LDL | 0.11 (0.08) | 0.16 | 0.14 |
| Medium LDL | 0.17 (0.08) | 0.03 | 0.04 |
| Small LDL | 0.09 (0.08) | 0.25 | 0.16 |
| Very large HDL | -0.31 (0.07) | 4.2*10^-5^ | 1.6*10^-4^ |
| Large HDL | -0.28 (0.07) | 1.1*10^-4^ | 3.8*10^-4^ |
| Medium HDL | -0.40 (0.07) | 4.1*10^-9^ | 1.2*10^-7^ |
| Small HDL | -0.30 (0.07) | 3.7*10^-5^ | 1.5*10^-4^ |
| **Phospholipids in lipoproteins** |  |  |  |
| Extremely large VLDL | 0.04 (0.08) | 0.61 | 0.30 |
| Very large VLDL | 0.09 (0.08) | 0.22 | 0.15 |
| Large VLDL | 0.10 (0.08) | 0.19 | 0.14 |
| Medium VLDL | 0.11 (0.08) | 0.17 | 0.14 |
| Small VLDL | 0.17 (0.08) | 0.04 | 0.05 |
| Very small VLDL | -0.07 (0.07) | 0.34 | 0.20 |
| IDL | -0.04 (0.08) | 0.60 | 0.30 |
| Large LDL | 0.07 (0.08) | 0.41 | 0.23 |
| Medium LDL | 0.12 (0.08) | 0.12 | 0.12 |
| Small LDL | 0.03 (0.08) | 0.73 | 0.33 |
| Very large HDL | -0.25 (0.07) | 9.8*10^-4^ | 0.002 |
| Large HDL | -0.27 (0.07) | 2.8*10^-4^ | 7.7*10^-4^ |
| Medium HDL | -0.40 (0.07) | 4.7*10^-9^ | 1.2*10^-7^ |
| Small HDL | -0.34 (0.07) | 3.9*10^-6^ | 2.3*10^-5^ |
| **Cholesterol esters in lipoproteins** |  |  |  |
| Extremely large VLDL | 0.05 (0.08) | 0.53 | 0.28 |
| Very large VLDL | 0.13 (0.08) | 0.11 | 0.11 |
| Large VLDL | 0.10 (0.08) | 0.18 | 0.14 |
| Medium VLDL | 0.07 (0.08) | 0.36 | 0.21 |
| Small VLDL | 0.14 (0.08) | 0.08 | 0.09 |
| Very small VLDL | -0.13 (0.08) | 0.07 | 0.08 |
| IDL | -0.03 (0.08) | 0.68 | 0.31 |
| Large LDL | 0.14 (0.08) | 0.07 | 0.08 |
| Medium LDL | 0.23 (0.07) | 0.002 | 0.004 |
| Small LDL | 0.14 (0.07) | 0.07 | 0.08 |
| Very large HDL | -0.28 (0.07) | 2.7*10^-4^ | 7.6*10^-4^ |
| Large HDL | -0.25 (0.07) | 0.001 | 0.002 |
| Medium HDL | -0.36 (0.07) | 5.1*10^-7^ | 3.8*10^-6^ |
| Small HDL | -0.22 (0.07) | 0.002 | 0.004 |
| **Free cholesterol in lipoproteins** |  |  |  |
| Extremely large VLDL | 0.04 (0.08) | 0.64 | 0.31 |
| Very large VLDL | 0.09 (0.08) | 0.25 | 0.16 |
| Large VLDL | 0.09 (0.08) | 0.23 | 0.16 |
| Medium VLDL | 0.10 (0.08) | 0.20 | 0.14 |
| Small VLDL | 0.16 (0.08) | 0.04 | 0.06 |
| Very small VLDL | -0.07 (0.08) | 0.34 | 0.20 |
| IDL | -0.03 (0.08) | 0.61 | 0.30 |
| Large LDL | 0.11 (0.08) | 0.16 | 0.14 |
| Medium LDL | 0.16 (0.08) | 0.049 | 0.06 |
| Small LDL | 0.13 (0.08) | 0.10 | 0.10 |
| Very large HDL | -0.31 (0.07) | 3.2*10^-5^ | 1.4*10^-4^ |
| Large HDL | -0.28 (0.07) | 1.6*10^-4^ | 5.1*10^-4^ |
| Medium HDL | -0.39 (0.07) | 1.3*10^-8^ | 2.3*10^-7^ |
| Small HDL | -0.36 (0.07) | 2.7*10^-7^ | 2.5*10^-6^ |
| **Particles in lipoproteins** |  |  |  |
| Extremely large VLDL | 0.04 (0.08) | 0.62 | 0.30 |
| Very large VLDL | 0.10 (0.08) | 0.19 | 0.14 |
| Large VLDL | 0.10 (0.08) | 0.19 | 0.14 |
| Medium VLDL | 0.12 (0.08) | 0.14 | 0.13 |
| Small VLDL | 0.17 (0.08) | 0.03 | 0.05 |
| Very small VLDL | -0.10 (0.08) | 0.18 | 0.14 |
| IDL | -0.03 (0.08) | 0.65 | 0.31 |
| Large LDL | 0.12 (0.08) | 0.13 | 0.12 |
| Medium LDL | 0.19 (0.08) | 0.02 | 0.02 |
| Small LDL | 0.06 (0.04) | 0.22 | 0.15 |
| Very large HDL | -0.32 (0.07) | 2.4*10^-5^ | 1.1*10^-4^ |
| Large HDL | -0.29 (0.07) | 1.2*10^-4^ | 3.8*10^-4^ |
| Medium HDL | -0.41 (0.07) | 5.3*10^-9^ | 1.2*10^-7^ |
| Small HDL | -0.24 (0.07) | 9.7*10^-4^ | 0.002 |
| **Total cholesterol in lipoproteins** |  |  |  |
| Extremely large VLDL | 0.04 (0.08) | 0.57 | 0.29 |
| Very large VLDL | 0.11 (0.08) | 0.15 | 0.13 |
| Large VLDL | 0.10 (0.08) | 0.20 | 0.14 |
| Medium VLDL | 0.09 (0.08) | 0.25 | 0.16 |
| Small VLDL | 0.15 (0.08) | 0.06 | 0.07 |
| Very small VLDL | -0.12 (0.08) | 0.10 | 0.10 |
| IDL | -0.01 (0.03) | 0.66 | 0.31 |
| Large LDL | 0.14 (0.08) | 0.09 | 0.09 |
| Medium LDL | 0.21 (0.07) | 0.007 | 0.01 |
| Small LDL | 0.14 (0.08) | 0.07 | 0.08 |
| Very large HDL | -0.29 (0.07) | 9.5*10^-5^ | 3.4*10^-4^ |
| Large HDL | -0.26 (0.07) | 0.001 | 0.001 |
| Medium HDL | -0.36 (0.07) | 3.1*10^-7^ | 2.5*10^-6^ |
| Small HDL | -0.24 (0.07) | 7.8*10^-4^ | 0.002 |
| **Apolipoproteins** |  |  |  |
| Apolipoprotein A1 | -0.42 (0.07) | 6.0*10^-10^ | 5.6*10^-8^ |
| Apolipoprotein B | 0.10 (0.08) | 0.19 | 0.14 |
| Apo B/Apo A1 | 0.32 (0.07) | 2.2*10^-5^ | 1.0*10^-4^ |
| **Triacylglycerol in lipoproteins** |  |  |  |
| Extremely large VLDL | 0.04 (0.08) | 0.56 | 0.29 |
| Very large VLDL | 0.10 (0.08) | 0.18 | 0.14 |
| Large VLDL | 0.08 (0.08) | 0.27 | 0.17 |
| Medium VLDL | 0.10 (0.08) | 0.19 | 0.14 |
| Small VLDL | 0.14 (0.08) | 0.07 | 0.08 |
| Very small VLDL | -0.01 (0.08) | 0.89 | 0.38 |
| IDL | -0.07 (0.08) | 0.32 | 0.19 |
| Large LDL | -0.07 (0.08) | 0.35 | 0.20 |
| Medium LDL | -0.01 (0.08) | 0.89 | 0.38 |
| Small LDL | -0.02 (0.08) | 0.84 | 0.37 |
| Very large HDL | -0.28 (0.08) | 2.5*10^-4^ | 7.2*10^-4^ |
| Large HDL | -0.31 (0.07) | 2.2*10^-5^ | 1.0*10^-4^ |
| Medium HDL | -0.20 (0.08) | 0.01 | 0.02 |
| Small HDL | -0.01 (0.08) | 0.88 | 0.38 |
| **Lipoprotein particle size** |  |  |  |
| VLDL particle size | 0.13 (0.07) | 0.05 | 0.06 |
| LDL particle size | -0.06 (0.02) | 0.01 | 0.02 |
| HDL particle size | -0.23 (0.05) | 2.2*10^-5^ | 0.0001 |
| **Total cholesterol in lipoproteins (%)** |  |  |  |
| Very large VLDL | 0.09 (0.08) | 0.24 | 0.16 |
| Large VLDL | 0.04 (0.08) | 0.61 | 0.30 |
| Medium VLDL | -0.01 (0.08) | 0.88 | 0.38 |
| Small VLDL | 0.02 (0.08) | 0.83 | 0.37 |
| Very small VLDL | -0.13 (0.07) | 0.08 | 0.08 |
| IDL | 0.02 (0.07) | 0.85 | 0.37 |
| Large LDL | 0.23 (0.06) | 2.2*10^-4^ | 6.6*10^-4^ |
| Medium LDL | 0.33 (0.06) | 1.5*10^-8^ | 2.3*10^-7^ |
| Small LDL | 0.29 (0.06) | 5.6*10^-5^ | 3.1*10^-4^ |
| Very large HDL | 0.12 (0.08) | 0.14 | 0.13 |
| Large HDL | -0.09 (0.08) | 0.27 | 0.17 |
| Medium HDL | -0.11 (0.08) | 0.16 | 0.14 |
| Small HDL | 0.09 (0.07) | 0.22 | 0.15 |
| **Phospholipids in lipoproteins (%)** |  |  |  |
| Very large VLDL | 0.06 (0.08) | 0.47 | 0.25 |
| Large VLDL | -0.01 (0.08) | 0.89 | 0.38 |
| Medium VLDL | -0.04 (0.08) | 0.62 | 0.30 |
| Small VLDL | -0.05 (0.08) | 0.55 | 0.29 |
| Very small VLDL | 0.09 (0.07) | 0.22 | 0.15 |
| IDL | 0.00 (0.07) | 0.97 | 0.40 |
| Large LDL | -0.27 (0.07) | 6.4*10^-5^ | 2.4*10^-4^ |
| Medium LDL | -0.34 (0.06) | 8.8*10^-8^ | 9.7*10^-7^ |
| Small LDL | -0.30 (0.06) | 3.4*10^-6^ | 2.1*10^-4^ |
| Very large HDL | -0.15 (0.08) | 0.07 | 0.08 |
| Large HDL | 0.05 (0.08) | 0.53 | 0.28 |
| Medium HDL | 0.14 (0.07) | 0.06 | 0.07 |
| Small HDL | -0.19 (0.06) | 0.003 | 0.005 |
| **Cholesterol esters in lipoproteins (%)** | |  |  |
| Very large VLDL | 0.10 (0.08) | 0.22 | 0.15 |
| Large VLDL | 0.03 (0.08) | 0.68 | 0.31 |
| Medium VLDL | -0.01 (0.08) | 0.86 | 0.37 |
| Small VLDL | 0.02 (0.07) | 0.82 | 0.36 |
| Very small VLDL | -0.14 (0.07) | 0.06 | 0.07 |
| IDL | 0.02 (0.07) | 0.80 | 0.36 |
| Large LDL | 0.23 (0.07) | 5.5*10^-4^ | 0.001 |
| Medium LDL | 0.34 (0.06) | 3.0*10^-7^ | 2.5*10^-6^ |
| Small LDL | 0.23 (0.06) | 3.3*10^-4^ | 8.6*10^-4^ |
| Very large HDL | 0.00 (0.08) | 0.99 | 0.41 |
| Large HDL | -0.08 (0.08) | 0.31 | 0.18 |
| Medium HDL | -0.10 (0.08) | 0.23 | 0.16 |
| Small HDL | 0.10 (0.07) | 0.14 | 0.13 |
| **Free cholesterol in lipoproteins (%)** |  |  |  |
| Very large VLDL | 0.07 (0.08) | 0.40 | 0.23 |
| Large VLDL | 0.03 (0.08) | 0.65 | 0.31 |
| Medium VLDL | 0.00 (0.08) | 1.00 | 0.41 |
| Small VLDL | 0.00 (0.08) | 0.94 | 0.39 |
| Very small VLDL | 0.08 (0.07) | 0.29 | 0.18 |
| IDL | -0.01 (0.07) | 0.93 | 0.39 |
| Large LDL | 0.01 (0.07) | 0.89 | 0.38 |
| Medium LDL | -0.06 (0.07) | 0.41 | 0.23 |
| Small LDL | 0.14 (0.07) | 0.06 | 0.07 |
| Very large HDL | 0.18 (0.08) | 0.02 | 0.03 |
| Large HDL | -0.06 (0.08) | 0.44 | 0.24 |
| Medium HDL | -0.24 (0.07) | 0.0003 | 7.8*10^-4^ |
| Small HDL | -0.12 (0.07) | 0.08 | 0.08 |
| **Triacylglycerol in lipoproteins (%)** |  |  |  |
| Very large VLDL | 0.09 (0.08) | 0.23 | 0.16 |
| Large VLDL | -0.05 (0.08) | 0.52 | 0.28 |
| Medium VLDL | 0.02 (0.08) | 0.72 | 0.33 |
| Small VLDL | 0.00 (0.08) | 0.95 | 0.39 |
| Very small VLDL | 0.11 (0.08) | 0.15 | 0.13 |
| IDL | -0.04 (0.08) | 0.69 | 0.31 |
| Large LDL | -0.20 (0.08) | 0.02 | 0.02 |
| Medium LDL | -0.24 (0.08) | 0.003 | 0.005 |
| Small LDL | -0.11 (0.08) | 0.17 | 0.14 |
| Very large HDL | 0.08 (0.08) | 0.30 | 0.18 |
| Large HDL | 0.07 (0.08) | 0.40 | 0.23 |
| Medium HDL | 0.06 (0.08) | 0.50 | 0.27 |
| Small HDL | 0.12 (0.08) | 0.14 | 0.13 |
| **Branched-chain amino acids** |  |  |  |
| Isoleucine | 0.13 (0.08) | 0.07 | 0.08 |
| Leucine | 0.03 (0.08) | 0.57 | 0.29 |
| Valine | 0.09 (0.08) | 0.22 | 0.15 |
| **Aromatic amino acids** |  |  |  |
| Phenylalanine | 0.02 (0.08) | 0.74 | 0.33 |
| Tyrosine | -0.18 (0.08) | 0.02 | 0.04 |
| Histidine | -0.16 (0.08) | 0.05 | 0.06 |
| **Other amino acids** |  |  |  |
| Alanine | -0.01 (0.08) | 0.96 | 0.40 |
| Glutamine | -0.04 (0.07) | 0.60 | 0.30 |
| **Ketone bodies** |  |  |  |
| Acetoacetate | -0.08 (0.07) | 0.30 | 0.18 |
| 3-hydroxybutyrate | 0.06 (0.08) | 0.41 | 0.23 |
| **Miscellaneous** |  |  |  |
| Creatinine | 0.03 (0.07) | 0.68 | 0.31 |
| Albumin | -0.16 (0.06) | 0.007 | 0.01 |
| Acetate | 0.06 (0.08) | 0.52 | 0.28 |
| Citrate | 0.10 (0.08) | 0.18 | 0.14 |
| Glycoprotein acetyls | 0.19 (0.08) | 0.01 | 0.02 |
| **Fatty acids** |  |  |  |
| Total fatty acids | -0.05 (0.08) | 0.45 | 0.25 |
| Degree of unsaturation | -0.11 (0.08) | 0.13 | 0.12 |
| DHA | -0.14 (0.08) | 0.05 | 0.07 |
| Linoleic acid | -0.01 (0.08) | 0.82 | 0.36 |
| Saturated fatty acids | -0.07 (0.08) | 0.29 | 8 |
| n-3 fatty acids | -0.12 (0.08) | 0.11 | 0.10 |
| n-6 fatty acids | -0.06 (0.08) | 0.41 | 0.23 |
| Monounsaturated fatty acids | 0.00 (0.08) | 0.90 | 0.38 |
| Polyunsaturated fatty acids | -0.07 (0.08) | 0.30 | 0.18 |
| **Fatty acid ratios** |  |  |  |
| Saturated fatty acids (%) | -0.14 (0.08) | 0.03 | 0.05 |
| Monounsaturated fatty acids (%) | 0.11 (0.08) | 0.21 | 0.15 |
| Polyunsaturated fatty acids (%) | -0.05 (0.08) | 0.46 | 0.25 |
| n-6 fatty acids (%) | -0.01 (0.08) | 0.79 | 0.36 |
| Linoleic acid (%) | 0.07 (0.08) | 0.45 | 0.25 |
| n-3 fatty acids (%) | -0.13 (0.08) | 0.08 | 0.08 |
| DHA (%) | -0.15 (0.08) | 0.05 | 0.07 |
| **Phospholipids** |  |  |  |
| Sphingomyelins | -0.10 (0.08) | 0.16 | 0.14 |
| Total cholines | -0.23 (0.07) | 0.002 | 0.004 |
| Phosphatidylcholines | -0.24 (0.07) | 0.001 | 0.003 |
| Total phosphoglycerides | -0.25 (0.07) | 0.001 | 0.002 |
| TG/PG | 0.17 (0.08) | 0.03 | 0.04 |
| Significance determined at p<0.05 and q<0.10. Linear regression, comparing metabolites (outcome) with alcohol consumption on a categorical scale (exposure), models adjusted for age, sex and study (pilot or larger sample) | | | |

**Supplementary Table 8.** Metabolite measures associated with physical activity in the BCGP study sample (N=1,319)

|  | ß (SE) | p-value | q-value |
| --- | --- | --- | --- |
| **Composite lipid measures** |  |  |  |
| Total serum cholesterol | 0.13 (0.10) | 0.57 | 0.70 |
| Esterified cholesterol | 0.13 (0.10) | 0.27 | 0.59 |
| Free cholesterol | 0.11 (0.10) | 0.41 | 0.68 |
| Remnant cholesterol | 0.00 (0.11) | 0.87 | 0.77 |
| VLDL cholesterol | -0.05 (0.11) | 0.97 | 0.79 |
| LDL cholesterol | 0.02 (0.10) | 0.00 | 0.13 |
| HDL cholesterol | 0.25 (0.10) | 0.01 | 0.18 |
| HDL2 cholesterol | 0.22 (0.10) | 0.09 | 0.35 |
| HDL3 cholesterol | 0.17 (0.10) | 0.19 | 0.48 |
| Total triacylglycerol | -0.05 (0.11) | 0.23 | 0.55 |
| Total triacylglycerol in VLDL | -0.09 (0.10) | 0.76 | 0.75 |
| Total triacylglycerol in LDL | 0.07 (0.12) | 0.64 | 0.72 |
| Total triacylglycerol in HDL | 0.08 (0.10) | 0.13 | 0.41 |
| **Total lipids in lipoproteins** |  |  |  |
| Extremely large VLDL | -0.03 (0.11) | 0.48 | 0.68 |
| Very large VLDL | -0.07 (0.11) | 0.29 | 0.60 |
| Large VLDL | -0.09 (0.11) | 0.24 | 0.55 |
| Medium VLDL | -0.05 (0.11) | 0.51 | 0.68 |
| Small VLDL | -0.08 (0.11) | 0.39 | 0.68 |
| Very small VLDL | 0.05 (0.10) | 0.58 | 0.70 |
| IDL | 0.06 (0.10) | 0.47 | 0.68 |
| Large LDL | 0.04 (0.11) | 0.71 | 0.73 |
| Medium LDL | 0.02 (0.10) | 0.96 | 0.79 |
| Small LDL | 0.07 (0.11) | 0.61 | 0.71 |
| Very large HDL | 0.15 (0.10) | 0.05 | 0.30 |
| Large HDL | 0.16 (0.10) | 0.04 | 0.28 |
| Medium HDL | 0.23 (0.09) | 0.00 | 0.13 |
| Small HDL | 0.19 (0.09) | 0.06 | 0.33 |
| **Phospholipids in lipoproteins** |  |  |  |
| Extremely large VLDL | -0.05 (0.11) | 0.44 | 0.68 |
| Very large VLDL | -0.05 (0.10) | 0.40 | 0.68 |
| Large VLDL | -0.08 (0.10) | 0.29 | 0.60 |
| Medium VLDL | -0.04 (0.11) | 0.59 | 0.70 |
| Small VLDL | -0.06 (0.11) | 0.54 | 0.69 |
| Very small VLDL | 0.06 (0.10) | 0.49 | 0.68 |
| IDL | 0.06 (0.04) | 0.46 | 0.68 |
| Large LDL | 0.06 (0.10) | 0.54 | 0.69 |
| Medium LDL | 0.05 (0.10) | 0.71 | 0.73 |
| Small LDL | 0.10 (0.10) | 0.39 | 0.68 |
| Very large HDL | 0.13 (0.10) | 0.08 | 0.35 |
| Large HDL | 0.14 (0.10) | 0.06 | 0.34 |
| Medium HDL | 0.20 (0.09) | 0.01 | 0.18 |
| Small HDL | 0.18 (0.10) | 0.03 | 0.27 |
| **Cholesterol esters in lipoproteins** |  |  |  |
| Extremely large VLDL | -0.04 (0.11) | 0.50 | 0.68 |
| Very large VLDL | -0.08 (0.11) | 0.30 | 0.60 |
| Large VLDL | -0.13 (0.11) | 0.15 | 0.43 |
| Medium VLDL | -0.04 (0.10) | 0.83 | 0.76 |
| Small VLDL | -0.06 (0.11) | 0.63 | 0.71 |
| Very small VLDL | 0.06 (0.11) | 0.46 | 0.68 |
| IDL | 0.05 (0.10) | 0.51 | 0.68 |
| Large LDL | 0.03 (0.10) | 0.84 | 0.76 |
| Medium LDL | 0.00 (0.10) | 0.81 | 0.76 |
| Small LDL | 0.06 (0.10) | 0.73 | 0.74 |
| Very large HDL | 0.10 (0.10) | 0.14 | 0.43 |
| Large HDL | 0.14 (0.10) | 0.08 | 0.35 |
| Medium HDL | 0.24 (0.10) | 0.01 | 0.13 |
| Small HDL | 0.20 (0.09) | 0.02 | 0.21 |
| **Free cholesterol in lipoproteins** |  |  |  |
| Extremely large VLDL | -0.03 (0.11) | 0.51 | 0.68 |
| Very large VLDL | -0.11 (0.11) | 0.19 | 0.48 |
| Large VLDL | -0.13 (0.11) | 0.12 | 0.41 |
| Medium VLDL | -0.05 (0.11) | 0.54 | 0.69 |
| Small VLDL | -0.07 (0.11) | 0.50 | 0.68 |
| Very small VLDL | 0.03 (0.12) | 0.58 | 0.70 |
| IDL | 0.06 (0.11) | 0.45 | 0.68 |
| Large LDL | 0.03 (0.11) | 0.68 | 0.72 |
| Medium LDL | 0.01 (0.11) | 0.92 | 0.78 |
| Small LDL | 0.02 (0.10) | 0.84 | 0.76 |
| Very large HDL | 0.15 (0.10) | 0.07 | 0.34 |
| Large HDL | 0.15 (0.10) | 0.05 | 0.30 |
| Medium HDL | 0.23 (0.09) | 0.00 | 0.13 |
| Small HDL | 0.22 (0.09) | 0.40 | 0.68 |
| **Particles in lipoproteins** |  |  |  |
| Extremely large VLDL | -0.03 (0.11) | 0.49 | 0.68 |
| Very large VLDL | -0.07 (0.11) | 0.30 | 0.60 |
| Large VLDL | -0.08 (0.11) | 0.26 | 0.59 |
| Medium VLDL | -0.06 (0.11) | 0.45 | 0.68 |
| Small VLDL | -0.09 (0.11) | 0.33 | 0.62 |
| Very small VLDL | 0.05 (0.11) | 0.62 | 0.71 |
| IDL | 0.06 (0.10) | 0.48 | 0.68 |
| Large LDL | 0.04 (0.10) | 0.75 | 0.75 |
| Medium LDL | 0.01 (0.10) | 0.96 | 0.79 |
| Small LDL | 0.04 (0.11) | 0.66 | 0.72 |
| Very large HDL | 0.15 (0.10) | 0.07 | 0.35 |
| Large HDL | 0.17 (0.10) | 0.03 | 0.28 |
| Medium HDL | 0.25 (0.10) | 0.00 | 0.13 |
| Small HDL | 0.20 (0.09) | 0.05 | 0.30 |
| **Total cholesterol in lipoproteins** |  |  |  |
| Extremely large VLDL | -0.03 (0.11) | 0.51 | 0.68 |
| Very large VLDL | -0.09 (0.11) | 0.27 | 0.59 |
| Large VLDL | -0.13 (0.11) | 0.14 | 0.42 |
| Medium VLDL | -0.04 (0.11) | 0.78 | 0.76 |
| Small VLDL | -0.06 (0.11) | 0.58 | 0.70 |
| Very small VLDL | 0.06 (0.10) | 0.48 | 0.68 |
| IDL | 0.02 (0.10) | 0.50 | 0.68 |
| Large LDL | 0.03 (0.10) | 0.80 | 0.76 |
| Medium LDL | 0.00 (0.10) | 0.89 | 0.77 |
| Small LDL | 0.04 (0.10) | 0.77 | 0.76 |
| Very large HDL | 0.13 (0.10) | 0.09 | 0.35 |
| Large HDL | 0.15 (0.10) | 0.06 | 0.33 |
| Medium HDL | 0.24 (0.10) | 0.01 | 0.13 |
| Small HDL | 0.21 (0.09) | 0.04 | 0.28 |
| **Apolipoproteins** |  |  |  |
| Apolipoprotein A1 | 0.28 (0.09) | 0.85 | 0.76 |
| Apolipoprotein B | 0.00 (0.11) | 0.10 | 0.37 |
| Apo B/Apo A1 | -0.15 (0.11) | 0.10 | 0.37 |
| **Triacylglycerol in lipoproteins** |  |  |  |
| Extremely large VLDL | -0.04 (0.11) | 0.44 | 0.68 |
| Very large VLDL | -0.07 (0.10) | 0.33 | 0.62 |
| Large VLDL | -0.08 (0.11) | 0.28 | 0.60 |
| Medium VLDL | -0.10 (0.11) | 0.22 | 0.53 |
| Small VLDL | -0.15 (0.11) | 0.09 | 0.35 |
| Very small VLDL | -0.03 (0.11) | 0.64 | 0.72 |
| IDL | 0.05 (0.10) | 0.77 | 0.76 |
| Large LDL | 0.08 (0.10) | 0.67 | 0.72 |
| Medium LDL | 0.07 (0.06) | 0.81 | 0.76 |
| Small LDL | 0.06 (0.11) | 0.93 | 0.79 |
| Very large HDL | 0.04 (0.10) | 0.62 | 0.71 |
| Large HDL | 0.14 (0.10) | 0.12 | 0.41 |
| Medium HDL | 0.04 (0.11) | 0.89 | 0.77 |
| Small HDL | -0.06 (0.11) | 0.83 | 0.76 |
| **Lipoprotein particle size** |  |  |  |
| VLDL particle size | -0.09 (0.09) | 0.63 | 0.71 |
| LDL particle size | 0.00 (0.03) | 0.03 | 0.28 |
| HDL particle size | 0.13 (0.07) | 0.21 | 0.53 |
| **Total cholesterol in lipoproteins (%)** |  |  |  |
| Very large VLDL | -0.09 (0.10) | 0.68 | 0.72 |
| Large VLDL | -0.19 (0.10) | 0.17 | 0.46 |
| Medium VLDL | 0.00 (0.10) | 0.80 | 0.76 |
| Small VLDL | -0.02 (0.10) | 0.66 | 0.72 |
| Very small VLDL | 0.04 (0.10) | 0.49 | 0.68 |
| IDL | -0.01 (0.09) | 0.89 | 0.77 |
| Large LDL | -0.06 (0.08) | 0.46 | 0.68 |
| Medium LDL | -0.12 (0.07) | 0.17 | 0.46 |
| Small LDL | -0.13 (0.08) | 0.41 | 0.68 |
| Very large HDL | -0.25 (0.10) | 0.22 | 0.53 |
| Large HDL | 0.03 (0.11) | 0.63 | 0.71 |
| Medium HDL | 0.15 (0.11) | 0.11 | 0.38 |
| Small HDL | 0.04 (0.10) | 0.59 | 0.70 |
| **Phospholipids in lipoproteins (%)** |  |  |  |
| Very large VLDL | -0.01 (0.10) | 0.44 | 0.68 |
| Large VLDL | -0.01 (0.10) | 0.08 | 0.35 |
| Medium VLDL | 0.00 (0.10) | 0.68 | 0.72 |
| Small VLDL | 0.01 (0.10) | 0.91 | 0.78 |
| Very small VLDL | 0.02 (0.09) | 0.44 | 0.68 |
| IDL | 0.00 (0.09) | 0.83 | 0.76 |
| Large LDL | 0.08 (0.09) | 0.49 | 0.68 |
| Medium LDL | 0.16 (0.08) | 0.08 | 0.35 |
| Small LDL | 0.18 (0.08) | 0.15 | 0.43 |
| Very large HDL | 0.00 (0.10) | 0.04 | 0.28 |
| Large HDL | -0.09 (0.11) | 0.57 | 0.70 |
| Medium HDL | -0.20 (0.10) | 0.09 | 0.35 |
| Small HDL | 0.01 (0.08) | 0.53 | 0.69 |
| **Cholesterol esters in lipoproteins (%)** | |  |  |
| Very large VLDL | -0.06 (0.10) | 0.13 | 0.41 |
| Large VLDL | -0.17 (0.10) | 0.05 | 0.30 |
| Medium VLDL | -0.02 (0.10) | 0.53 | 0.69 |
| Small VLDL | -0.06 (0.09) | 0.93 | 0.79 |
| Very small VLDL | 0.04 (0.10) | 0.73 | 0.74 |
| IDL | -0.01 (0.09) | 0.70 | 0.73 |
| Large LDL | -0.05 (0.08) | 0.88 | 0.77 |
| Medium LDL | -0.09 (0.08) | 0.89 | 0.77 |
| Small LDL | -0.04 (0.08) | 0.37 | 0.67 |
| Very large HDL | -0.19 (0.11) | 0.01 | 0.18 |
| Large HDL | 0.02 (0.11) | 0.84 | 0.76 |
| Medium HDL | 0.14 (0.11) | 0.02 | 0.21 |
| Small HDL | 0.04 (0.10) | 0.36 | 0.66 |
| **Free cholesterol in lipoproteins (%)** |  |  |  |
| Very large VLDL | -0.14 (0.10) | 0.42 | 0.68 |
| Large VLDL | -0.18 (0.10) | 0.68 | 0.72 |
| Medium VLDL | -0.07 (0.10) | 0.07 | 0.35 |
| Small VLDL | -0.04 (0.10) | 0.01 | 0.18 |
| Very small VLDL | 0.02 (0.10) | 0.18 | 0.47 |
| IDL | 0.00 (0.09) | 0.67 | 0.72 |
| Large LDL | -0.03 (0.10) | 0.95 | 0.79 |
| Medium LDL | -0.03 (0.10) | 0.81 | 0.76 |
| Small LDL | -0.13 (0.10) | 0.68 | 0.72 |
| Very large HDL | -0.27 (0.10) | 0.03 | 0.27 |
| Large HDL | 0.00 (0.11) | 0.27 | 0.59 |
| Medium HDL | 0.17 (0.09) | 0.23 | 0.55 |
| Small HDL | 0.05 (0.09) | 0.10 | 0.38 |
| **Triacylglycerol in lipoproteins (%)** |  |  |  |
| Very large VLDL | -0.06 (0.10) | 0.98 | 0.80 |
| Large VLDL | -0.03 (0.10) | 0.99 | 0.80 |
| Medium VLDL | -0.14 (0.10) | 0.82 | 0.76 |
| Small VLDL | -0.21 (0.10) | 0.71 | 0.73 |
| Very small VLDL | -0.09 (0.10) | 0.96 | 0.79 |
| IDL | -0.01 (0.11) | 0.28 | 0.60 |
| Large LDL | 0.03 (0.11) | 0.02 | 0.24 |
| Medium LDL | 0.05 (0.10) | 0.01 | 0.18 |
| Small LDL | 0.00 (0.11) | 0.63 | 0.71 |
| Very large HDL | -0.22 (0.10) | 0.51 | 0.68 |
| Large HDL | -0.10 (0.11) | 0.02 | 0.21 |
| Medium HDL | -0.10 (0.11) | 0.94 | 0.79 |
| Small HDL | -0.15 (0.11) | 0.13 | 0.41 |
| **Branched-chain amino acids** |  |  |  |
| Isoleucine | -0.07 (0.11) | 0.29 | 0.60 |
| Leucine | -0.06 (0.10) | 0.33 | 0.62 |
| Valine | -0.08 (0.10) | 0.31 | 0.60 |
| **Aromatic amino acids** |  |  |  |
| Phenylalanine | 0.02 (0.10) | 0.95 | 0.79 |
| Tyrosine | -0.02 (0.10) | 0.52 | 0.69 |
| Histidine | 0.09 (0.10) | 0.53 | 0.69 |
| **Other amino acids** |  |  |  |
| Alanine | -0.11 (0.10) | 0.13 | 0.41 |
| Glutamine | 0.01 (0.10) | 0.95 | 0.79 |
| **Ketone bodies** |  |  |  |
| Acetoacetate | 0.20 (0.10) | 0.02 | 0.21 |
| 3-hydroxybutyrate | 0.18 (0.10) | 0.05 | 0.30 |
| **Miscellaneous** |  |  |  |
| Creatinine | 0.01 (0.09) | 0.87 | 0.77 |
| Albumin | 0.10 (0.08) | 0.24 | 0.55 |
| Acetate | 0.43 (0.11) | 0.00 | 0.04 |
| Citrate | 0.16 (0.10) | 0.08 | 0.35 |
| Glycoprotein acetyls | -0.11 (0.11) | 0.17 | 0.46 |
| **Fatty acids** |  |  |  |
| Total fatty acids | 0.10 (0.11) | 0.57 | 0.70 |
| Degree of unsaturation | 0.01 (0.10) | 0.57 | 0.70 |
| DHA | 0.02 (0.10) | 0.84 | 0.76 |
| Linoleic acid | 0.12 (0.11) | 0.36 | 0.66 |
| Saturated fatty acids | 0.05 (0.09) | 0.85 | 0.76 |
| n-3 fatty acids | 0.06 (0.11) | 0.71 | 0.73 |
| n-6 fatty acids | 0.13 (0.11) | 0.29 | 0.60 |
| Monounsaturated fatty acids | -0.04 (0.10) | 0.46 | 0.68 |
| Polyunsaturated fatty acids | 0.04 (0.10) | 0.37 | 0.66 |
| **Fatty acid ratios** |  |  |  |
| Saturated fatty acids (%) | 0.10 (0.11) | 0.60 | 0.70 |
| Monounsaturated fatty acids (%) | 0.06 (0.11) | 0.83 | 0.76 |
| Polyunsaturated fatty acids (%) | 0.12 (0.11) | 0.34 | 0.63 |
| n-6 fatty acids (%) | 0.05 (0.10) | 0.31 | 0.60 |
| Linoleic acid (%) | 0.05 (0.11) | 0.41 | 0.68 |
| n-3 fatty acids (%) | -0.04 (0.10) | 0.88 | 0.77 |
| DHA (%) | -0.06 (0.10) | 0.78 | 0.76 |
| **Phospholipids** |  |  |  |
| Sphingomyelins | 0.15 (0.10) | 0.13 | 0.41 |
| Total cholines | 0.16 (0.10) | 0.00 | 0.08 |
| Phosphatidylcholines | 0.16 (0.10) | 0.16 | 0.45 |
| Total phosphoglycerides | 0.17 (0.10) | 0.15 | 0.43 |
| TG/PG | -0.11 (0.11) | 0.15 | 0.43 |
| Significance determined at p<0.05 and q<0.10. Linear regression, comparing metabolites (outcome) with physical activity on a continuous scale (exposure), models adjusted for age, sex and study (pilot or larger sample) | | | |
